# Molecular features of TNBC govern heterogeneity in the response to radiation and autophagy inhibition

**DOI:** 10.1038/s41419-025-07873-w

**Published:** 2025-07-21

**Authors:** Patrick Fischer, Maximilian Schmid, Anna Ohradanova-Repic, Rebecca Schneeweiss, Jana Hadatsch, Odysseus Grünert, Johannes Benedum, Anna Röhrer, Felix Staudinger, Philipp Schatzlmaier, Niccolo Bragato, Sandra Barna, Magdalena Engl, Ava Kleinwächter, Dietmar Georg, Joachim Widder, Sylvia Kerschbaum-Gruber, Dea Slade

**Affiliations:** 1 Department of Radiation Oncology, Medical University of Vienna, Währinger Gürtel 18-20, 1090 Vienna, Austria; 2 Comprehensive Cancer Center, Medical University of Vienna, Spitalgasse 23, 1090 Vienna, Austria; 3 Max Perutz Labs, Vienna Biocenter Campus (VBC), Dr.-Bohr-Gasse 9, 1030 Vienna, Austria; 4 Medical University of Vienna, Center for Medical Biochemistry, Dr.-Bohr-Gasse 9, 1030 Vienna, Austria; 5 MedAustron Ion Therapy Center, Wiener Neustadt, Austria; 6 Medical University of Vienna, Center for Pathophysiology, Infectiology and Immunology, Institute for Hygiene and Applied Immunology, Lazarettgasse 19, 1090 Vienna, Austria; 7 Vienna Biocenter PhD Program, a Doctoral School of the University of Vienna and the Medical University of Vienna, 1030 Vienna, Austria

**Keywords:** Breast cancer, Macroautophagy, Pattern recognition receptors, Radiotherapy

## Abstract

Triple negative breast cancer (TNBC) is a heterogeneous and a highly aggressive type of breast cancer. Standard of care for TNBC patients includes surgery, radio-, chemo- and immunotherapy, depending on the stage of the disease. Immunotherapy is ineffective as monotherapy but can be enhanced with taxane chemotherapy or radiotherapy. Radiation can stimulate the immune system by activating the type I interferon (IFN-I) response through cGAS-STING signaling, which recognizes cytosolic double-stranded DNA (dsDNA). Cytosolic dsDNA can be cleared by autophagy, thereby preventing activation of cGAS-STING signaling. Autophagy inhibition was therefore proposed to potentiate the immunostimulatory effects of radiation. Here we show that different molecular features of TNBC cell lines influence the effect of X-ray and carbon ion (C-ion) irradiation and autophagy inhibition on immunogenic signaling. MDA-MB-468, with low basal autophagy and high cytosolic dsDNA, activates the IFN-I response after radiation. In contrast, MDA-MB-231, characterized by high autophagy rates and low cytosolic dsDNA, induces NF-κB signaling and CXCL10 expression upon autophagy inhibition with the VPS34 inhibitor SAR405. Autophagy inhibition in TNBC cells triggers a stronger activation of innate immune cells (monocytes, natural killer cells and dendritic cells) compared to radiation. In BRCA1-mutated MDA-MB-436 cells, C-ion irradiation was more potent compared to X-rays in inducing the NF-κB-driven immunogenic response but failed to activate immune cells. Upregulation of PD-L1 by X-rays, and especially C-ions, may contribute to reduced immune cell activation, underscoring the need for combination strategies with immune checkpoint blockade. Collectively, our study highlights the NF-κB-driven immunostimulatory effects of autophagy inhibition and the importance of understanding the molecular heterogeneity in TNBC with regard to autophagy rates, IFN-I and NF-κB signaling when designing effective treatments that target these pathways.

## Introduction

Breast cancer is the most frequent malignancy in women with approximately 2.1 million newly diagnosed cases every year. Triple negative breast cancer (TNBC) is a subtype of epithelial breast cancer and accounts for approximately 10-15% of all breast cancer cases [1]. Among the most frequent mutations are tumor protein p53 (TP53), phosphatidylinositol-4,5-bisphosphate 3-kinase catalytic subunit alpha (PIK3CA) and breast cancer gene 1/2 (BRCA1/2) [2]. TNBC is characterized by the lack of the oestrogen receptor (ER) and progesterone receptor (PR) expression, as well as lack of overexpression of human epidermal growth factor 2 (HER2). Given its highly aggressive behavior, TNBC is associated with poor prognosis and limited treatment options. Cytotoxic chemotherapy with anthracyclines and taxol is the only established systemic treatment option for metastatic TNBC, which is, however, associated with high toxicity and resistance [3]. Immune checkpoint blockade (ICB) with pembrolizumab (anti-PD-1) combined with standard chemotherapy is an FDA-approved regimen for patients with metastatic and early-stage TNBC.

As a genotoxic agent, radiation has the potential to enhance ICB through its immunostimulatory effects on antigen-presenting cells and tumor-infiltrating lymphocytes, also referred to as radiation-induced immunogenicity [4, 5]. Mechanistically, radiation induces accumulation of double-stranded DNA (dsDNA) in the cytosol, which may originate from damaged mitochondria [6]. Cytosolic dsDNA can be detected by the nucleic acid sensor Cyclic GMP-AMP synthase (cGAS), which produces cGAMP and activates Stimulator of Interferon Genes (STING) [7, 8]. STING promotes TANK-binding kinase 1 (TBK1)-mediated phosphorylation and nuclear translocation of the Interferon regulatory factor 3 (IRF3) transcription factor, which triggers the type-I interferon (IFN-I) response. Interferon beta 1 (IFNB1) is secreted and binds to the interferon-α/β receptor (IFNAR) to activate the Janus kinase/signal transducer and activator of transcription (JAK/STAT) signaling pathway. Lastly, heterodimers of phosphorylated STAT1 and STAT2 traffic into the nucleus to form a complex on DNA together with IRF9 (ISGF3) and induce transcription of interferon-stimulated genes (ISGs) such as chemokines and cytokines that can attract and activate cytotoxic T cells [9].

STING can also induce the expression of cytokines through the NF-κB pathway in a TBK1/I-kappa-B kinase epsilon (IKKε)-dependent or -independent manner [10, 11]. Upon activation of STING, TBK1 and IKKε can both activate the IKK complex composed of two catalytic subunits IKKα and IKKβ and the regulatory subunit NF-kappa-B essential modulator (NEMO). Activation of the IKK complex results in phosphorylation of the inhibitory protein IkB, followed by its ubiquitination and degradation in the proteasome, allowing NF-κB p65 (RelA)/p50 to migrate to the nucleus and induce the expression of IFNB1 but also proinflammatory cytokines such as Interleukin 6 (IL-6) that can promote tumor proliferation [10, 12, 13] and immune evasion [14]. STING can also activate non-canonical NF-κB signaling via RelB/p52 in a TBK1-dependent or -independent manner [12, 15]. Non-canonical NF-κB signaling was shown to counteract radiation-induced canonical STING-NF-κB signaling and IFN-I production [15, 16].

While photon (X-ray) radiotherapy is conventionally used for cancer treatment, particle therapy with carbon ions (C-ions) may improve the clinical outcome due to their higher ionization density (quantified by the Linear Energy Transfer – LET), improved sparing of healthy tissue and a stronger anti-tumor immunogenic response, which underlines its potential for combination therapy with ICB [17]. The stronger immunogenic potential of C-ions stems from clustered DNA damage and induction of error-prone DNA repair pathways, which increases the mutational burden and antigenicity of the tumor. Moreover, C-ions were shown to induce a stronger release of damage-associated molecular patterns that activate antigen-presenting cells [18].

Various factors can modulate immunostimulatory effects of radiation, including mutations in DNA repair genes such as *BRCA*, the expression and activation of the cGAS-STING, IFN-I and NF-κB signaling pathways, as well as autophagy. *BRCA* mutations were shown to enhance the immunogenic effects of Poly (ADP-ribose) polymerase (PARP) inhibitors and were shown to sensitize tumors to radiation in combination with PARP inhibitors [19, 20]. STING is often downregulated in cancer, which correlates with a worse clinical outcome [21, 22]. Oncogenic KRAS mutations activate the NF-κB pathway, which can promote tumor proliferation [23, 24]. Autophagy acts as a recycling mechanism that degrades potentially dangerous cellular materials ranging from misfolded proteins and protein aggregates to whole damaged mitochondria and intracellular bacteria [25]. Autophagy was shown to eliminate cytosolic dsDNA and thereby act as a negative regulator of radiation-induced immunogenicity [26, 27]. Autophagy inhibition can activate anti-tumor immune response by preventing the clearance of cytosolic dsDNA and may elicit synergistic anti-tumor effects when combined with radiation and immunotherapy [28].

Here we show that three different TNBC cell lines, MDA-MB-468, MDA-MB-436 and MDA-MB-231, show distinct responses to various types of radiation (X-rays and C-ions) and autophagy inhibitors due to inherent differences in signaling mechanisms and autophagy rates. Of the three cell lines, we found that MDA-MB-468 accumulates highest levels of cytosolic dsDNA due to lowest autophagy rates and can mount an IFN-I response after radiation or taxol treatment. In contrast to MDA-MB-468, MDA-MB-436 and MDA-MB-231 showed low levels of cytosolic dsDNA and high autophagy rates. The BRCA1-mutated MDA-MB-436 cell line induced an IFN-I response to radiation and showed a stronger response to C-ions compared to conventional X-ray irradiation. The KRAS-mutated MDA-MB-231 did not respond to radiation but showed the strongest immunogenic response to autophagy inhibition by inducing the expression of the chemokine *C-X-C motif chemokine ligand 10 (CXCL10)* and the proinflammatory cytokine *IL-6* via STING-NF-κB signaling. In all cell lines, autophagy inhibition with the VPS34 inhibitor SAR405 induced pTBK1 sequestration, shifting the signaling from IFN-I to NF-κB. Supernatants from MDA-MB-231 and MDA-MB-468 treated with the autophagy inhibitor activated innate immune cells including monocytes, natural killer (NK) cells and dendritic cells (DCs). Despite a stronger induction of IFNB1 after C-ions, only the supernatants from X-ray-irradiated MDA-MB-436 activated the innate immune cells. Overall, our data highlight the need for understanding molecular alterations associated with different TNBC subtypes in order to develop personalized targeted therapies.

## Results

### Irradiation releases mitochondrial dsDNA into the cytoplasm

**Fig. 1:**
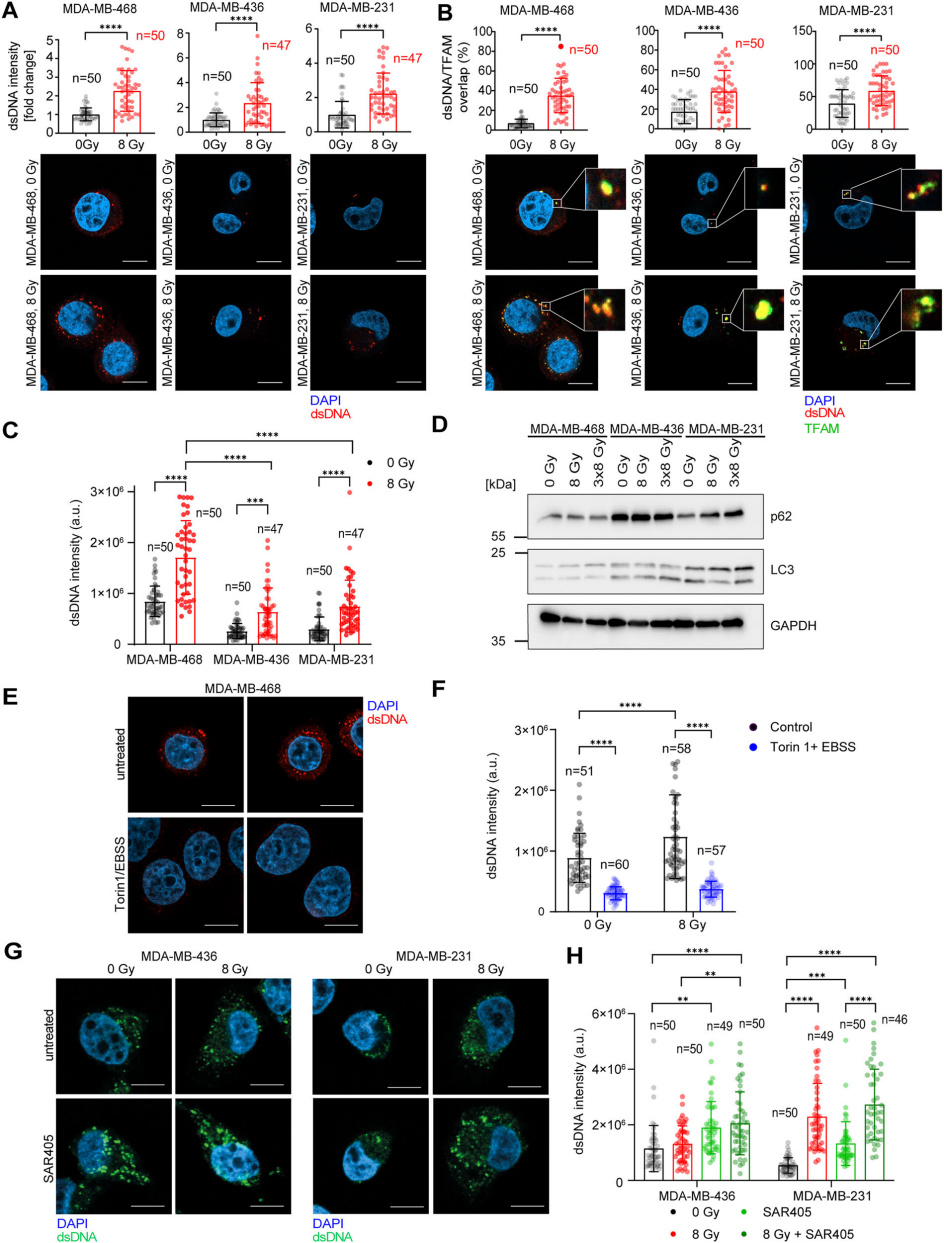
Autophagy rates correlate with the levels of cytosolic dsDNA in TNBC cell lines. **A** Quantifications of dsDNA intensity of cytosolic dsDNA and representative images of MDA-MB-468, MDA-MB-436 and MDA-MB-231 control cells and cells exposed to 8 Gy (X-ray) irradiation, harvested after 24 hours, and stained with anti-dsDNA antibody (red) and nucleus-stained with DAPI (blue). Scale bar: 10 μm. **B** Quantifications of cytosolic dsDNA-TFAM colocalization and representative images of MDA-MB-468, MDA-MB-436 and MDA-MB-231 control cells and cells exposed to 8 Gy (X-ray) irradiation, harvested after 24 hours, and stained with anti-dsDNA antibody (red), TFAM (green) and nucleus-stained with DAPI (blue). Scale bar: 10 μm. **C** Quantifications of dsDNA intensity of cytosolic dsDNA in MDA-MB-468, MDA-MB-436 and MDA-MB-231. **D** Western blots of MDA-MB-468, MDA-MB-436 and MDA-MB-231 exposed to 8 Gy and 3×8 Gy irradiation (X-rays). Cells were harvested 24 h after the last irradiation. GAPDH served as a loading control. **E** Representative images of MDA-MB-468 control cells and cells exposed to 8 Gy (X-ray) irradiation and harvested after 24 h (left) or cells treated with Torin1/EBSS for 8 h before harvesting (right). Scale bar: 10 μm. **F** Quantifications of dsDNA intensity of MDA-MB-468 cells under (**E**). **G** Representative images of MDA-MB-436 and MDA-MB-231 control cells and cells exposed to 8 Gy (X-ray) irradiation and harvested after 48 h or cells treated with the autophagy inhibitor SAR405 (3 μM for 48 h). Scale bar: 10 μm. **H** Quantifications of dsDNA intensity of MDA-MB-436 and MDA-MB-231 cells under (**G**). The bars and error bars represent mean values ± standard deviation. Three biologically independent experiments were performed. *P* values were calculated with two-way ANOVA (* ≤ 0.05; **≤ 0.01; ***≤ 0.005, ****≤0.0001).

Previous studies have shown that irradiation can lead to the accumulation of dsDNA in the cytosol of breast cancer cells [26, 29]. To assess radiation-induced cytosolic dsDNA accumulation in human TNBC cells, MDA-MB-468, MDA-MB-436 and MDA-MB-231 were irradiated with a single dose of 8 Gy X-rays and fixed after 24 h. Subsequently, the intensity of cytosolic dsDNA was quantified by immunofluorescence. All three investigated cell lines showed radiation-induced accumulation of cytosolic dsDNA (Fig. 1A). To assess whether cytosolic dsDNA originates from mitochondria as previously reported [26], we performed co-localization experiments with the mitochondrial transcription factor A (TFAM), known to bind mtDNA and recently reported to act as an autophagy receptor [27]. Indeed, radiation led to increased colocalization of TFAM and dsDNA in the cytoplasm of all three human TNBC cell lines (Fig. 1B). Taken together, mtDNA is released into the cytosol upon 8 Gy single dose radiation in human TNBC cell lines.

### TNBC cell lines show a delayed immunogenic response after radiation and taxol

Cytosolic dsDNA is recognized by the DNA sensor cGAS, which activates downstream signaling leading to the production of type I IFNs and proinflammatory chemokines and cytokines such as CXCL10 and IL-6 [7]. Given that all three cell lines showed increased levels of cytosolic dsDNA 24 h after radiation, we analyzed the effect of 8 Gy single dose and 3×8 Gy fractionated X-ray irradiation on the induction of the cytokine expression. The hypofractionation regimen of 3×8 Gy was previously shown to be more immunogenic in breast cancer cells compared to a single dose of 20 Gy [29]. We performed a time course analysis and collected the samples on five consecutive days after 8 Gy single dose and on three consecutive days after 3×8 Gy (Supplementary Fig. 1). In addition, cells were treated for up to three days with the chemotherapeutic drug taxol, which was reported to activate the IFN-I response in TNBC [30]. The mRNA levels of *IFNB1*, *CXCL10*, *PD-L1* and *IL-6* were measured by RT-qPCR. *IFNB1* expression can be induced by IRF3 or NF-κB transcription factors, *CXCL10* and *PD-L1* are ISGs, *CXCL10* can also be induced directly by NF-κB in an IFNB1-independent manner, while *IL-6* is induced directly by NF-κB [31–33].

We observed a delayed induction of cytokine expression in all three TNBC cell lines between days 2 and 4 after the treatment, in accordance with previous reports showing that cell cycle progression is required for accumulation of immunostimulatory cytosolic dsDNA [34]. Cytokine induction was the strongest in MDA-MB-468 and the weakest in MDA-MB-436 (Supplementary Fig. 1). MDA-MB-231 did not induce *IFNB1* expression (Supplementary Fig. 1C). The three cell lines also showed a differential response to single dose (8 Gy) and fractionated radiation (3×8 Gy); MDA-MB-468 responded best to 3×8 Gy, MDA-MB-436 to 8 Gy, while MDA-MB-231 showed a similar response to 8 Gy and 3×8 Gy (Supplementary Fig. 1).

### Cytosolic dsDNA negatively correlates with autophagy rates

Of the three TNBC cell lines, MDA-MB-468 showed the highest level of cytosolic dsDNA and the strongest induction of the IFN-I response after radiation and taxol (Fig. 1A, C and Supplementary Fig. 1). We hypothesized that the three TNBC cell lines have different rates of autophagy as a common cellular pathway implicated in the clearance of cytosolic dsDNA [26–28]. Autophagic flux was assessed by measuring p62 and LC3 II/I levels [35]. A decrease of p62 upon treatment can indicate increased autophagic flux while autophagy inhibition leads to accumulation of p62 [35]. Upon autophagy induction, cytosolic LC3 (LC3 I) is conjugated to phosphatidylethanolamine (PE) (LC3 II) and attached to the autophagosomal membrane, resulting in the higher ratio of LC3 II/I [25, 35]. Compared to MDA-MB-436 and MDA-MB-231, MDA-MB-468 cells showed relatively low levels of LC3 II/I indicating a lower basal rate of autophagy in those cells. While MDA-MB-468 showed higher levels of cytosolic dsDNA and lower levels of basal autophagy, MDA-MB-436 and MDA-MB-231 cells showed a relatively high autophagic rate and low levels of cytosolic dsDNA (Fig. 1C, D). Collectively, our data suggest that the level of cytosolic dsDNA negatively correlates with the rate of autophagy.

### Autophagy induction or inhibition modulate the levels of cytosolic dsDNA

To further investigate the role of autophagy in regulating dsDNA levels in MDA-MB-468, we measured the levels of cytosolic dsDNA upon induction of autophagy. For that we used a combination of EBSS starvation media and mammalian target of rapamycin (mTOR) inhibitor Torin-1, which is commonly used to induce bulk autophagy. Upon treatment with Torin-1/EBSS, cytosolic dsDNA was almost completely abolished in both control and irradiated cells (Fig. 1E, F). Conversely, inhibition of autophagy with SAR405 in MDA-MB-231 and MDA-MB-436 increased cytosolic dsDNA (Fig. 1G, H). SAR405 is a VPS34 inhibitor, which blocks the synthesis of phosphatidylinositol 3-phosphate (PI3P) [36]. In summary, autophagy modulates the levels of cytosolic dsDNA, whereby induction of autophagy decreases and inhibition of autophagy increases the levels of cytosolic dsDNA.

### Inhibition of autophagy modulates the immunogenic response in TNBC cell lines

Autophagy can degrade cytosolic dsDNA and could thereby dampen the immunogenic response, which may be particularly relevant for the two cell lines that show high autophagy rates, MDA-MB-436 and MDA-MB-231. To assess the effect of autophagy on the IFN-I response, cells were treated with Torin-1/EBSS and with different concentrations of the two autophagy inhibitors, SAR405 and bafilomycin A1, and analyzed by immunoblotting of the STING signaling cascade including pSTING, pTBK1 and pSTAT1 (Supplementary Fig. 2). Bafilomycin A1 was used as a late-stage autophagy inhibitor that blocks autophagosomal-lysosomal fusion [37]. In MDA-MB-468, which has low autophagy rates (Fig. 1D), STING signaling was largely unaffected by inhibition of autophagy (Supplementary Fig. 2A). In contrast, MDA-MB-436 and MDA-MB-231, which have high autophagy rates (Fig. 1D), showed a modest induction of the STING signaling cascade after treatment with both inhibitors (Supplementary Fig. 2B,C). In summary, inhibition of autophagy was found to modestly induce the STING signaling in cells with higher basal rates of autophagy.

### Combinatorial effects of radiation and autophagy inhibition on STING and NF-κB signaling in TNBC cell lines

To assess potential synergistic effects between radiation/taxol and autophagy inhibition, we treated TNBC cells with 8 Gy or 3×8 Gy and harvested on day 3 after the last fraction. Taxol treatment was applied for two days. In addition, we compared the effects of X-ray and C-ion irradiation, considering that C-ions were shown to have a higher immunogenic potential compared to photons [17]. C-ions were applied in a single dose of 8 Gy. The autophagy inhibitor SAR405 was used in combination with radiation or taxol, whereas bafilomycin A1 was only combined with taxol due to a severe loss in viability when combined with radiation. The activation of the STING and NF-κB signaling cascades was analyzed by immunoblotting or nuclear IRF3 staining by flow cytometry [38], while mRNA levels of *IFNB1*, *CXCL10*, *PD-L1* and *IL-6* were determined by RT-qPCR.

**Fig. 2:**
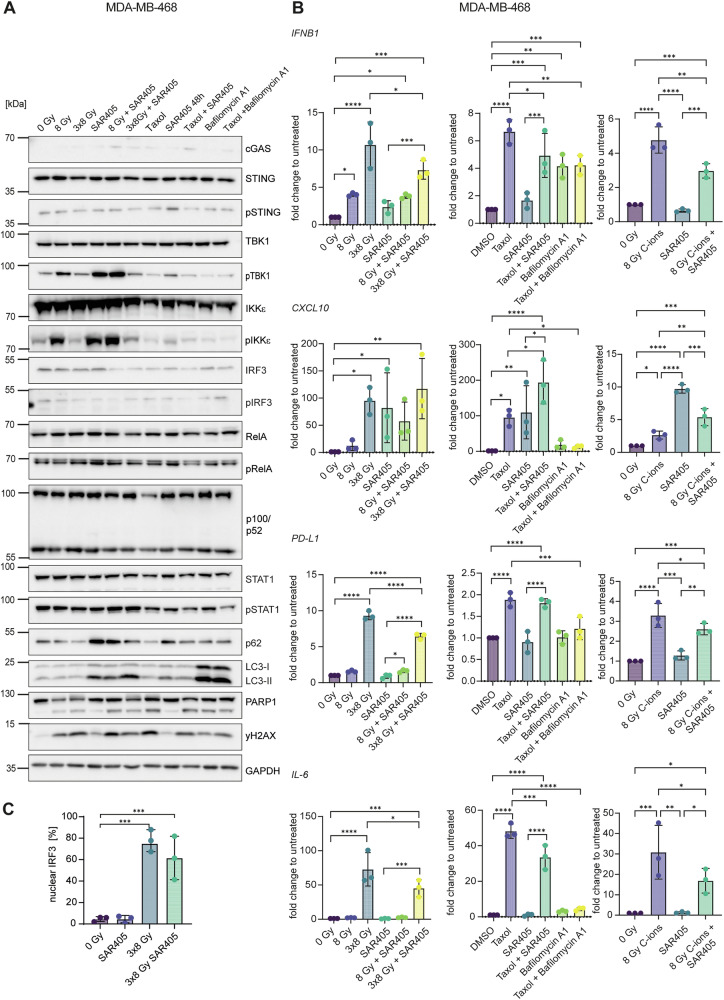
Hypofractionated X-ray irradiation induces the IFN-I response in MDA-MB-468. Cells were irradiated with 8 Gy or 3×8 Gy X-rays or 8 Gy C-ions and harvested 72 h after the last irradiation. Cells were treated for 48 h with 1 μM of taxol, 3 μM SAR405 or 200 nM bafilomycin A1. When combined with radiation, SAR405 and bafilomycin A1 were added 72 h before harvesting. **A** Western blots of MDA-MB-468 upon different treatments. GAPDH served as a loading control. **B** RT-qPCR analysis of *IFNB1*, *CXCL10*, *PD-L1* and *IL-6* mRNA levels in MDA-MB-468. *TBP* served as a housekeeping gene. **C** Nuclear IRF3 analysis by flow cytometry for MDA-MB-468 control cells and after treatment with SAR405, 3×8 Gy and 3×8 Gy combined with SAR405 (as described under **A**). Flow cytometry measurements were analyzed with FlowJo_v10.8.1. The bars and error bars represent mean values ± standard deviation. Three biologically independent experiments were performed. *P* values were calculated with a one-way ANOVA (* ≤ 0.05; **≤ 0.01; ***≤ 0.005, ****≤0.0001).

In MDA-MB-468, *IFNB1*, *CXCL10*, *PD-L1* and *IL-6* were upregulated after 3×8 Gy and taxol, while inhibition of autophagy with SAR405 induced *CXCL10* expression (Fig. 2B). The levels of pTBK1 and pIKKε were increased after SAR405 treatments (Fig. 2A). In combination with radiation, SAR405 led to decreased *IFNB1*, *PD-L1* and *IL-6* mRNA levels without affecting *CXCL10* (Fig. 2B). The induction of the STING-IRF3-IFNB1 pathway was confirmed by an increase in nuclear IRF3 levels after 3×8 Gy, which were slightly reduced in combination with SAR405 (Fig. 2C and Supplementary Fig. 3A). In combination with taxol, the late-stage autophagy inhibitor bafilomycin A1 severely dampened the effects observed after taxol alone (Fig. 2B). Combinatorial effects of C-ion irradiation and SAR405 were comparable to X-rays but differed in magnitude, with 8 Gy C-ions showing a lower induction of *CXCL10* and a higher induction of *IL-6* compared to 8 Gy X-rays (Fig. 2B).

**Fig. 3:**
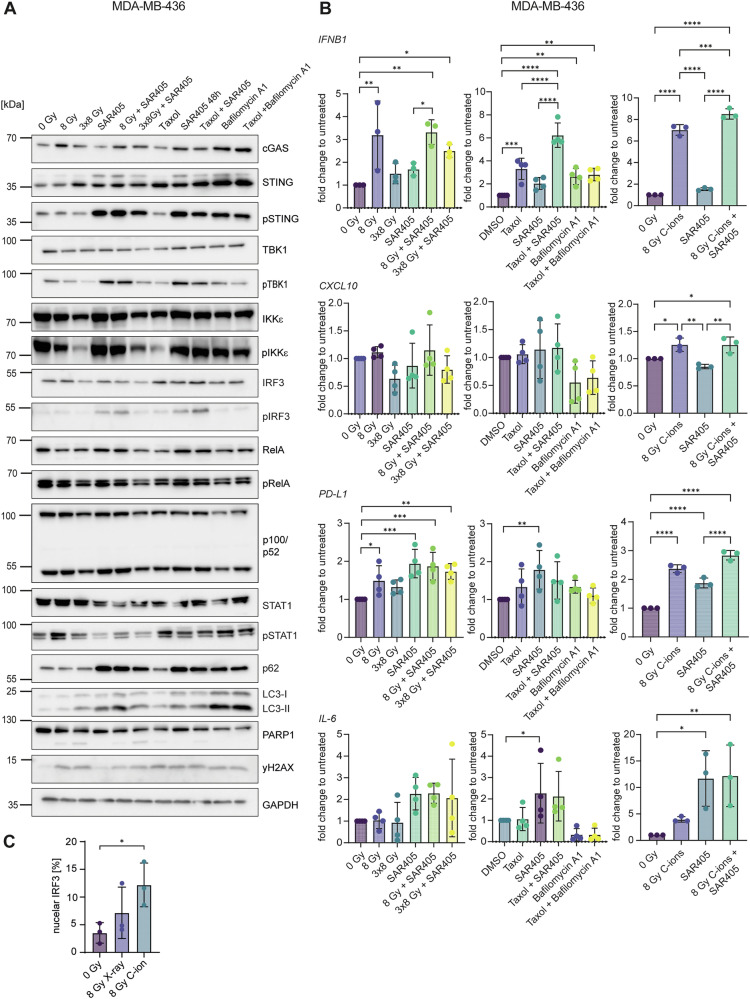
C-ion irradiation induces the IFN-I response in MDA-MB-436. Cells were irradiated with 8 Gy or 3×8 Gy X-rays or 8 Gy C-ions and harvested 72 h after the last irradiation. Cells were treated for 48 h with 1 μM of taxol, 3 μM SAR405 or 200 nM bafilomycin A1. When combined with radiation, SAR405 and bafilomycin A1 were added 72 h before harvesting. **A** Western blots of MDA-MB-436 upon different treatments. GAPDH served as a loading control. **B** RT-qPCR analysis of *IFNB1*, *CXCL10*, *PD-L1* and *IL-6* mRNA levels in MDA-MB-436. *TBP* served as a housekeeping gene. **C** Nuclear IRF3 analysis by flow cytometry for MDA-MB-436 control cells and after treatment with 8 Gy X-rays or C-ions. Flow cytometry measurements were analyzed with FlowJo_v10.8.1. The bars and error bars represent mean values ± standard deviation. Three biologically independent experiments were performed. *P* values were calculated with a one-way ANOVA (* ≤ 0.05; **≤ 0.01; ***≤ 0.005, ****≤0.0001).

In MDA-MB-436, pSTAT1 and *IFNB1* were upregulated after 8 Gy or taxol (Fig. 3). Autophagy inhibition with SAR405 induced pSTING, pTBK1, pIRF3, *IL-6* and *PD-L1*, whereas pSTAT1 was reduced (Fig. 3). A combination of SAR405 and 8 Gy radiation or taxol showed a similar effect as radiation or taxol alone (Fig. 3). Interestingly, 8 Gy C-ions were more potent compared to X-rays in inducing nuclear IRF3, *IFNB1* and pSTAT1 (Fig. 3B, C and Supplementary Figs. 3B and 4).

**Fig. 4:**
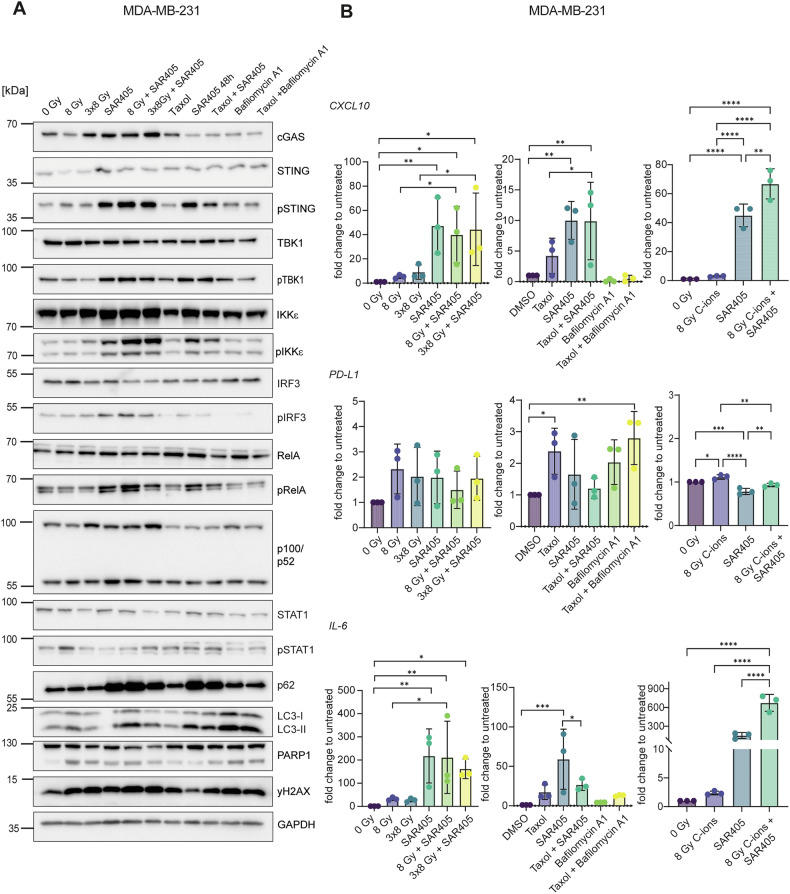
Autophagy inhibition induces NF-κB signaling in MDA-MB-231. Cells were irradiated with 8 Gy or 3×8 Gy X-rays or 8 Gy C-ions and harvested 72 h after the last irradiation. Cells were treated for 48 h with 1 μM of taxol, 3 μM SAR405 or 200 nM bafilomycin A1. When combined with radiation, SAR405 and bafilomycin A1 were added 72 h before harvesting. **A** Western blots of MDA-MB-231 upon different treatments. GAPDH served as a loading control. **B** RT-qPCR analysis of *IFNB1*, *CXCL10*, *PD-L1* and *IL-6* mRNA levels in MDA-MB-231. *TBP* served as a housekeeping gene. The bars and error bars represent mean values ± standard deviation. Three biologically independent experiments were performed. P-values were calculated with a one-way ANOVA (* ≤ 0.05; **≤ 0.01; ***≤ 0.005, ****≤0.0001).

The strongest effect upon autophagy inhibition with SAR405 was observed in MDA-MB-231, which showed increased cGAS, pSTING, pTBK1 and pIRF3 levels, as well as pRelA (p65 subunit of NF-κB) (Fig. 4A). Accordingly, SAR405 significantly increased *CXCL10* and *IL-6* mRNA levels (Fig. 4B). Despite increased pIRF3, nuclear IRF3 was not detected by flow cytometry (Supplementary Fig. 3C), suggesting that only the STING-NF-κB pathway is active in these cells. *IFNB1* expression could not be detected, which may be due to activation of non-canonical NF-κB signaling as judged by an increase in p100/p52, which was previously shown to negatively regulate *IFNB1* expression [15, 16]. Given the absence of *IFNB1* and pSTAT1 induction, we conclude that STING signaling results in NF-κB-IL-6 induction in this cell line. Unlike SAR405, bafilomycin A1 did not show immunostimulatory effects (Fig. 4). Combinational treatments with SAR405 and X-ray irradiation or taxol showed a similar effect as SAR405 alone (Fig. 4). In this cell line, C-ion irradiation in combination with SAR405 showed a stronger induction of *CXCL10* and *IL-6* compared to X-rays (Fig. 4B).

In all three cell lines, C-ions induced more DNA damage compared to X-rays based on the quantitative assessment of γH2AX foci (Supplementary Fig. 5) and a stronger activation of the ATR-CHK1 pathway as judged by the higher levels of pATR, pCHK1 and pRPA S33 (Supplementary Fig. 4). Both radiation modalities induced apoptosis based on the cleavage of caspase-8, caspase-3 and PARP1. Necroptosis was induced more strongly by C-ions in MDA-MB-468 and MDA-MB-436 based on the increase in MLKL levels, while ferroptosis was induced more strongly by C-ions in MDA-MB-231 based on the increase in COX-2 (Supplementary Fig. 4). Collectively, these results do not explain why C-ions showed a stronger effect on the induction of the IFN-I pathway in the *BRCA*-mutated MDA-MB-436 cell line.

Taken together, we did not observe synergistic effects between radiation and autophagy inhibition in TNBC cell lines. Radiation predominantly induces *IFNB1* expression, whereas SAR405 induces the expression of the chemokine *CXCL10* via the NF-κB pathway. Hypofractionated (3×8 Gy) or single dose radiation (8 Gy) were most immunostimulatory in MDA-MB-468 and MDA-MB-436 respectively, while SAR405 showed the strongest effect in MDA-MB-231.

### Variable expression of cGAS/STING and oncogenic mutations in PTEN, BRCA1 and KRAS mutations may contribute to the differential response of TNBC cell lines to radiation and autophagy inhibitors

High autophagy rates in MDA-MB-231 may explain why autophagy inhibition in this cell line results in increased cytosolic dsDNA (Fig. 1G, H) and induction of cGAS/pSTING/pTBK1 signaling (Fig. 4). Conversely, low autophagy rates in MDA-MB-468 may explain why this cell line is non-responsive to autophagy inhibitors (Fig. 2).

Furthermore, we observed pronounced differences in cGAS and STING protein levels among the three cell lines, which may contribute to their differential response to radiation or taxol. In MDA-MB-468, there was no detectable cGAS but STING levels were markedly higher compared to the other two cell lines (Supplementary Fig. 6A). In contrast, MDA-MB-436 and MDA-MB-231 expressed moderate amounts of cGAS and STING. High STING levels in MDA-MB-468 were previously linked with the PTEN mutation [39]. PTEN dephosphorylates RAB7, which causes RAB7 mislocalization and prevents the trafficking of STING-containing vesicles to the lysosome. In PTEN-mutated cells, RAB7 is hyperphosphorylated by TBK1/IKKε and cannot induce lysosome-mediated degradation of STING, resulting in stronger STING-dependent immune signaling [39]. The absence of cGAS in MDA-MB-468 is corroborated by the publicly available mRNA expression data [40] and may be compensated by other dsDNA sensors such as RNA polymerase III, DNA-PK or DDX41 [40–43] (Supplementary Fig. 6B).

*BRCA1* mutation impairs DNA repair by homologous recombination and may thus sensitize MDA-MB-436 to C-ions (Fig. 3). C-ions induce clustered DNA damage, which is harder to repair compared to photon-induced DNA damage and was shown to sensitize DNA repair-deficient cancers [44–46]. We found that C-ions induce more DNA damage compared to X-rays and elicit a stronger activation of the ATR-CHK1 pathway (Supplementary Figs. 4 and 5) in all three TNBC cell lines, which can therefore not explain the strongest effect of C-ions on the IFN-I response in the *BRCA*-mutated MDA-MB-436 cell line.

The oncogenic KRAS G13D mutation in MDA-MB-231 may explain the suppression of the IFN-I response and the activation of NF-κB signaling and *IL-6* expression, which have been already reported for KRAS-mutated cancers [23, 24, 47]. Activation of STING signaling due to autophagy inhibition in this cell line was found to potentiate STING-dependent NF-κB signaling.

Overall, differential response of TNBC cell lines to radiation and autophagy inhibitors can be at least partly attributed to their genetic background.

### Autophagy inhibition with the VPS34 inhibitor SAR405 sequesters phosphorylated TBK1 in p62 aggregates

TNBC cells treated with SAR405 uniformly showed an increase in pTBK1 levels (Figs. 2–4, Supplementary Fig. 6A). TBK1 is not only involved in STING signaling but is also required for bulk and selective autophagy and is particularly important during early phases of autophagy by enabling the recruitment of the ULK1 complex [48, 49]. Recently it was shown that TBK1 can functionally take over the role of ULK1 to directly interact with the PI3K complex I and initiate OPTN-driven mitophagy [50]. Furthermore, TBK1 interacts with and phosphorylates the selective autophagy receptor p62, which enhances its binding to polyubiquitinated mitochondria and their clearance by mitophagy [51]. TBK1 also phosphorylates p62 within TAX1BP1-positive ubiquitinated aggregates to ensure efficient phase separation, engulfment and clearance of the cargo [52]. Loss of the ULK1 complex member FIP200 or autophagy inhibition promote the formation of TBK1-p62 aggregates in which TBK1 is highly phosphorylated and hyperphosphorylates p62, rendering these aggregates insoluble [52].

**Fig. 5:**
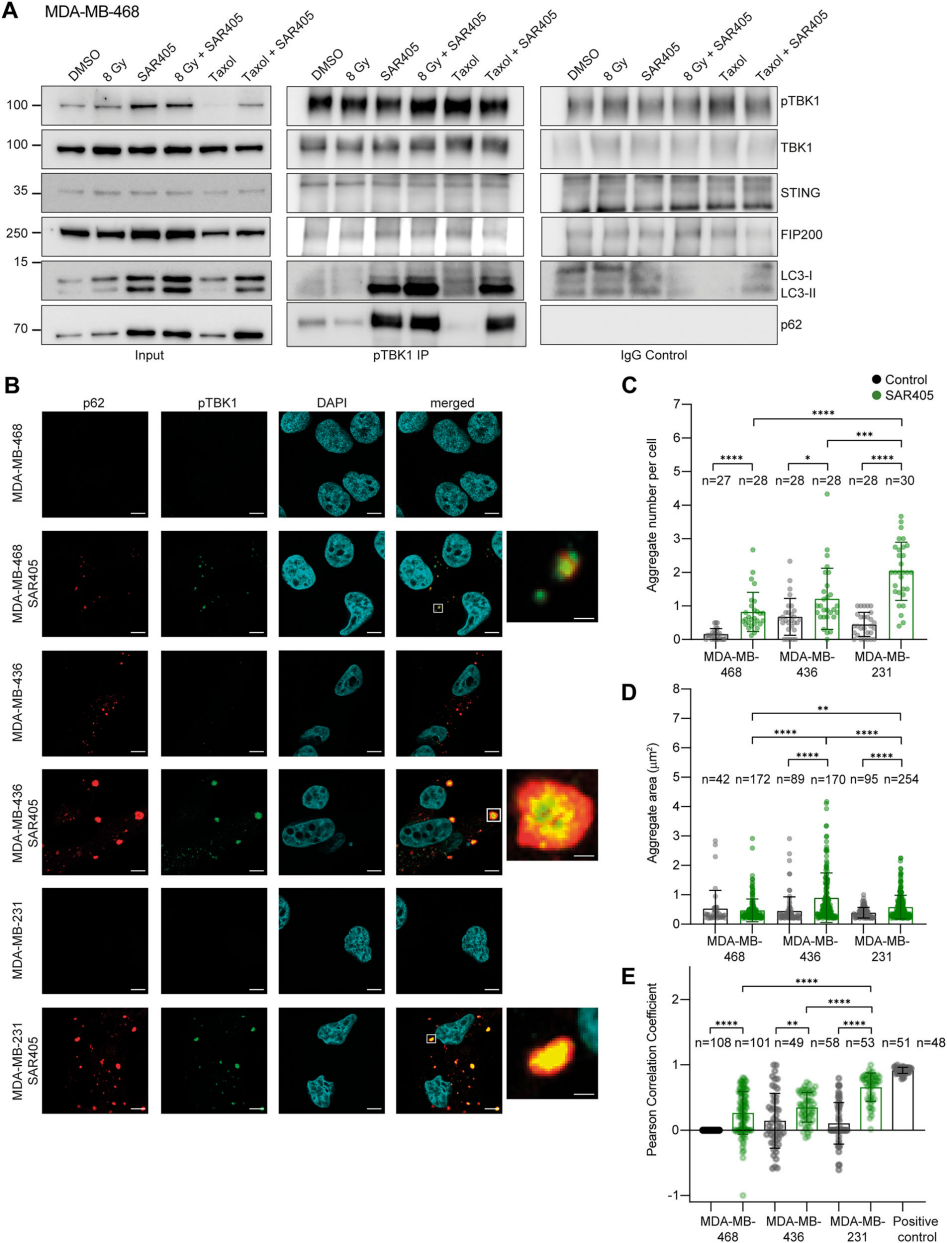
Autophagy inhibition drives pTBK1-p62 aggregate formation in TNBC cell lines. **A** Immunoprecipitation of pTBK1 or IgG control in MDA-MB-468 cells followed by Western blotting. Cells were irradiated with 8 Gy X-rays and harvested 72 h after the last irradiation. Cells were treated for 48 h with 1 μM of taxol or 3 μM SAR405. When combined with radiation, SAR405 was added 48 h before harvesting. **B** Representative immunofluorescence images of MDA-MB-468, MDA-MB-436 and MDA-MB-231 control cells and cells treated with 3 μM SAR405 for 48 h. Cells were stained with anti-pTBK1 antibody (green), anti-p62 antibody (red) and the nucleus was stained with DAPI (blue). Scale bar: 10 μm. Zoom-in of representative aggregates from SAR405-treated cells are shown on the right. Scale bar: 1 μm. **C** Quantification of mean cytoplasmic aggregate number per cell per image for (**B**). **D** Quantification of aggregate area in cells from (**B**). **E** Quantification of pTBK1-p62 colocalization based on the Pearson correlation coefficient in cells from (**B**). The bars and error bars represent mean values ± standard deviation. Two biologically independent experiments were performed. P-values were calculated with Welch’s t-test (*≤ 0.05; **≤ 0.01; ***≤ 0.005, ****≤0.0001).

Thus we hypothesized that autophagy inhibition with SAR405 may sequester TBK1 in pTBK1-p62 aggregates and thereby impair STING signaling. To test this, we performed a co-immunoprecipitation analysis of pTBK1 in MDA-MB-468 treated with SAR405, 8 Gy ± SAR405 and taxol ± SAR405 (Fig. 5A). Samples treated with SAR405 alone or in combination with radiation or taxol showed a highly increased interaction of pTBK1 with p62 and LC3 (Fig. 5A). Immunofluorescence analysis revealed SAR405-induced pTBK1 aggregates in all three TNBC cell lines that also contain p62 (Fig. 5B). MDA-MB-231 showed the strongest increase in pTBK1-p62 aggregate number upon SAR405 treatment and the highest colocalization coefficient between pTBK1 and p62 within the aggregates (Fig. 5C–E). Interestingly, pTBK1 and p62 are differently organized within the aggregates; in MDA-MB-468 both proteins are intermixed, whereas in MDA-MB-231 and MDA-MB-436 pTBK1 is encapsulated by p62 (Fig. 5B, right). These differences in aggregate structure may reflect p62 concentration differences, whereby a higher amount of p62 in MDA-MB-231 and MDA-MB-436 aggregates (Supplementary Fig. 6A) may result in the formation of a p62 cage around the pTBK1-p62 aggregate (Fig. 5B). Collectively, these results suggest that sequestration of pTBK1 in p62 condensates may be a general mechanism of autophagy inhibition with SAR405, favoring TBK1-independent NF-κB signaling over IFN-I signaling.

### The immunogenic response induced by radiation and autophagy inhibition with SAR405 is dependent on STING and/or NF-κB

**Fig. 6:**
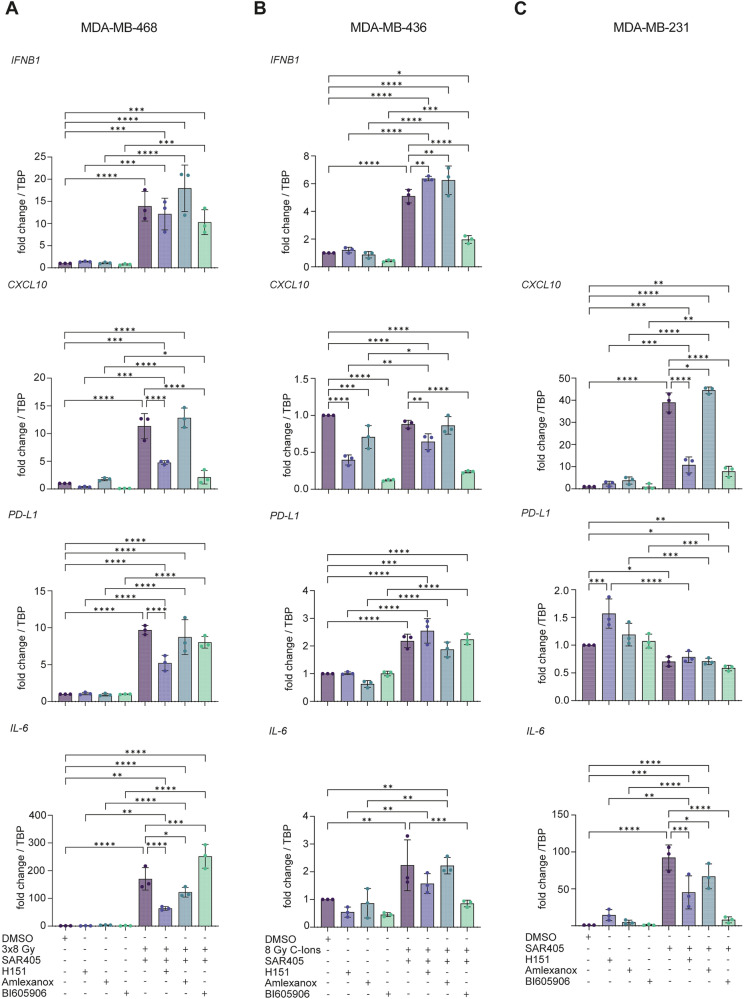
STING and NF-κB-dependent immunogenic response in TNBC cell lines treated with radiation and autophagy inhibitor. Cells were treated with the STING inhibitor H151 (10 μM), the TBK1/IKKε inhibitor amlexanox (20 μM) or the NF-κB inhibitor BI605906 (10 μM) alone or in combination with **A** 3×8 Gy X-rays in MDA-MB-468, **B** 8 Gy C-ions and 3 μM SAR405 in MDA-MB-436 and **C** 3 μM SAR405 in MDA-MB-231. H151, amlexanox and BI605906 were applied directly after the last irradiation/SAR405 treatment and kept for 72 hours. All cells were harvested after 72 hours of treatment. RT-qPCR analysis of *IFNB1*, *CXCL10*, *PD-L1* and *IL-6* mRNA levels is shown. *TBP* served as a housekeeping gene. The bars and error bars represent mean values ± standard deviation. Three biologically independent experiments were performed. P-values were calculated with a one-way ANOVA (* ≤ 0.05; **≤ 0.01; ***≤ 0.005, ****≤0.0001).

To further investigate the regulation of cytokine expression in different TNBC cell lines, we applied three different inhibitors: STING inhibitor (H151 [53]), TBK1/IKKε inhibitor (amlexanox [54]) and NF-κB inhibitor (BI605906 [55]). Amlexanox was shown to inhibit TBK1/IKKε with IC_50_ = 1–2 μM [54]. BI605906 inhibits IKKβ and thereby prevents activation of the RelA (p65) subunit of NF-κB [55]. We combined these inhibitors with treatment conditions that showed the strongest induction of cytokine expression in the three TNBC cell lines: 3×8 Gy X-rays in MDA-MB-468, 8 Gy C-ions+SAR405 in MDA-MB-436 and SAR405 in MDA-MB-231 (Fig. 6).

In MDA-MB-468 treated with 3×8 Gy X-rays, STING, TBK1 and NF-κB inhibitors showed no significant effect on *IFNB1* expression (Fig. 6A). The STING inhibitor reduced *PD-L1*, *CXCL10* and *IL-6* expression, the NF-κB inhibitor reduced *CXCL10* expression, while the TBK1 inhibitor modestly reduced *IL-6* levels (Fig. 6A).

In MDA-MB-436 treated with 8 Gy C-ions and SAR405, the NF-κB inhibitor significantly reduced the expression of *IFNB1*, *CXCL10* and *IL-6*, whereas the STING inhibitor and the TBK1 inhibitor had a weak or no effect (Fig. 6B). We therefore conclude that the induction of immunogenic response in MDA-MB-436 after radiation and autophagy inhibition relies mainly on TBK1-independent NF-κB signaling.

In MDA-MB-231, NF-κB and STING inhibitors abrogated SAR405-induced expression of *CXCL10* (Fig. 6C). The NF-κB inhibitor also abrogated the induction of *IL-6*, while the STING inhibitor reduced it (Fig. 6C). The TBK1 inhibitor had no effect on SAR405-induced expression of *CXCL10* (Fig. 6C), suggesting that NF-κB signaling in MDA-MB-231 is independent of TBK1. Taken together, our results show that in MDA-MB-231 SAR405 induces cytokine expression through STING-dependent but TBK1-independent NF-κB signaling.

### TNBC cells treated with the autophagy inhibitor SAR405 or radiation activate cells of the innate immune system

**Fig. 7:**
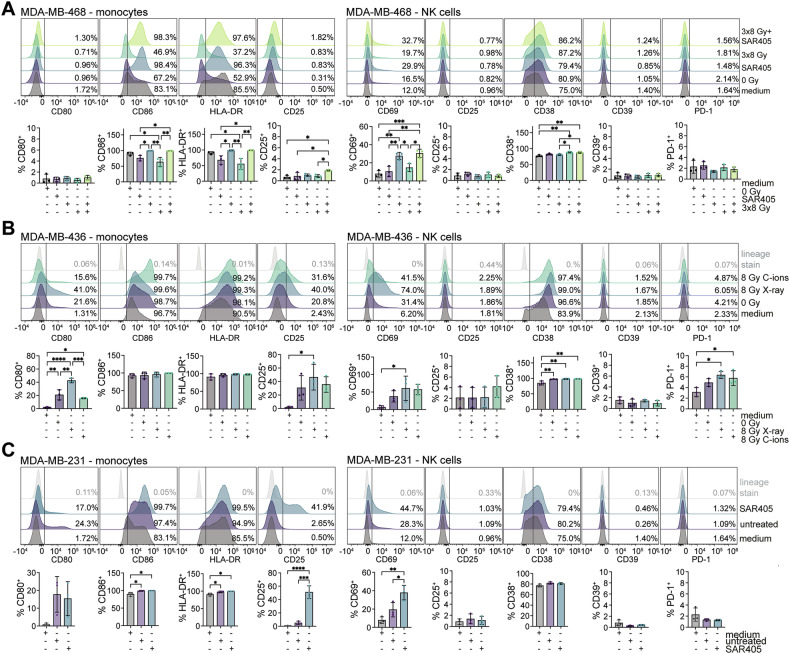
Response of primary human monocytes and NK cells to supernatants from TNBC cells treated with the autophagy inhibitor SAR405 or radiation. PBMCs from three different donors were incubated for 20 h in the presence of the culture medium (control) or cell-free supernatants from **A** MDA-MB-468, **B** MDA-MB-436 and **C** MDA-MB-231 cells. **A** MDA-MB-468 cells were treated with 3 μM SAR405 for 96 h, 3×8 Gy X-rays or a combination thereof. Supernatants were harvested 96 h after the last irradiation. When combined with radiation, SAR405 was added 96 h before harvesting. **B** MDA-MB-436 cells were irradiated with 8 Gy X-rays or C-ions and harvested after 96 h. **C** MDA-MB-231 cells were treated with 3 μM SAR405 for 96 h. Human monocytes (left) and NK cells (right) were identified according to lineage markers and their activation was assessed based on the analysis of activation markers CD80, CD86, HLA-DR, CD25 (monocytes), CD69, CD25, CD38 (NK cells) or activation-induced molecules CD39 and PD-1 with immunoregulatory functions (NK cells) by spectral flow cytometry. Representative histograms of one donor (top) and percentages of marker-positive cells ± standard deviation of three PBMC donors (bottom) are shown. In histograms, the gating for the positive cells is based on the lineage stain (depicted in gray) and is indicated by a vertical line. Experiments in A and C were performed at the same time, thus they share the medium control. Statistical significance was assessed using one-way ANOVA with Tukey’s *post-hoc* test (*≤ 0.05; **≤ 0.01; ***≤ 0.005, ****≤0.0001).

To further probe the immunostimulatory effects of type I interferon and cytokines generated by the TNBC cells, we incubated freshly isolated peripheral blood mononuclear cells (PBMCs) with the cell-free supernatants from treated TNBC cells for 20 h, followed by the PBMC analysis using spectral flow cytometry (Fig. 7 and Supplementary Figs. 7–9). Lineage markers (Supplementary Table 2) allowed us to differentiate between monocytes, myeloid dendritic cells (mDCs), plasmacytoid dendritic cells (pDCs), natural killer (NK) cells, natural killer T (NKT) cells, CD4 and CD8 T cells, and B cells (Supplementary Fig. 7), and additional well-known activation-induced surface markers helped us scrutinize their activation state [56, 57]. For monocytes and DCs, we assessed co-stimulatory ligands CD80 and CD86, the antigen-presenting molecule HLA-DR and CD25, which are all involved in antigen presentation and T cell activation [56, 57]. Activation of lymphocytes, with the focus on subsets (CD8 T cells, NK cells and NKT cells) that are crucial in anti-tumor immune responses, was scored based on early-to-intermediate activation markers CD69, CD25, CD38 [55]. Here, we also evaluated the expression of immunoregulatory molecules PD-1 and CD39, which are known to be induced upon immune cell activation [55]. To assess chemokine binding to immune cells and their potential chemotactic capacity, we analyzed surface expression of CCR5 (a receptor for proinflammatory chemokines CCL3, CCL4, CCL5 that we previously found to be highly expressed by activated myeloid cells [58]) and CXCR3 (a receptor for IFN-inducible chemokines CXCL9, CXCL10 and CXCL11) [59, 60]. CCR5 and CXCR3 are preferentially expressed in myeloid and lymphoid cells respectively, and play a pivotal role in immune cell recruitment to the sites of inflammation, including tumors [59, 60]. Upon chemokine binding, these receptors are cleared from the cell surface by the process of ligand-induced internalization [61, 62]. We compared conditions that showed the strongest induction of cytokine expression in the three TNBC cell lines: 3×8 Gy X-rays ± SAR405 in MDA-MB-468, 8 Gy X-rays vs 8 Gy C-ions in MDA-MB-436 and SAR405 in MDA-MB-231.

Supernatants from the non-treated MDA-MB-468 cells downregulated otherwise strongly expressed CD86 and HLA-DR on monocytes compared to the control (medium only), and had no effect on lowly expressed CD80 and CD25 (Fig. 7A) or other markers in the monitored PBMC subsets (Fig. 7A, Supplementary Figs. 8A and 9A). Interestingly, we observed a strong upregulation of CD86 and HLA-DR on monocytes as well as CD69 on NK cells after SAR405 alone or in combination with radiation, indicating their activation (Fig. 7A). SAR405-treated cells also showed a reduced surface expression of the chemokine receptor CCR5 on monocytes and NK cells, and CXCR3 on CD8 T cells, suggesting that they were stimulated by chemokine binding (Supplementary Fig. 10A). Radiation in combination with SAR405 induced CD86 in pDCs (Supplementary Fig. 8A). NKT cells, which are considered as a functional bridge between innate and adaptive immunity, did not significantly respond to any of the treatments within 20 h (Supplementary Fig. 9A).

In experiments with MDA-MB-436 supernatants, both 8 Gy X-rays and 8 Gy C-ions showed a stronger immunostimulatory effect in a cell type- and marker-specific pattern than mildly activating supernatants from the non-irradiated MDA-MB-436 cells. On monocytes, X-rays—but not C-ions—upregulated CD80 and CD25 the most (Fig. 7B). NK and NKT cells were also activated by X-rays, leading to a significant upregulation of CD69 and PD-1 (in NK cells) and CD38 (in NKT cells) (Fig. 7B and Supplementary Fig. 9B). CD8 T cells also showed a tendency towards activation, with a slight increase in the surface expression of CD69 and CD38 after 8 Gy X-rays, or CD25 and PD-1 after 8 Gy C-ions (Supplementary Fig. 9B).

Finally, supernatants from untreated MDA-MB-231 cells also showed immunostimulatory effects by increasing multiple activation markers on monocytes (Fig. 7C) and DCs (Supplementary Fig. 8C). CD69, an activation marker with the fastest kinetics, was also weakly increased on NK, NKT and CD8 T cells (Fig. 7C, Supplementary Fig. 9C). Importantly, SAR405 strongly upregulated CD25 on monocytes and mDCs, as well as the expression of all monitored markers on pDCs (Fig. 7C and Supplementary Fig. 8C). CD69 was also substantially increased on NK cells (and non-significantly also on NKT and CD8 T cells) upon incubation with the SAR405-pretreated MDA-MB-231 supernatants (Fig. 7C and Supplementary Fig. 9C). CCR5 was reduced on monocytes, while CXCR3 was reduced on pDCs, mDCs, NK, NKT and CD8 T cells, indicating their engagement by the respective chemokines (Supplementary Fig. 10).

Collectively, these results reveal that the autophagy inhibitor SAR405 and radiation applied onto TNBC cells can activate cells of the innate immune system, and have the potential to attract both innate and adaptive immune cells via chemokines, which are prerequisite steps towards immune system-mediated tumor elimination.

## Discussion

**Fig. 8:**
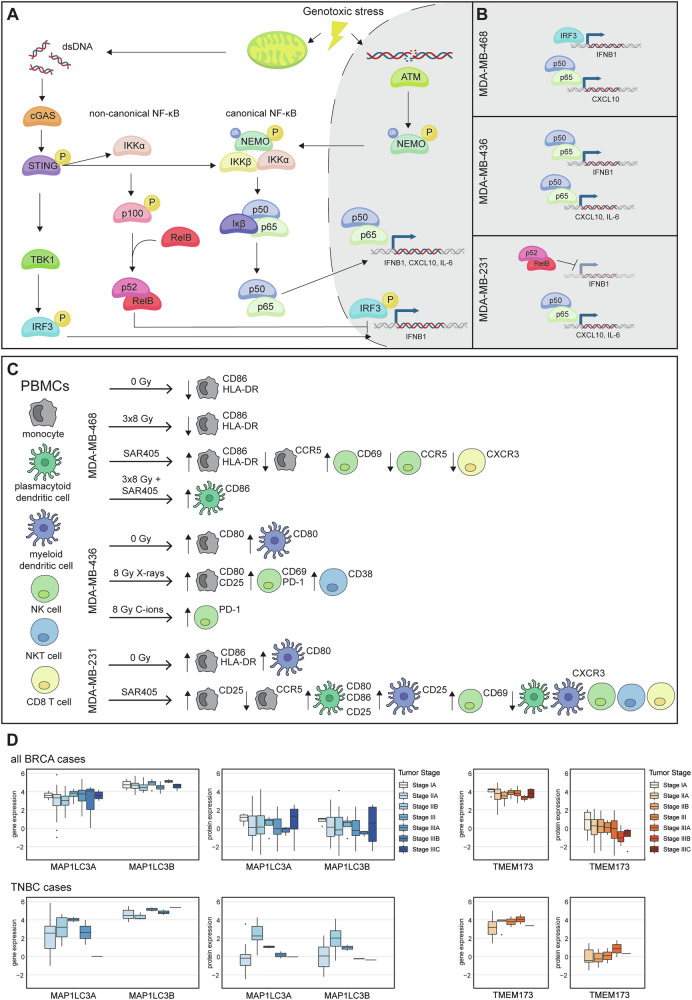
Immunogenic signaling and immune cell activation after irradiation or autophagy inhibition of TNBC cell lines. **A** Mitochondrial damage leads to the release of dsDNA into the cytoplasm, which activates the cGAS-STING signaling pathway. cGAS-STING signaling culminates in the translocation of transcription factors IRF3 and NF-κB into the nucleus, which can induce the expression of *IFNB1* and proinflammatory chemokines and cytokines such as *CXCL10* and *IL-6*. Canonical NF-κB signaling can also be induced by the ATM kinase, which is activated by double-strand DNA breaks (DSBs) induced by genotoxic stress. Non-canonical NF-κB can dampen the expression of *IFNB1*. **B** In MDA-MB-468, the induction of *CXCL10* is dependent on NF-κB, while *IFNB1* is mainly driven by IRF3. In MDA-MB-436, signaling is dominated by the canonical NF-κB pathway with p50-p65 driving transcription of *IFNB1*, *CXCL10* and *IL-6*. In MDA-MB-231, proinflammatory signaling is induced by STING-dependent canonical NF-κB signaling. Non-canonical NF-κB signaling likely dampens the transcription of *IFNB1*. **C** Supernatants from MDA-MB-468 cells, that are otherwise immunosuppressive, can activate monocytes, NK cells and CD8 T cells after autophagy inhibition with SAR405. Supernatants from X-ray-irradiated MDA-MB-436 activate monocytes, NK and NKT cells. Supernatants from MDA-MB-231 treated with SAR405 can activate monocytes, DCs, NK, NKT and CD8 T cells. **D** Gene and protein expression data for LC3 (blue) and STING/TMEM173 (orange) was accessed from previously published, publicly available datasets [77] and shown per tumor stage. Patient metadata was used to differentiate between all BRCA cases (top) and only TNBC cases (bottom).

The regulation of immune signaling and inflammatory response is of profound importance for tumor microenvironment and cancer cell fate. Understanding the genetic make-up of cancer cells and regulatory mechanisms that rewire these pathways is therefore key for predicting patients’ response to available treatments and devising personalized treatment strategies. Our study reveals differential effects of radiation and autophagy inhibition in three human TNBC cell lines with regard to STING and NF-κB signaling, the expression of *IFNB1* and inflammatory chemokines such as *CXCL10*, and the activation of innate immune cells (Fig. 8A–C). We conclude that: i) radiation induces the IFN-I pathway; ii) autophagy inhibition induces NF-κB and *CXCL10*; iii) autophagy inhibition with the VPS34 inhibitor SAR405 causes sequestration of pTBK1, which may promote the NF-κB pathway; iv) autophagy inhibition has a stronger effect on the activation of innate immune cells compared to radiation. The cell line with the lowest autophagy rates and highest levels of cytosolic dsDNA showed the strongest induction of *IFNB1* after radiation, while the cell line with the highest autophagy rates showed the strongest immunostimulatory effects of autophagy inhibition via NF-κB signaling.

It was previously shown that irradiated mouse TNBC TS/A cells accumulate dsDNA of mitochondrial origin after radiation [26]. Accordingly, irradiated human TNBC cells also showed radiation-induced accumulation of cytosolic dsDNA, largely of mitochondrial origin based on colocalization with the mitochondrial marker TFAM (Fig. 1), which was recently shown to act as an autophagy receptor for cytoplasmic mitochondrial DNA [27]. We did not detect micronuclei in irradiated TNBC cells and thus can rule them out as a source of cytosolic dsDNA (data not shown). Alternatively, R-loop derived cytosolic RNA-DNA hybrids were recently proposed to induce a cGAS-STING-driven immune response [63]. Furthermore, dying cells release DNA into the intercellular space from where it can be taken up by neighboring cells, which would also result in the appearance of cytosolic DNA [64].

Human TNBC cell lines showed variable levels of radiation-induced cytosolic dsDNA, which correlate with their autophagy rates. MDA-MB-468 cells have significantly more dsDNA in the cytoplasm compared to MDA-MB-436 and MDA-MB-231, owing to the lowest basal rate of autophagy as indicated by low LC3 II/I ratio (Fig. 1), which is also supported by the literature [65]. Induction of autophagy significantly diminished cytosolic dsDNA in MDA-MB-468. In contrast, MDA-MB-436 and MDA-MB-231 exhibit higher autophagic rates, thus enabling efficient clearance of cytosolic dsDNA, which can be rescued by autophagy inhibition. Accordingly, of the three cell lines MDA-MB-468 showed the strongest induction of *IFNB1* in response to radiation or taxol (Fig. 2). However, this did not translate into the activation of innate immune cells, which may be due to the profound immunosuppressive capacity of this cell line at the baseline (Fig. 7A) and immunosuppressive mediators that are transiently induced by radiation such as PD-L1 [66]. PD-L1 can be released from irradiated cancer cells via exosomes and bind to the PD-1 receptor on NK cells, thereby preventing their activation [67, 68].

MDA-MB-436 and MDA-MB-231 were less responsive to radiation and taxol, which may be due to higher rates of autophagy and a limited pool of native STING observed in these cell lines. In the *BRCA*-mutated TNBC cell line MDA-MB-436, particle radiation with C-ions showed the strongest *IFNB1* and *PD-L1* expression (Fig. 3), which relies on STING-independent NF-κB signaling (Fig. 6B). By preventing DNA damage repair, the PARP inhibitor olaparib was recently shown to sensitize *BRCA*-mutated TNBC cells to C-ion irradiation [46]. In general, *BRCA*-mutated cancers are characterized by genomic instability due to deficient DNA repair by homologous recombination, which promotes the generation of neoantigens, robust cGAS-STING signaling and increased number of tumor infiltrating lymphocytes, making it an attractive target for ICB [69, 70]. Although C-ions induced more DNA damage and *IFNB1*, X-rays exerted stronger immunostimulatory effects on innate immune cells (monocytes, NK, NKT cells) (Fig. 7B). This may be due to a higher upregulation of *PD-L1* after C-ion irradiation (Fig. 3), supporting the combination of C-ions with ICB in *BRCA*-mutated TNBC.

In contrast to the other two cell lines, MDA-MB-231 showed a strong induction of STING signaling through NF-κB upon inhibition of autophagy with SAR405 (Figs. 4, 6C). TBK1 is dispensable for STING-NF-κB activation, as previously shown in macrophages [10]. Of the three cell lines, MDA-MB-231 has the highest rate of autophagy and autophagy inhibition prevents effective clearance of cytosolic dsDNA leading to a robust induction of the STING signaling cascade (pSTING, pTBK1, pIRF3) (Fig. 4). However, this did not translate into *IFNB1* expression, which may be due to activation of non-canonical NF-κB signaling that was shown to impair *IFNB1* expression [15, 16]. Oncogenic KRAS G13D mutation may also contribute to the suppression of the IFN-I response [47]. Instead, SAR405 induced the expression of the chemokine *CXCL10* and the proinflammatory cytokine *IL-6* through the STING-NF-κB pathway (Fig. 6C). Activation of the IL-6-STAT3 axis was previously shown to induce the expression of chemokines such as CXCL10, which promote T cell infiltration [71]. In the PBMC activation assay, SAR405 strongly activated monocytes (CD25), NK cells (CD69) and pDCs (CD80, CD86, CD25) (Fig. 7C). SAR405-treated MDA-MB-468 cells, which also induce CXCL10 expression, showed a weaker but significant effect on the activation of monocytes (CD86, HLA-DR), NK cells (CD69) and pDCs (CD86) (Fig. 7A). The importance of CXCL10 for the immunostimulatory effects of SAR405 is corroborated by downregulation of the CXCL10 receptor CXCR3 on CD8 T cells (Supplementary Fig. 10A, C). However, STING-dependent activation of NF-κB RelB-IL-6-STAT3 was shown to promote tumor cell proliferation by blocking STAT1-induced apoptosis [13, 72]. A mouse model would be required to further distinguish between tumor-suppressive paracrine (leading to activation of immune cells) and tumor-promoting autocrine effects of SAR405-induced STING-NF-κB signaling.

In all three TNBC cell lines, SAR405 induces pTBK1-p62 aggregate formation (Fig. 5), which was previously observed in FIP200 knock-out (KO) mouse embryonic fibroblasts (MEFs) or upon treatment with bafilomycin A1 [52]. FIP200 KO cells showed impaired *IFNB1* production after poly I:C transfection [52]. SAR405-induced pTBK1-p62 aggregates trap highly active pTBK1, preventing it from activating the IFN-I pathway and shifting the signaling towards the NF-κB pathway. Induction of NF-κB signaling in relation to p62 condensates was previously observed for the PARP12 zinc finger-deficient mutant that relocalizes from stress granules to p62 condensates [73], suggesting that p62 condensates favor NF-κB pathway activation. The exact mechanism of how SAR405 activates the NF-κB pathway requires further analysis. Our data reveal that SAR405 can activate innate immune cells by promoting NF-κB-driven expression of chemokines such as *CXCL10*, without inducing *PD-L1*. In contrast, radiation-induced IFN-I signaling fails to activate immune cells due to the concomitant upregulation of *PD-L1*.

Higher rates of autophagy have been associated with poor prognosis in a variety of cancers including TNBC [74–76]. Interestingly, TNBC patients with high-grade tumors express higher levels of LC3 and STING compared to low-grade tumors and non-TNBC patients [77] (Fig. 8D), suggesting that autophagy inhibitors would be beneficial in these patients and may show synergistic effects with radiotherapy coupled with ICB to mitigate radiation-induced expression of PD-L1. In fact, autophagy inhibition in TNBC with high autophagy rates has recently shown promising results in improving chemotherapy efficacy [65].

Currently the only two FDA-approved autophagy inhibitor drugs are chloroquine and hydroxychloroquine, which are unspecific with many off-target effects [78]. An effort to produce highly specific small molecule inhibitors that might be better suited as targeted treatments in patients is under way [79]. Understanding the direct targets of autophagy inhibitors and their effects on tumor cells is crucial for achieving a desired anti-tumor response. When comparing two autophagy inhibitors (SAR405 and bafilomycin A1), we observed different effects on cGAS-STING signaling alone or in combination with radiation or taxol. Taxol was previously shown to activate cGAS-STING signaling in TNBC [30]. Combination with autophagy inhibitors was expected to potentiate this effect by preventing clearance of cytosolic dsDNA. However, no synergistic effect was observed when taxol was combined with either SAR405 or bafilomycin A1. On the contrary, bafilomycin A1 dampened the production of IFNB1 and ISGs. Besides targeting autophagy, bafilomycin A1 can also induce apoptosis [80]. Activation of the apoptotic caspase cascade can suppress mtDNA-induced type I IFN production [81]. Therefore, bafilomycin A1, by activating apoptosis, might suppress cGAS-STING signaling, which may be particularly pronounced after a prolonged treatment (48 h with taxol or 72 h with radiation; Figs. 2–4).

Taken together, our findings highlight the NF-κB-driven immunostimulatory effects of autophagy inhibition with the VPS34 inhibitor SAR405. They also suggest that NF-κB signaling and autophagy rates could serve as predictive biomarkers for the immunogenic effects of autophagy inhibition. However, radiation does not enhance the effects of autophagy inhibition due to its induction of PD-L1, underscoring the need for combination treatments with ICB.

## Materials and methods

### Cell culture

The human TNBC cell lines MDA-MB-468, MDA-MB-436 and MDA-MB-231 were purchased from ATCC. All three cell lines were cultured in Dulbecco’s Modified Eagle Medium (DMEM) medium (Gibco, 41965-039), supplemented with 10% fetal bovine serum (FBS) (Sigma, F7524-500ml) and 1% penicillin/streptomycin (P/S) (Gibco, 15140-122). For autophagy induction experiments cells were incubated in unsupplemented EBSS starvation media (Gibco, 24010-043). All cell lines were maintained at 37 °C in a humidified atmosphere with 95% air and 5% CO_2_. Cells were grown to 70-80% of confluency at time of irradiation. Prior to irradiation, cell culture flasks were filled air-bubble free with unsupplemented medium to accommodate the vertical flask positioning in both irradiation workflows, which was replaced immediately after irradiation with fresh, supplemented medium.

### Irradiation and treatments

Cells were irradiated with 8 Gy of 200 kV X-rays or C-ions. Additionally, a fractionation regimen of 3×8 Gy was implemented for the X-ray experiments but not for C-ions due to limited beam time availability. X-ray irradiations were administered using a horizontal irradiation cabinet (YXLON, TU32-D03, 20 mA, 5.5FOC, filtration: 3 mm Be + 3 mm Al + 0.5 mm Cu), C-ion irradiations were all performed using the particle synchrotron at MedAustron with a horizontal experimental beam line. For C-ion experiments, customized cell holders were manufactured and dosimetrically verified to enable cell irradiations in water, in accordance with a recent report of a National Cancer Institute special panel [82]. The positioning uncertainty was estimated to be 0.3 mm [83]. A spread-out Bragg peak (SOBP) width of 4 cm and a range at the 80% dose level (R80) of 10.6 cm (corresponding to C-ion energy of 285 MeV/u) was achieved for both setups (ChamberFlask and T25 flasks). The dose-averaged LET (LET_D_) was derived from GATE/Geant4 based Monte Carlo simulations taking into account the beam nozzle of the experimental beam line at MedAustron. All primary and secondary particles were included in the calculation of the LET_D_. The scoring geometry was a cylinder with a diameter of approximately 80 mm. At 8 cm, the depth corresponding with the cell placement in the center of the SOBP, the LET_D_ is 58 keV/μm (Supplementary Fig. 11).

The chemotherapeutic agent Taxol (Bio-Techne, 1097) was used at a final concentration of 1 µM. The autophagy inhibitors SAR405 (TargetMol, T12831L) and bafilomycin A1 (Szabo-Scandic, SACSC-201550B) were used at concentrations of 3 µM (SAR405) and 200 nM (bafilomycin A1). The autophagy inducer Torin1 (MyBioSource, MBS3600848) was used at a concentration of 300 nM. The TBK1/IKKε inhibitor Amlexanox (TargetMol, T1639) was used at a concentration of 20 µM. The STING inhibitor H151 (TargetMol, T5674) and the NF-κB inhibitor BI-605906 (THP Medical Products, HY-13019) were used at 10 µM. Treatment durations are indicated in the respective graphs.

### Cell lysis, SDS-PAGE and Western blotting

Cell pellets were resuspended in lysis buffer (50 mM Tris-Cl pH 7,4, 250 mM NaCl, 5 mM EDTA, 50 mM NaF, 1 mM Na_3_VO_4,_ 1% NP40, 1 mM PMSF, 1% of protease and phosphatase inhibitor cocktail (Thermo Fisher Scientific, D12345) and 1x PhosStop (Merck, 4906837001)) and lysed for 20 min on ice before centrifugation at 4 °C for 10 min. Protein concentration in the supernatant was estimated by Bradford assay. 20 µg lysate per lane were loaded onto SDS-PAGE gels, transfer onto nitrocellulose membrane was performed in transfer buffer (25 mM Tris, 192 mM glycine) containing 10% ethanol at 35 V o/n. Membranes were blocked for 1 h and incubated in primary antibody o/n on a roller at 4 °C. Membranes were washed three times for 10 min in TBS-T (0.1% Tween in TBS), incubated in HRP-conjugated secondary antibody for 2 h at 4 °C and chemiluminescent signal was detected on ChemiDoc MP Imaging system (Bio-Rad) operated by Bio-Rad Image Lab Touch Software (version 2.3.0.07) and analyzed using Bio-Rad Image Lab Software (version 5.2.1). Antibodies used for Western blotting are listed in Supplementary Table 1. Uncropped blots are shown in Supplementary File. All Western blot experiments were performed in two biologically independent replicates.

### RNA isolation

Cell pellets were resuspended in 1 mL TRAzol (Neo-biotech). 200 µL chloroform (Applichem) was added and the lysate was vortexed and centrifuged at maximum speed (21100xg) for 15 min at 4 °C. The aqueous layer was transferred to a new tube and precipitated with 0.5 mL isopropanol. The RNA pellet was isolated by centrifugation for 30 min at 4 °C, washed with 1 mL 75% ethanol, re-centrifuged for 10 min, dried and resuspended in 70 µL RNase free water. 20 µg RNA was treated with 40 U DNaseI (Roche) for 30 min at 37 °C and subsequently purified by phenol-chloroform extraction and ethanol precipitation.

### Reverse transcription and real-time qPCR

1 µg of RNA was reverse transcribed using LunaScript RT SuperMix (NEB). cDNA was diluted 1:5 in H_2_O. qPCR was performed on a BioRad CFX Opus 96 cycler operated by BioRad CFX Maestro software (Version 2.3) using iQ^TM^ Multiplex Powermix (Bio-Rad). IFNB1, CXCL10, PD-L1, IL-6 and TBP levels were quantified by multiplexing RT-PCR based on commercially available primers (human IFNB1 UniqueAssayID: qHsaCEP0054112, human CXCL10 UniqueAssayID: qHsaCEP0053880, human IL-6 UniqueAssayID: qHsaCEP0051939, human TBP UniqueAssayID: qHsaCIP0036255, human PD-L1 UniqueAssayID: qHsaCIP0039192) qPCR data were analyzed and plotted using GraphPad Prism (9.1.1). Experiments were performed in three biological replicates and each sample was measured in three technical replicates.

### Immunofluorescence microscopy

For immunofluorescence analyses of dsDNA, 1 x 10^5^ cells (all cell lines) were seeded in Nunc^TM^ Lab-Tek^TM^ ChamberFlasks one day prior to irradiation treatment. Immunofluorescence experiments were performed 24 h post irradiation. Cells were fixed in 4% paraformaldehyde (Carl Roth), permeabilized with 0.1% Tween 20 (Sigma Aldrich) and 0.01% Triton X-100 (Sigma Aldrich) in PBS and blocked with 1% BSA (Sigma Aldrich) in PBS. Primary antibodies targeting dsDNA (Abcam, ab27156, 1:1000 dilution) or TFAM (Genetex, GTX103231, 1:500 dilution) were applied o/n at 4 °C, followed by Alexa Fluor 488-conjugated goat anti-rabbit IgG (Abcam, ab150077, 1:400 dilution) and Alexa Fluor 568-conjugated goat anti-mouse IgG (Abcam, ab175473, 1:500 dilution) incubation for 1 h at room temperature. Cells were mounted with DAPI-containing mounting medium (VectaShield, H-2000) and evaluated using fluorescence microscopy. Images were acquired with the LSM980 inverse confocal microscope (Zeiss) equipped with a Plan-Apochromat 63x/1.4 Oil DIC (WD 0.13 mm), a 405 nm laser diode (30 mW), a 488 nm laser diode (30 mW) and a 561 nm DPSS laser (25 mW). Appropriate laser power, excitation wavelength range and pinhole size were chosen for each channel in alignment with negative controls. All images were acquired with Zen software (version 3.3, Zeiss) and processed with Fiji (ImageJ). For dsDNA-TFAM colocalization measurements, ROIs were drawn around each cell and the threshold was set to 20000 (dsDNA) and 10000 (TFAM). Fluorescence and co-localization signals were analyzed with the “Analyze Particles” option in Fiji. For co-localization the number of overlapping objects with specifications of 0.05-2/0.1-1.0 (TFAM) and 0.05-2/0.1-1.0 (dsDNA) size/circularity were counted. To get the percentage of overlapping dsDNA-TFAM puncta the number of overlapping particles was divided by the number of dsDNA particles. Intensity of dsDNA was calculated as follows: integrated density – (area of the selected cell * mean fluorescence of the background). Experiments were performed in three biological replicates

For immunofluorescence analyses of pTBK1 and p62, Cells were grown in 24-well plates on sterilized glass coverslips #1.5 (0.17 mm) which were coated with 5 μg/mL fibronectin (Sigma) for 3 h at RT before seeding. After 24 h the cells were treated with 3 μM SAR405 for 48 h. Following the treatment, the cells were fixed in 4% paraformaldehyde at RT for 10 min and washed twice with PBS followed by permeabilization with 0,5% TritonX-100 in PBS for 10 min. Next, the coverslips were washed twice with PBS and transferred into a humidified incubation chamber. Coverslips were blocked with 3% BSA and 0,1% Tween in PBS for 20 minutes. The primary antibody solution (rabbit anti-pTBK1 diluted 1:400 and mouse anti-p62 diluted 1:500 in blocking solution) was applied afterwards, and the coverslips were incubated at RT for 2 h. After incubation, the coverslips were washed once with PBS and twice with 3% BSA and 0,1% Tween in PBS and incubated with the secondary antibody solution at RT for 1 h in the incubation chamber. After the incubation with the secondary antibody (goat anti-rabbit-AF488 diluted 1:500 and goat anti-mouse-AF568 diluted 1:500 in blocking solution), the coverslips were washed with PBS, 3% BSA and 0,1% Triton in PBS and then again with PBS. After washing, DAPI staining was applied for 10 min and immediately washed with PBS. Coverslips were washed with water and dried before being mounted with 5 μl of Prolonged Diamond onto a slide. After 48 h of hardening, the cover slips were sealed with clear nail polish. High resolution Airyscan images were acquired using a Zeiss LSM980 microscope operated with Zen blue 3.3 software and a M27 (WD 0.19 mm) objective. Laser intensities for each channel: pTBK1 = 0,8%/790 V; p62 = 0,1%/750 V; DAPI = 0,3%/800 V. Aggregate size and location analysis was performed by using the automated image analysis tool AggreCount in Fiji (ImageJ) [84]. During the analysis, the following settings were used: Lower = 30.000; Upper = 65535; perinuclear distance = 10; aggresome size = 4; minimum aggregate size = 0,2; maximum aggregate size = 20; minimum nuclei size = 50; nuclei strictness = 7; minimum cell size = 75; cell strictness = 7. Colocalization analysis was performed using the colocalization tool in the Zeiss ZEN 3.3 software, by drawing ROIs surrounding single cells and thresholding the Costes for pTBK1 at 300, p62 at 260, and for the positive control 25 for both channels. Experiments were performed in three biological replicates.

### Nuclear IRF3 staining

Cells were washed twice with 1xPBS, fixed and permeabilized using Fixation/Permeabilization Diluent and Concentrate (#00-5223, #00-5123, eBioscience) and Permeabilization buffer (#00-5523, eBioscience) before intracellular staining with APC-conjugated anti-IRF3 (#395906, BioLegend) for 30 min at room temperature. Samples were then washed twice with Permeabilization buffer and PBS sequentially before sorting on the Cytek Aurora CS^TM^ Cell Sorter. Untreated Cells (0 Gy) were used as negative controls. Experiments were performed in three biological replicates.

### γH2AX staining

γH2AX foci numbers were assessed 1 h, 24 h and 72 h after X-ray or C-ion irradiation as markers of DNA double strand breaks (DSB) and cell line specific DSB repair proficiency. Cells were seeded in a concentration of 1 x 10^5^ cells per chamberflask (Nunc^TM^ Lab-Tek^TM^) and irradiated with 0 Gy or 8 Gy of physical dose. For immunofluorescence detection of γH2AX foci, cells were fixed with 4% paraformaldehyde for 1 h at 4 °C. Further steps included permeabilization with 0.1% Triton X and 0.1% SDS in PBS for 10 min at room temperature, blocking with 2% BSA in PBS for 1 h at room temperature, primary antibody incubation (concentration 1:100, # 05-636 Merck Millipore) over night at 4 °C and secondary antibody incubation (Rhodamine (TRITC)-conjugated AffiniPure Goat Anti-Mouse IgG 1:400 (#115-025-072, Jackson Immuno Research Laboratories) for 1 h at room temperature. Slides were covered with VectaShield antifade mounting medium containing DAPI (#H-1200-10, VectorLaboratories), coverslipped and analyzed with a Zeiss A2 microscope (Zeiss) equipped with the automated Metafer analysis system (MetaSystems). The Metafer software preselects the brightest foci in each cell nucleus and further on disregards any signal that does not reach a minimum of 60% of signal intensity in the particular nucleus. A minimum of 150 cells were analyzed per slide. Experiments were performed in 3 biological replicates per condition. Analyses of γH2AX foci was performed using one-way analyses of variance (ANOVA) with post hoc Tukey´s multiple comparison tests. A value of p < 0.05 was regarded as statistically significant.

### Immunoprecipitation

For each immunoprecipitation 1 x 10^7^ cells were used. Cells were harvested 48 hours after treatment and lysed in lysis buffer (50 mM Tris pH 8, 150 mM NaCl, 1% Triton, 1x protease inhibitors, 1 mM DTT, 1 mM MgCl_2_ and 1 mM PMSF). Lysis was performed for 30 minutes at 4 °C. The lysates were centrifuged at 21130xg for 10 minutes. The protein concentration was determined using the Bradford assay, and samples were adjusted to the lowest concentration. The adjusted lysates were used for immunoprecipitation. Phosphorylated TBK1 was immunoprecipitated with a monoclonal phospho-TBK1 (Ser172) antibody (5483, Cell Signaling) conjugated to Dynabeads protein A (Invitrogen). Normal rabbit IgG (2729, Cell Signaling) was used as IgG control. 40 µl Dynabeads Protein A per sample were incubated in TBS with 2,82 µg antibody or rabbit IgG for 1 hour on a rotating wheel at room temperature. The beads were washed three times in TBS on a magnetic stand. Purified lysates were added to the beads and incubated for 2 hours at 4 °C on a rotating wheel for immunoprecipitation. After immunoprecipitation, the beads were washed three times with TBS. For elution, 40 µl of 2xSDS sample buffer was added to the beads and boiled at 95 °C for 5 minutes. The eluate was separated using a magnetic stand and subsequently analyzed by SDS-PAGE and Western blotting. Experiments were performed in two biological replicates.

### Isolation of peripheral blood mononuclear cells (PBMCs)

PBMCs from venous blood of three healthy human donors were obtained in accordance with the Declaration of Helsinki and approved by the Ethics Committee of the Medical University of Vienna (2001/2018, 1238/2024), isolated by density gradient centrifugation (800 g without break for 20 min) using Lymphoprep (Axis-Shield), washed twice with Ca^2+^/Mg^2+^-free phosphate buffered saline (PBS) and counted. After sedimentation by centrifugation (350 g, 5 min), PBMC aliquots were directly resuspended at a density of 2 x 10^6^ cells/mL in fresh cell-free cell culture supernatants collected from the TNBC cell lines after 72 h of culture. As a control, PBMCs were resuspended in fully supplemented DMEM medium. PBMCs were cultured at 37 °C in a humidified atmosphere with 5% CO_2_ for 20 h and then analyzed by spectral flow cytometry.

### Spectral flow cytometry

PBMCs were harvested and adherent cells were detached from the cell culture plates with ice-cold 1.5 mM EDTA in PBS. Cells were collected by centrifugation (350 g, 5 min), washed with PBS and stained with the LIVE/DEAD Fixable Blue Dead Cell Stain Kit (Thermo Fisher, #L23105, 1:500 final concentration) for 20 min at room temperature and washed twice with precooled staining buffer (PBS containing 1% BSA and 0.02% NaN3). To prevent non-specific binding of monoclonal antibodies (mAbs) to Fc receptors, cells were resuspended in a blocking buffer consisting of Brilliant Stain Buffer (BD Biosciences, #566349) supplemented with 3.2 mg/mL human IgG (Beriglobin P; CSL Behring) and 20 μL/mL True-Stain Monocyte Blocker (BioLegend, #426102), and incubated for 30 min on ice. Afterwards, 2 x 10^6^ cells were stained with the full immunophenotyping panel, detailed in Supplementary Table 2, consisting of 30 mAbs against surface antigens prediluted in Brilliant Stain Buffer. In parallel, 1 x 10^6^ cells were stained with a control lineage panel, consisting of 12 mAbs. To enhance staining of difficult-to-stain antigens, the prediluted chemokine receptor mAbs (against CCR7, CCR4, CCR5, CCR6 and CXCR3) and a TCRγδ mAb were added prior to adding a cocktail of the remaining 25 mAbs. After adding the mAb cocktail, cells were incubated 30 min on ice. Single stains, used as controls for unmixing, were performed in parallel with either Cytek FSP CompBeads (Cytek Biosciences, #B7-10011) or non-stained cells. After 30 min incubation, all samples were washed twice with a staining buffer and immediately acquired using a Cytek Aurora spectral flow cytometer equipped with SpectroFlo software (Cytek Biosciences). Data were unmixed using SpectroFlo software and further analysis was performed using FlowJo 10 software (BD Biosciences). All samples were pre-gated to remove artifacts, debris and doublets by evaluating the scatter profiles, and subsequently gated for individual PBMC subsets in accordance with the literature [85] and displayed in Supplementary Fig. 7. To evaluate the percentage of activated cells within individual PBMC lineages, data from the control lineage stain were used for setting the gates for individual activation markers, while the dendritic cell populations, due to absence of an HLA-DR mAb in the lineage stain, were evaluated using a monocyte control stain.

## Supplementary information

Original data_TNBC qPCR values

Original data_uncropped WB

Supplementary information

## Data Availability

Raw data obtained in this study are available in the file ‘Original data’. Materials used in this study are available upon request.
