# Supplementary material for: Molecular features of TNBC govern heterogeneity in the response to radiation and autophagy inhibition
Source: Cell Death Dis. 2025 Jul 21;16(1):540. doi: 10.1038/s41419-025-07873-w (PMC12280211; doi:10.1038/s41419-025-07873-w)
Supplement: Supplementary file 1 — Original data_TNBC qPCR values [file 41419_2025_7873_MOESM1_ESM.pdf]

Fig 2B MDA-MB-468

IFNb1		0Gy	8Gy	3x8Gy	SAR405	8Gy +SAR405	3x8Gy + SAR405
	it1	1	3,82	11,01	1,48	3,51	6,88
	it2	1	4,26	13,57	3,09	4,11	8,73
	it3	1	4	7,5	2,59	3,82	6,38
CXCL10		0Gy	8Gy	3x8Gy	SAR405	8Gy +SAR405	3x8Gy + SAR405
	it1	1	23,12	119,53	27,57	36,35	180,58
	it2	1	7,4	69,73	66,2	38,85	76,84
	it3	1	7,16	96,59	153,18	98,19	94,7
PD-L1		0Gy	8Gy	3x8Gy	SAR405	8Gy +SAR405	3x8Gy + SAR405
	it1	1	1,48	10,01	0,58	1,39	6,73
	it2	1	1,49	9,12	1,07	1,67	6,01
	it3	1	1,74	8,86	1,03	1,79	6,64
IL-6		0Gy	8Gy	3x8Gy	SAR405	8Gy +SAR405	3x8Gy + SAR405
	it1	1	2,01	56,8	0,41	2,15	32,34
	it2	1	2,43	100,85	0,92	3,36	56,94
	it3	1	1,96	60,72	1,36	2,27	46,68

IFNb1	DMSO	Taxol	SAR405	Taxol + SAR40	Bafilomycin A1	Taxol + Bafilomycin A1
it1	1	6,8	1,08	6,75	4,19	3,52
it2	1	7,47	1,71	4,34	5,02	4,93
it3	1	5,74	2,21	3,71	3,3	4,25
CXCL10	DMSO	Taxol	SAR405	Taxol + SAR40	Bafilomycin A1	Taxol + Bafilomycin A1
it1	1	115,29	52,32	186,18	11,82	15,2
it2	1	70,08	82,94	137,59	11,27	7,93
it3	1	100,4	194,41	258,45	32,75	15,01
PD-L1	DMSO	Taxol	SAR405	Taxol + SAR40	Bafilomycin A1	Taxol + Bafilomycin A1
it1	1	2,05	0,66	1,84	0,85	1
it2	1	1,72	0,91	1,7	1,06	1,15
it3	1	1,89	1,15	1,89	1,14	1,5
IL-6	DMSO	Taxol	SAR405	Taxol + SAR40	Bafilomycin A1	Taxol + Bafilomycin A1
it1	1	45,49	0,34	40,18	3,09	3,35
it2	1	46,39	1,5	26,55	3,73	4,89
it3	1	52,82	1,31	34,01	2,7	4,86

IFNb1	0Gy	8Gy C-ions	SAR405	8Gy C-ions +SAR405
it1	1	4,33	0,58	2,57
it2	1	5,66	0,63	3,4
it3	1	4,33	0,76	2,94
CXCL10	0Gy	8Gy C-ions	SAR405	8Gy C-ions +SAR405
it1	1	2	9,2	4,01
it2	1	2,72	10,43	5,55
it3	1	3,22	9,5	6,61
PD-L1	0Gy	8Gy C-ions	SAR405	8Gy C-ions +SAR405
it1	1	2,79	1,17	2,49
it2	1	3,96	1,55	2,93
it3	1	3,13	1,14	2,4
IL-6	0Gy	8Gy C-ions	SAR405	8Gy C-ions +SAR405
it1	1	19,45	1,03	10,57
it2	1	45,26	1,75	22,64
it3	1	27,83	1,1	17,48

Fig 3B MDA-MB-436

IFNb1

	0Gy	8Gy	3x8Gy	SAR405	8Gy +SAR405	x8Gy + SAR405
it1	1	1,63	1,36	1,96	2,7	2,45
it2	1	4,61	2,01	1,73	3,68	2,81
it3	1	3,34	1,15	1,4	3,59	2,24
it4						

DMSO	Taxol	SAR405	Taxol + SAR40	Bafilomycin A1	ol + Bafilomycin A1
1	3,66	1,79	5,64	2,82	2,27
1	3,66	2,38	5,84	3,45	3,23
1	3,99	2,56	7,82	2,56	3,34
1	1,95	1,45	5,66	1,75	2,43

0Gy	8Gy C-ions	SAR405	y C-ions +SAR405
1	7,35	1,52	8,32
1	7,28	1,67	8,17
1	6,46	1,41	9,06

CXCL10

	0Gy	8Gy	3x8Gy	SAR405	8Gy +SAR405	x8Gy + SAR405
it1	1	1,04	0,51	0,7	1	0,6
it2	1	1,17	0,91	1,48	1,83	1,15
it3	1	1,23	0,76	0,67	0,9	0,81
it4	1	1,02	0,37	0,64	0,88	0,65

DMSO	Taxol	SAR405	Taxol + SAR40	Bafilomycin A1	ol + Bafilomycin A1
1	1,09	1,69	1,23	0,94	0,76
1	1,29	1,47	1,74	0,81	1,01
1	0,95	0,58	1	0,26	0,41
1	0,91	0,84	0,74	0,21	0,38

0Gy	8Gy C-ions	SAR405	y C-ions +SAR405
1	1,39	0,9	1,3
1	1,15	0,82	1,09
1	1,23	0,85	1,37

PD-L1

	0Gy	8Gy	3x8Gy	SAR405	8Gy +SAR405	x8Gy + SAR405
it1	1	1,26	1,45	1,61	1,46	1,46
it2	1	1,11	1,15	2,01	1,78	1,69
it3	1	2	1,52	2,44	2,34	1,86
it4	1	1,59	1,19	1,7	1,91	1,92

DMSO	Taxol	SAR405	Taxol + SAR40	Bafilomycin A1	ol + Bafilomycin A1
1	0,83	1,23	0,95	1,25	0,89
1	1,09	1,61	1,47	1,28	1,1
1	1,92	2,44	2,15	1,59	1,37
1	1,49	1,86	1,45	1,23	1,06

0Gy	8Gy C-ions	SAR405	y C-ions +SAR405
1	2,42	2,07	2,91
1	2,22	1,75	2,65
1	2,48	1,81	2,96

IL-6

	0Gy	8Gy	3x8Gy	SAR405	8Gy +SAR405	x8Gy + SAR405
it1	1	1,45	2,2	3,12	2,66	4,56
it2	1	1,01	0,36	2,57	2,68	1,14
it3	1	1,17	1,06	2,01	2,08	2,1
it4	1	0,54	0,12	1,37	1,73	0,47

DMSO	Taxol	SAR405	Taxol + SAR40	Bafilomycin A1	ol + Bafilomycin A1
1	1,85	4,28	3,86	0,75	0,78
1	0,76	1,49	1,6	0,27	0,26
1	0,66	1,2	1,41	0,11	0,12
1	0,97	2,11	1,64	0,17	0,14

0Gy	8Gy C-ions	SAR405	y C-ions +SAR405
1	3,67	6	6,55
1	3,67	12,76	11,9
1	4,55	16,33	18,15

Fig 4B MDA-MB-231

CXCL10		0Gy	8Gy	3x8Gy	SAR405	8Gy +SAR405	x8Gy + SAR405
	it1	1	46,16	3,84	15,53	5,92	28,88
	it2	1	24,83	6,86	42,3	15,92	78,88
	it3	1	70,96	5,34	62,06	5,5	25,47

CXCL10	DMSO	Taxol	SAR405	Taxol + SAR40	Bafilomycin A1	ol + Bafilomycin A1
it1	1	0,99	6,45	0	2,69	0,02
it2	1	5,15	12	0,48	14,56	1,05
it3	1	6,54	11,58	0,04	12,48	0,51

CXCL10	0Gy	8Gy C-ions	SAR405	y C-ions +SAR405
it1	1	3,28	49,48	68,93054
it2	1	3,2	49,54	76,02
it3	1	2,57	35,97	55,44

PD-L1		0Gy	8Gy	3x8Gy	SAR405	8Gy +SAR405	x8Gy + SAR405
	it1	1	2,89	3,04	1,09	1,85	1,81
	it2	1	0,85	1,2	1,06	0,97	1,17
	it3	1	2,23	2,73	2,36	3,26	2,89

PD-L1	DMSO	Taxol	SAR405	Taxol + SAR40	Bafilomycin A1	ol + Bafilomycin A1
it1	1	2,64	2,88	2,44	1,13	3,28
it2	1	1,56	0,73	1,22	0,93	1,83
it3	1	2,95	1,35	2,45	1,56	3,29

PD-L1	0Gy	8Gy C-ions	SAR405	y C-ions +SAR405
it1	1	1,06	0,77	0,93
it2	1	1,13	0,86	0,97
it3	1	1,16	0,72	0,89

IL-6		0Gy	8Gy	3x8Gy	SAR405	8Gy +SAR405	x8Gy + SAR405
	it1	1	272,21	30,58	133,93	27,4	135,12
	it2	1	83,62	26,54	109,12	34,87	211,22
	it3	1	296,89	39,78	390,86	19,46	140,67

IL-6	DMSO	Taxol	SAR405	Taxol + SAR40	Bafilomycin A1	ol + Bafilomycin A1
it1	1	11,61	69,3	4,38	24,45	13,85
it2	1	12,3	16,82	4,18	20,86	11,77
it3	1	28,01	90,95	4,45	34,7	13,48

IL-6	0Gy	8Gy C-ions	SAR405	y C-ions +SAR405
it1	1	2,74	169,26	521,62
it2	1	2,26	185,56	722,61
it3	1	2,12	95,24	778,21

Fig 6	MDA-MB-468								
IFNB1		DMSO	H151	Amlexanox	BI605906	SAR405	SAR405+H151	SAR405+Amlexanox	SAR405+BI605906
	it1	1	1,32	0,94	0,92	11,14	8,1	11,88	9,45
	it2	1	1,52	1,1	0,61	12,94	14,87	21,08	8,03
	it3	1	1,37	1,29	0,78	17,62	13,47	20,88	13,44
CXCL10		DMSO	H151	Amlexanox	BI605906	SAR405	SAR405+H151	SAR405+Amlexanox	SAR405+BI605906
	it1	1	0,32	2,01	0,1	8,73	4,35	11,14	1,69
	it2	1	0,34	1,47	0,07	12,61	4,96	12,65	1,15
	it3	1	0,46	1,81	0,1	12,68	4,95	14,69	3,48
PD-L1		DMSO	H151	Amlexanox	BI605906	SAR405	SAR405+H151	SAR405+Amlexanox	SAR405+BI605906
	it1	1	1,05	0,8	0,96	9,68	4,15	6,85	7,68
	it2	1	1,27	0,89	1,02	10,29	6,22	11,37	8,94
	it3	1	0,94	1,12	1,03	9,07	5,29	7,96	7,48
IL-6		DMSO	H151	Amlexanox	BI605906	SAR405	SAR405+H151	SAR405+Amlexanox	SAR405+BI605906
	it1	1	0,72	3,17	1,2	152,67	57,84	114,35	254,81
	it2	1	1,15	3,95	2,62	216,9	70,65	142,53	207,73
	it3	1	1,34	4,37	0,87	142,06	63,23	110,46	293,44

	MDA-MB-436								
IFNB1		DMSO	H151	Amlexanox	BI605906	SAR405	SAR405+H151	SAR405+Amlexanox	SAR405+BI605906
	it1	1	1,38	0,61	0,36	4,57	6,28	5,12	1,67
	it2	1	0,99	1,02	0,49	5,19	6,26	7,13	2,23
	it3	1	1,26	0,99	0,44	5,53	6,55	6,49	2,01
CXCL10		DMSO	H151	Amlexanox	BI605906	SAR405	SAR405+H151	SAR405+Amlexanox	SAR405+BI605906
	it1	1	0,45	0,55	0,13	0,92	0,7	0,78	0,24
	it2	1	0,32	0,71	0,11	0,9	0,52	0,81	0,22
	it3	1	0,42	0,86	0,13	0,82	0,71	1	0,25
PD-L1		DMSO	H151	Amlexanox	BI605906	SAR405	SAR405+H151	SAR405+Amlexanox	SAR405+BI605906
	it1	1	1	0,49	0,91	2,34	2,7	1,59	2,21
	it2	1	0,98	0,68	1,06	1,9	2,05	1,91	2,07
	it3	1	1,07	0,72	1,05	2,31	2,89	2,12	2,44
IL-6		DMSO	H151	Amlexanox	BI605906	SAR405	SAR405+H151	SAR405+Amlexanox	SAR405+BI605906
	it1	1	0,37	0,33	0,52	1,63	1,51	2,04	0,72
	it2	1	0,73	0,87	0,38	1,78	1,96	2,56	0,94
	it3	1	0,53	1,39	0,45	3,29	1,25	2,06	0,9

	MDA-MB-231								
CXCL10		DMSO	H151	Amlexanox	BI605906	SAR405	SAR405+H151	SAR405+Amlexanox	SAR405+BI605906
	it1	1	3,1	5,09	0,25	42,79	13,13	45,49	9,45
	it2	1	2,82	4,26	0,26	39,89	12,63	45,19	8,99
	it3	1	1,05	1,96	2,45	34,3	6,7	42,71	5,22
PD-L1		DMSO	H151	Amlexanox	BI605906	SAR405	SAR405+H151	SAR405+Amlexanox	SAR405+BI605906
	it1	1	1,37	1,03	0,96	0,7	0,71	0,66	0,61
	it2	1	1,87	1,42	1,21	0,79	0,9	0,77	0,53
	it3	1	1,47	1,12	1,05	0,62	0,75	0,69	0,62
IL-6		DMSO	H151	Amlexanox	BI605906	SAR405	SAR405+H151	SAR405+Amlexanox	SAR405+BI605906
	it1	1	22,4	8,1	2,25	106,33	69,9	84,52	11,89
	it2	1	13,72	3,92	0,82	97,47	40,09	65,48	8,73
	it3	1	7,67	2,78	0,81	73,6	26,08	50,86	3,77

Sup Fig 1

MDA-MB-468

IFNb1	0Gy	8Gy D1	8Gy D2	8Gy D3	8Gy D4	8Gy D5	3x8Gy D1	3x8Gy D2	3x8Gy D3	DMSO	Taxol D1	Taxol D2	Taxol D3
it1	1	0,48	0,97	0,74	1,37	1,88	3,38	19,08	3,86	1	0,97	6,07	1,42
it2	1	0,39	1,09	2,53	1,36	3,18	1,26	2,96	4,94	1	1,06	1,96	4,03
it3	1	3,41	21,15	4,64	5	8,27	4,96	5,1	2,72	1	1	0,74	0,64

CXCL10	0Gy	8Gy D1	8Gy D2	8Gy D3	8Gy D4	8Gy D5	3x8Gy D1	3x8Gy D2	3x8Gy D3	DMSO	Taxol D1	Taxol D2	Taxol D3
it1	1	0,02	1,52	2,93	1,48	1,27	1,73	22,36	8,8	1	2,67	17,35	17,86
it2	1	0,02	0,75	3,19	1,49	2,86	3,65	22,27	32,58	1	1,46	21,75	29,86
it3	1	0,01	2,88	3,83	4,87	4,08	1,12	11,45	9,68	1	2,67	8,43	24,87

PD-L1	0Gy	8Gy D1	8Gy D2	8Gy D3	8Gy D4	8Gy D5	3x8Gy D1	3x8Gy D2	3x8Gy D3	DMSO	Taxol D1	Taxol D2	Taxol D3
it1	1	1,34	1,34	1,1	1,98	2,54	4,31	27,7	9,93	1	0,47	5,42	4,8
it2	1	0,53	0,87	1,45	1,06	2,63	1,8	4,73	8,63	1	0,35	4,01	13,44
it3	1	6,44	14,05	2,81	4,35	6	5,63	6,37	8,39	1	0,77	2,31	8,96

IL-6	0Gy	8Gy D1	8Gy D2	8Gy D3	8Gy D4	8Gy D5	3x8Gy D1	3x8Gy D2	3x8Gy D3	DMSO	Taxol D1	Taxol D2	Taxol D3
it1	1	9,45	6	11,53	1,82	2,62	105,36	898,74	310,51	1	52,61	1100,08	222,66
it2	1	1,37	4,86	14,36	7,76	12,83	158,64	1070,99	1844,84	1	144,47	363,12	904,33
it3	1	5,49	22,74	11,27	45,08	25,21	11,37	61,23	1951,71	1	29,16	939,74	2205,05

MDA-MB-436

IFNb1	0Gy	8Gy D1	8Gy D2	8Gy D3	8Gy D4	8Gy D5	3x8Gy D1	3x8Gy D2	3x8Gy D3	DMSO	Taxol D1	Taxol D2	Taxol D3
it1	1	0,57	4,56	3,84	1,72	2,92	1,06	0,47	1,08	1	0,66	5,51	6,8
it2	1	0,36	1,94	1,82	2,06	1,32	0,66	0,46	0,83	1	2,76	3,71	4,71
it3	1	4,75	3,89	2,78	2,64	3,13	2,61	0,55	0,9	1	2,07	8,77	1,96

CXCL10	0Gy	8Gy D1	8Gy D2	8Gy D3	8Gy D4	8Gy D5	3x8Gy D1	3x8Gy D2	3x8Gy D3	DMSO	Taxol D1	Taxol D2	Taxol D3
it1	1	0,51	1,6	1,07	0,54	0,72	0,32	0,13	0,15	1	0,14	0,55	1,49
it2	1	0,96	3,66	2,55	4,11	3,95	0,83	0,39	0,68	1	0,69	0,6	1,03
it3	1	0,96	1,26	0,67	0,45	0,43	0,63	0,15	0,14	1	0,49	1,14	0,57

PD-L1	0Gy	8Gy D1	8Gy D2	8Gy D3	8Gy D4	8Gy D5	3x8Gy D1	3x8Gy D2	3x8Gy D3	DMSO	Taxol D1	Taxol D2	Taxol D3
it1	1	1,18	1,68	1,8	1,71	2,06	1,34	1,51	1,93	1	0,76	1,64	2,16
it2	1	0,71	0,8	1,19	1,16	1,54	1,07	1,32	2,08	1	2,39	1,38	2,7
it3	1	6,62	1,06	0,84	0,92	1,11	2,72	0,53	0,85	1	1,17	1,69	0,76

IL-6	0Gy	8Gy D1	8Gy D2	8Gy D3	8Gy D4	8Gy D5	3x8Gy D1	3x8Gy D2	3x8Gy D3	DMSO	Taxol D1	Taxol D2	Taxol D3
it1	1	2,59	2,34	3,51	0,83	1,3	2,16	0,95	1,46	1	0,16	0,69	3,48
it2	1	0,82	0,91	2,42	1,37	1,75	1,14	3,12	2,56	1	2,66	0,92	1,08
it3	1	3,47	2,09	1,98	1,93	2,08	2,61	0,68	0,83	1	0,08	0,47	0,69

MDA-MB-231

CXCL10	0Gy	8Gy D1	8Gy D2	8Gy D3	8Gy D4	8Gy D5	3x8Gy D1	3x8Gy D2	3x8Gy D3	DMSO	Taxol D1	Taxol D2	Taxol D3
it1	1	0,12	9,1	16,42	14,12	15,87	5,16	20,4	31,53	1	0,78	42,15	100,2
it2	1	0,01	3,08	14,3	15,62	6,91	2,06	16,51	15,05	1	3,34	65,23	37,77
it3	1	0,02	2,59	20,02	21,08	48,78	6,53	25,45	21	1	1,2	68,53	121,61

PD-L1	0Gy	8Gy D1	8Gy D2	8Gy D3	8Gy D4	8Gy D5	3x8Gy D1	3x8Gy D2	3x8Gy D3	DMSO	Taxol D1	Taxol D2	Taxol D3
it1	1	0,02	0,02	0,03	0,03	0,03	0,65	0,51	0,71	1	0,91	1,44	1,21
it2	1	0,79	0,85	1,23	1,78	1,01	1,13	2,84	4,35	1	1,57	3,9	0,93
it3	1	0,7	0,36	0,49	0,43	0,86	1,79	1,76	1,53	1	0,71	5,18	1,61

IL-6	0Gy	8Gy D1	8Gy D2	8Gy D3	8Gy D4	8Gy D5	3x8Gy D1	3x8Gy D2	3x8Gy D3	DMSO	Taxol D1	Taxol D2	Taxol D3
it1	1	0,01	0,03	0,16	0,12	0,24	0,17	0,33	0,33	1	0,14	0,46	10,46
it2	1	0,45	1,09	5,55	13,58	5,5	0,99	4,96	10,23	1	3,19	6,12	9,81
it3	1	0,12	0,06	1,17	1,94	4,67	6,91	8,9	36	1	3,07	28,96	19,37
