# Supplementary material for: Molecular features of TNBC govern heterogeneity in the response to radiation and autophagy inhibition
Source: Cell Death Dis. 2025 Jul 21;16(1):540. doi: 10.1038/s41419-025-07873-w (PMC12280211; doi:10.1038/s41419-025-07873-w)
Supplement: Supplementary file 2 — Original data_uncropped WB [file 41419_2025_7873_MOESM2_ESM.pdf]

Fig. 1D

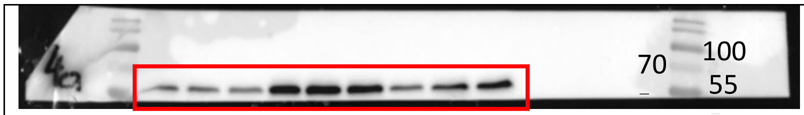	p62
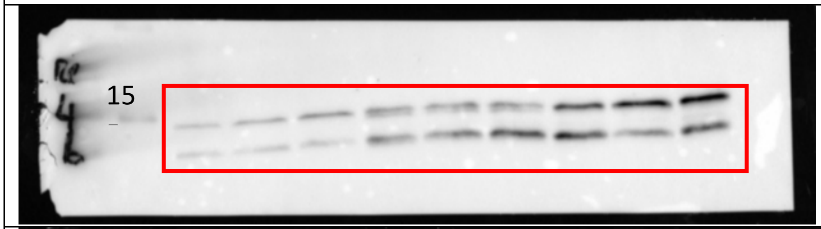	LC3
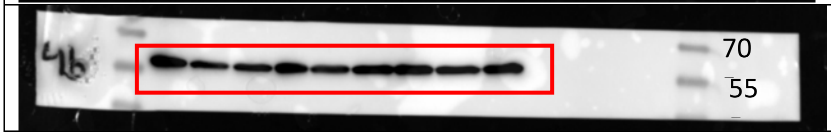	GAPDH

Fig. 2A

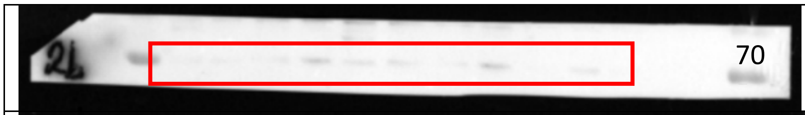	cGAS
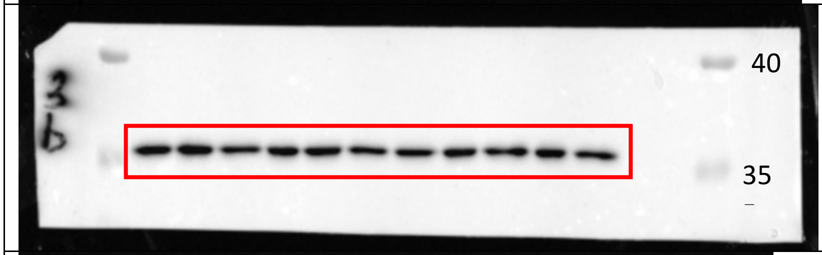	STING
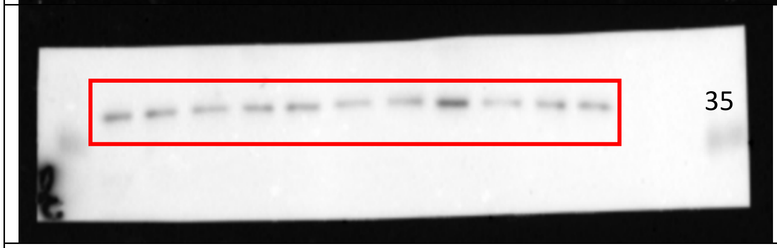	pSTING
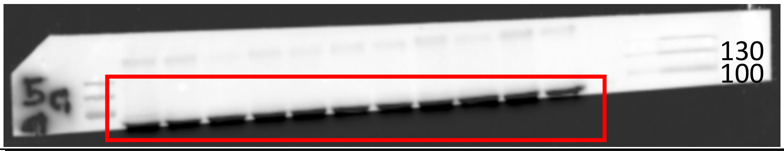	TBK1
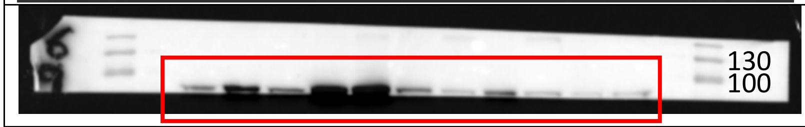	pTBK1
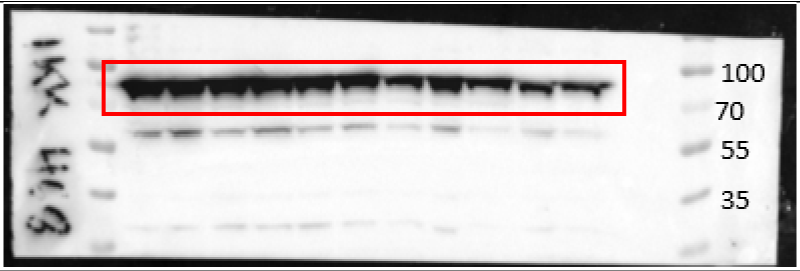	IKKϵ
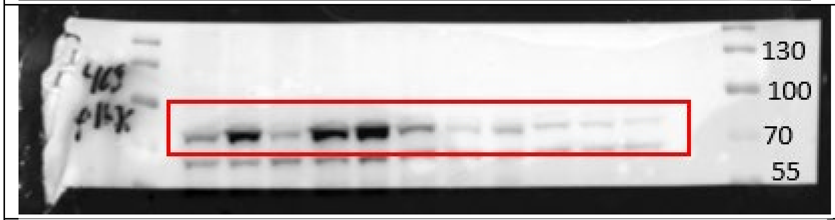	pIKKϵ
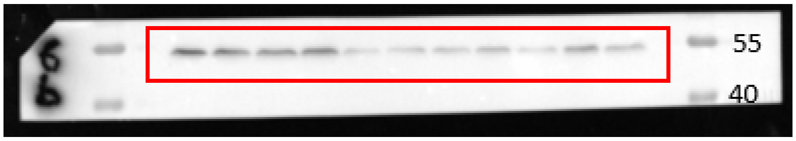	IRF3

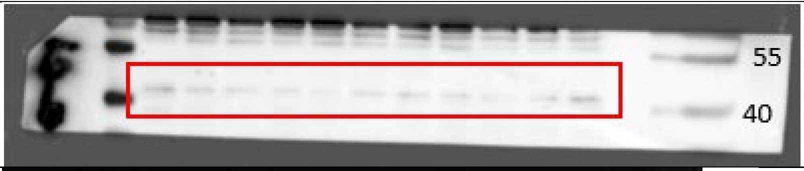	pIRF3
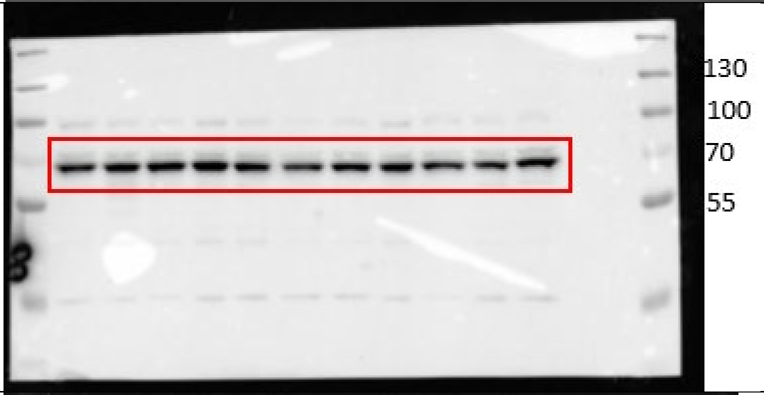	RelA
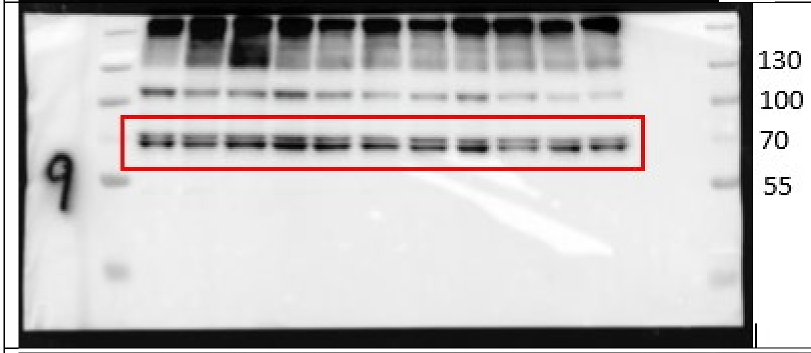	pRelA
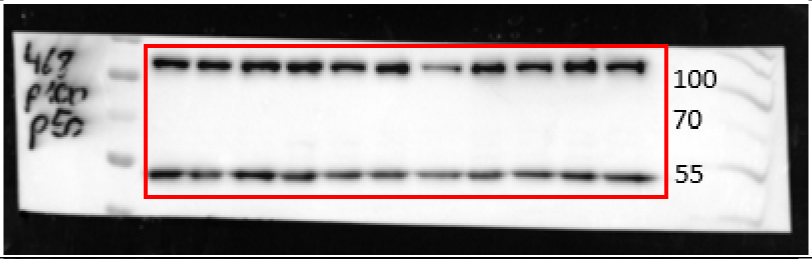	p100/p52
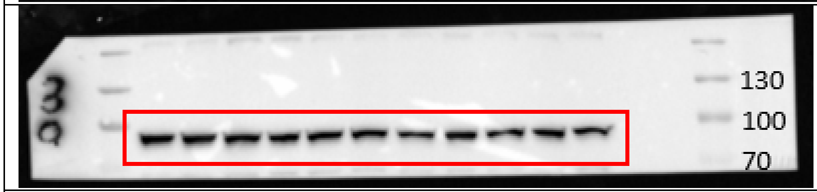	STAT1
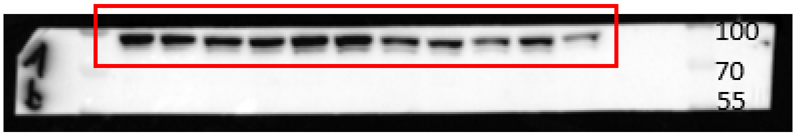	pSTAT1
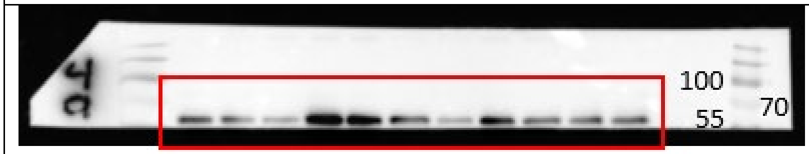	p62

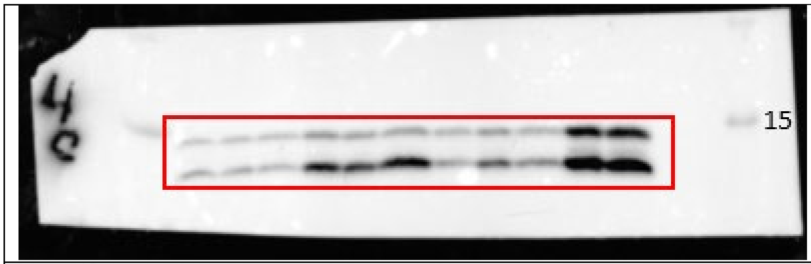	LC3
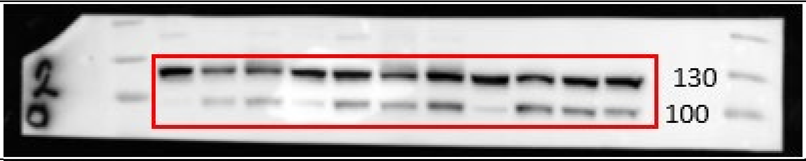	PARP1
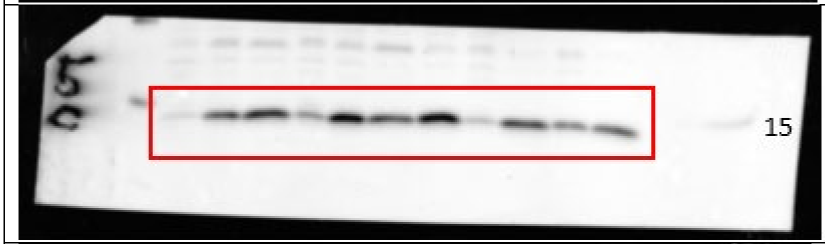	γH2AX
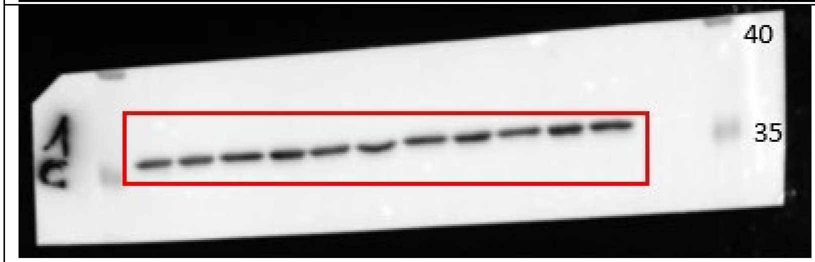	GAPDH

Fig. 3A

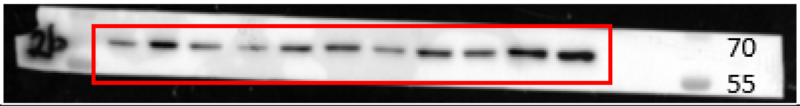	cGAS
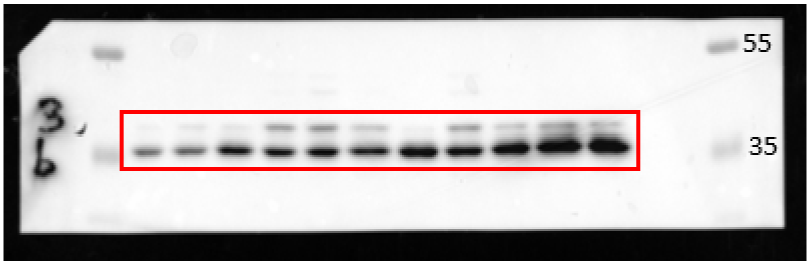	STING
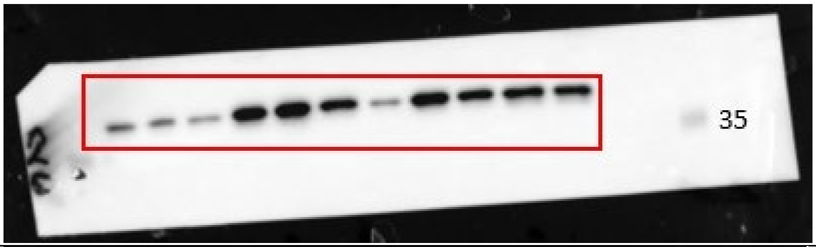	pSTING
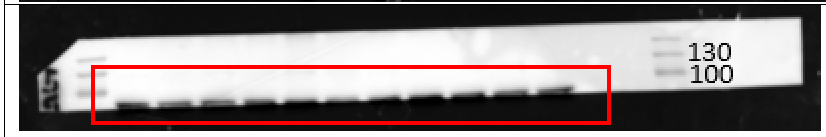	TBK1
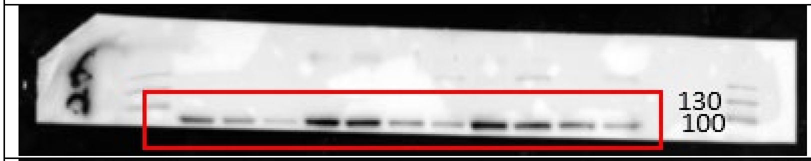	pTBK1
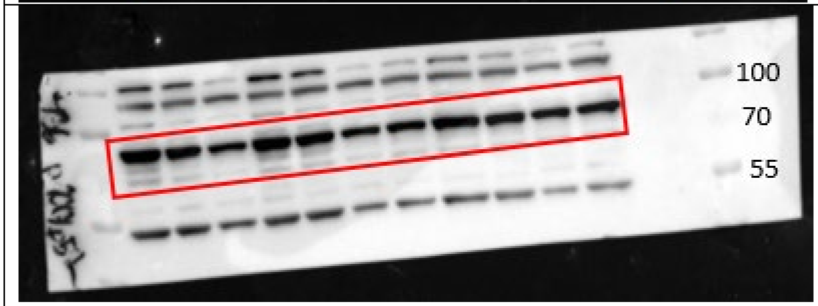	IKKϵ
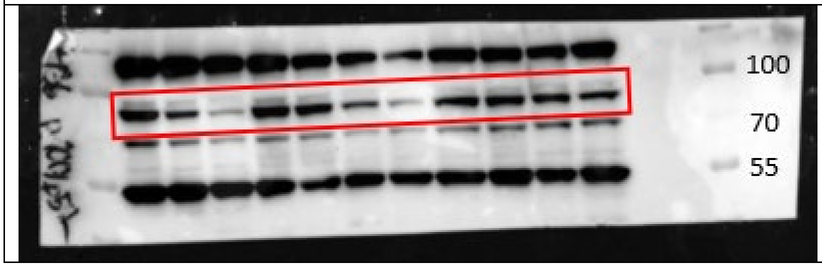	pIKKϵ

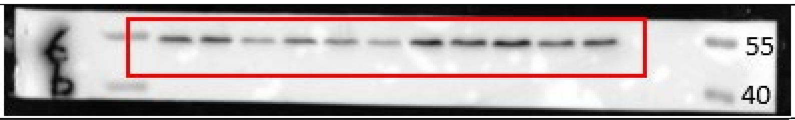	IRF3
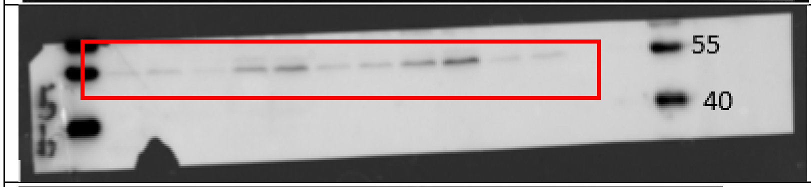	pIRF3
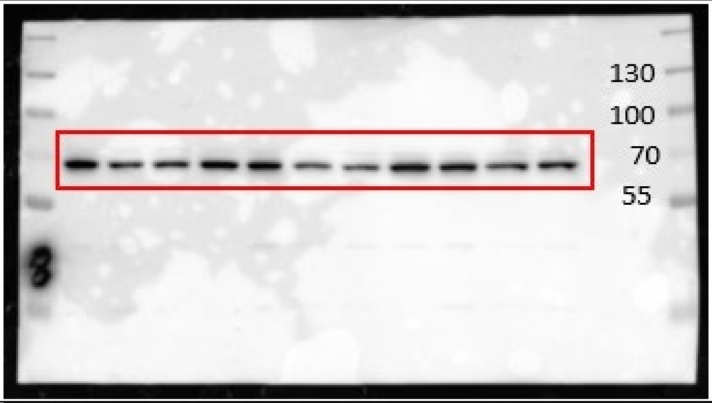	RelA
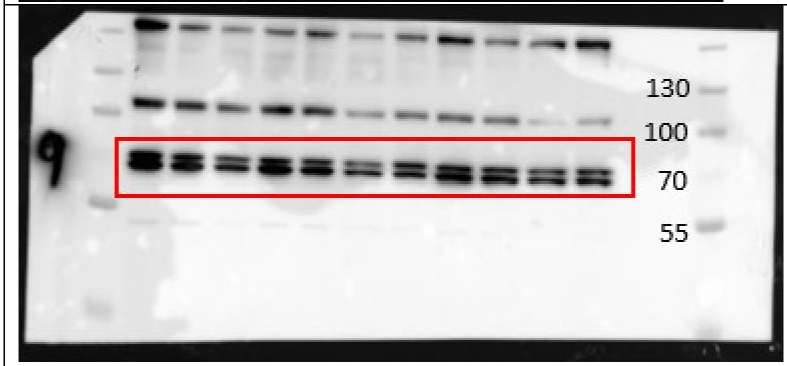	pRelA
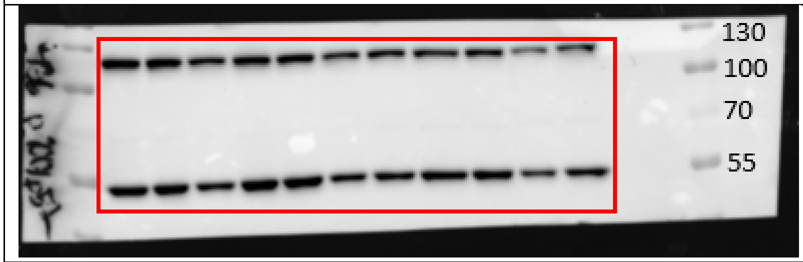	p100/p52
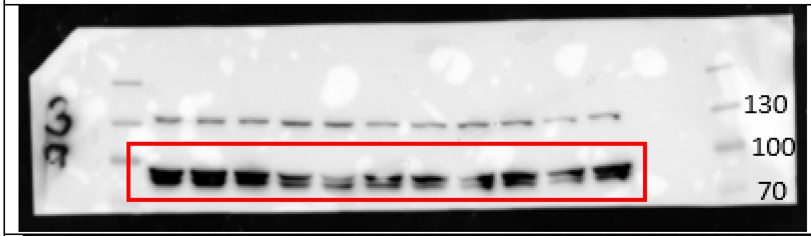	STAT1
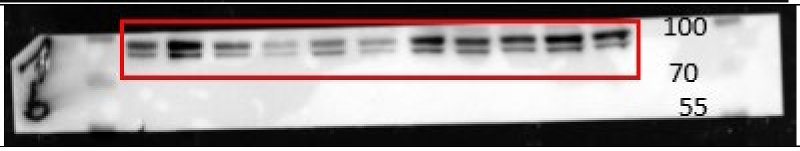	pSTAT1

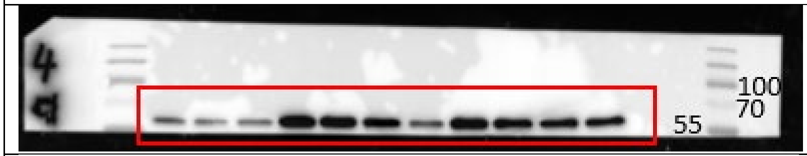	p62
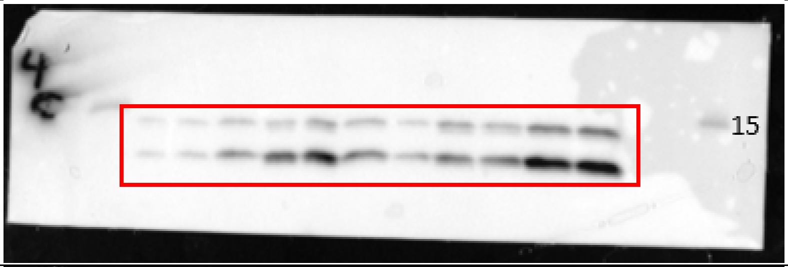	LC3
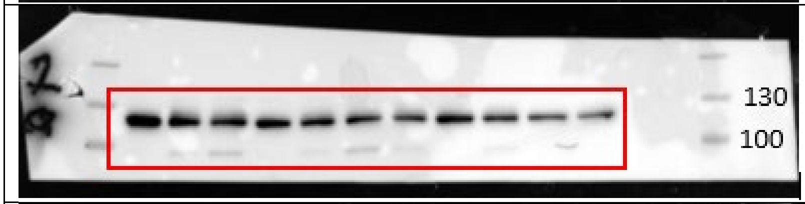	PARP1
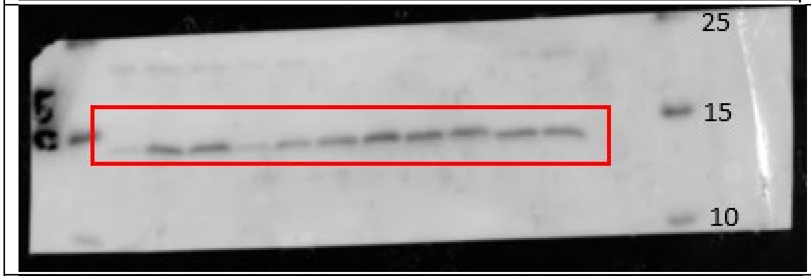	γH2AX
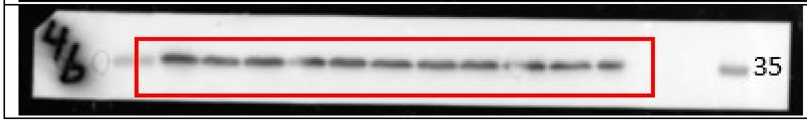	GAPDH

Fig 4A

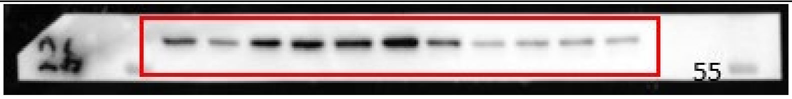	cGAS
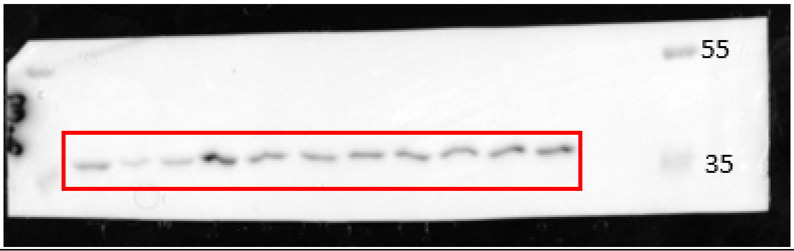	STING
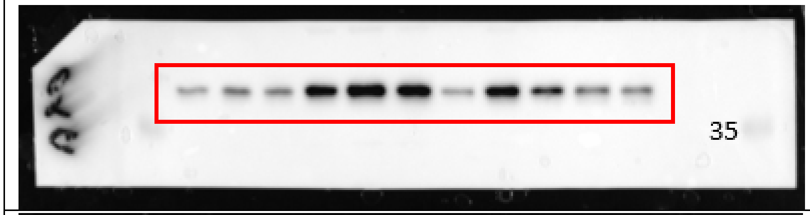	pSTING
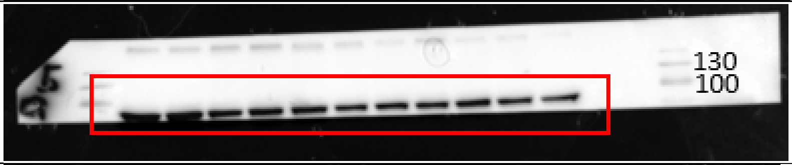	TBK1
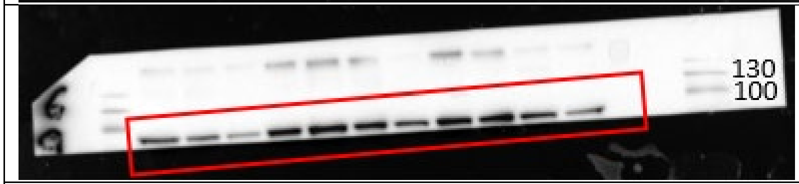	pTBK1
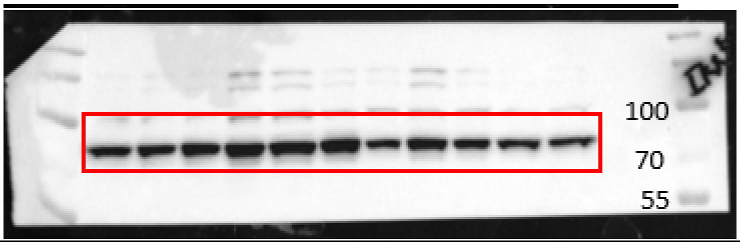	IKKϵ
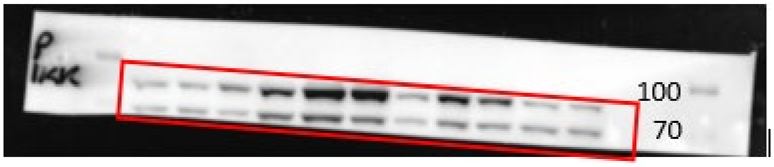	pIKKϵ
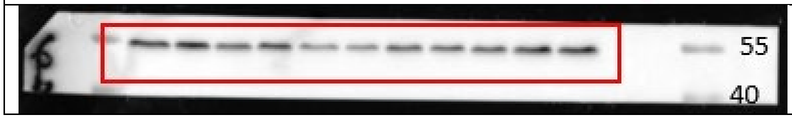	IRF3

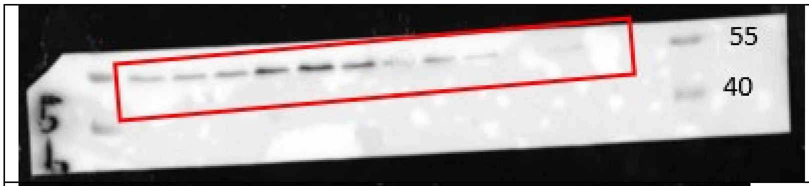	pIRF3
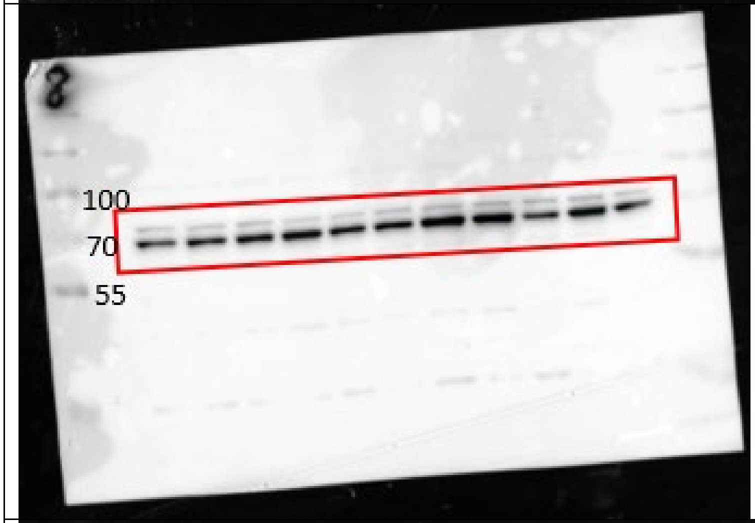	RelA
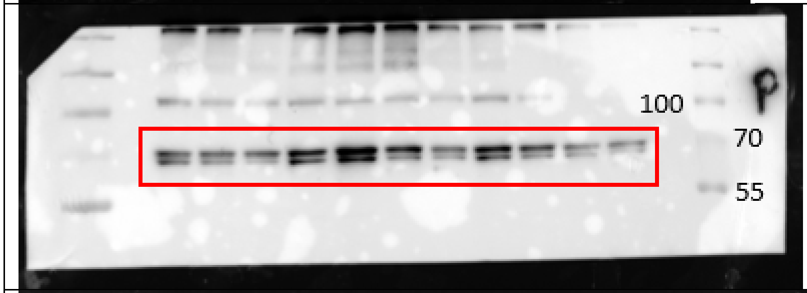	pRelA
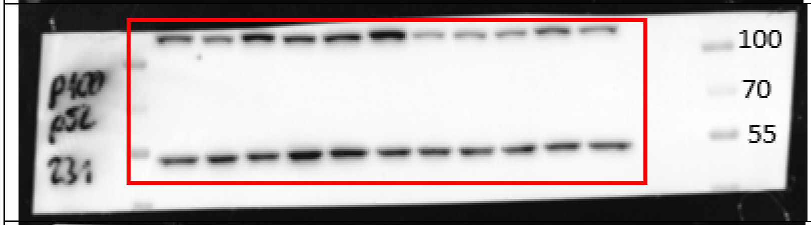	p100/p52
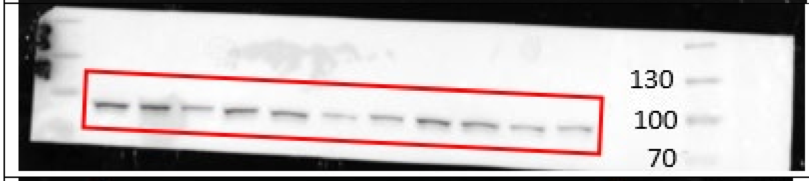	STAT1
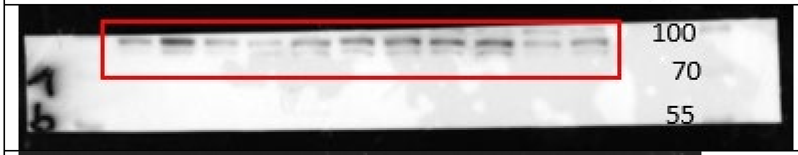	pSTAT1
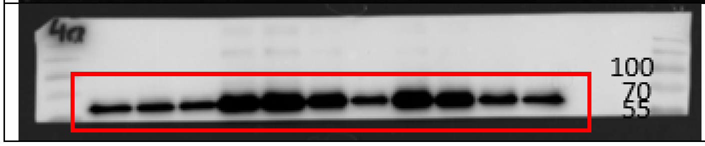	p62

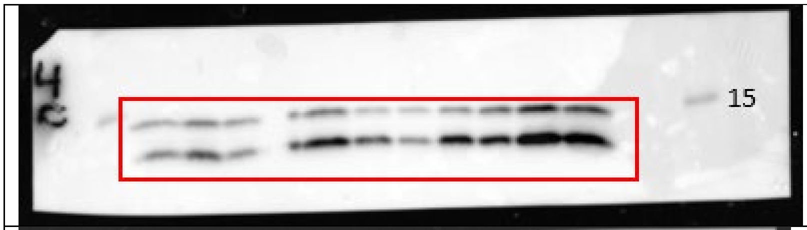	LC3
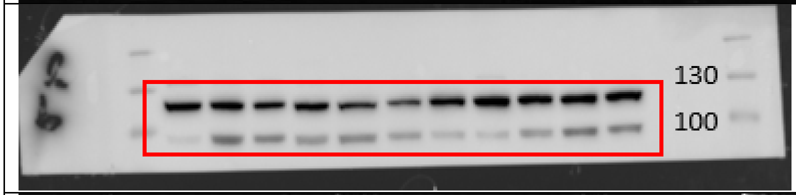	PARP1
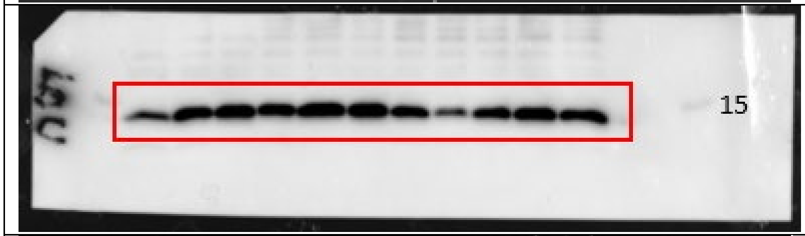	γH2AX
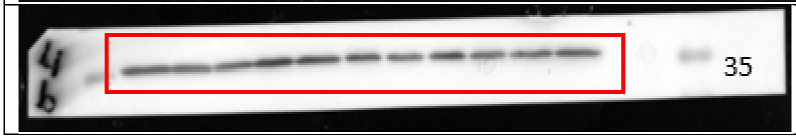	GAPDH

Fig. 5A

INPUT	
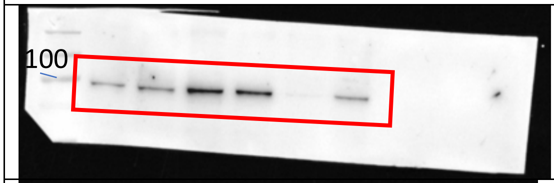	pTBK1
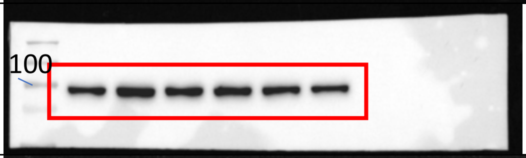	TBK1
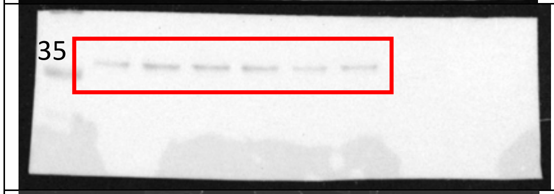	STING
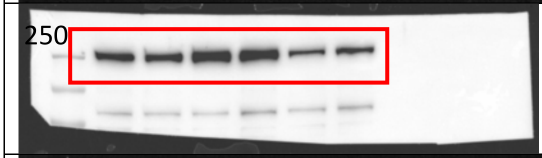	FIP200
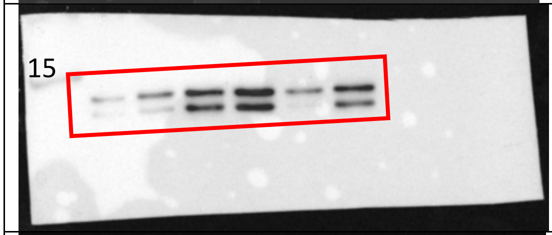	LC3
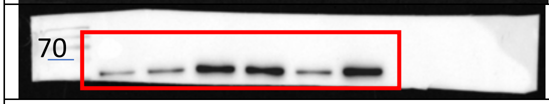	P62
ELUATE	
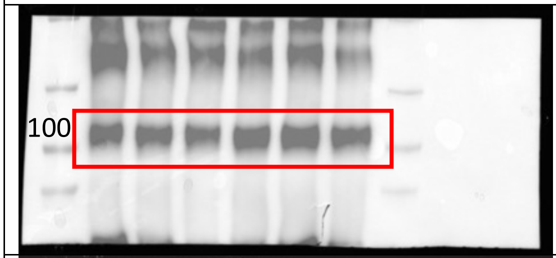	pTBK1
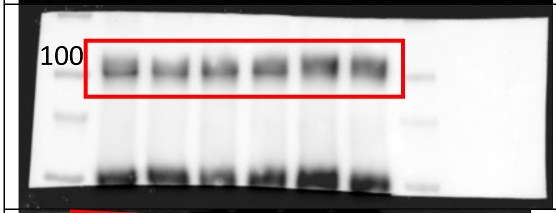	TBK1
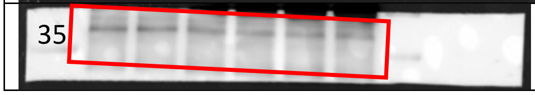	STING

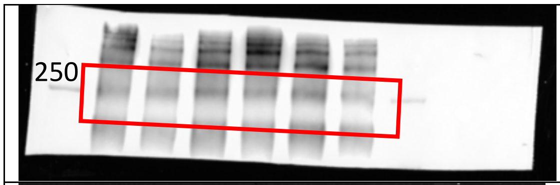	FIP200
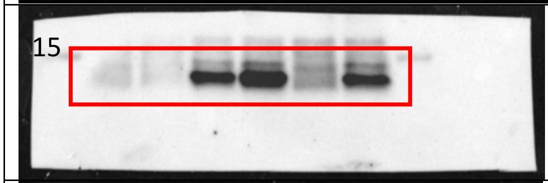	LC3
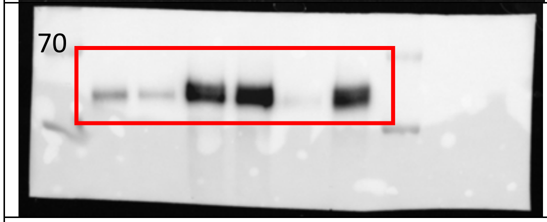	P62
IgG Control	
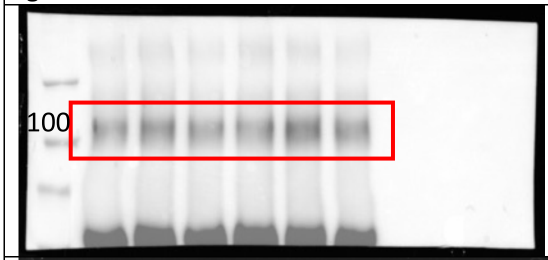	pTBK1
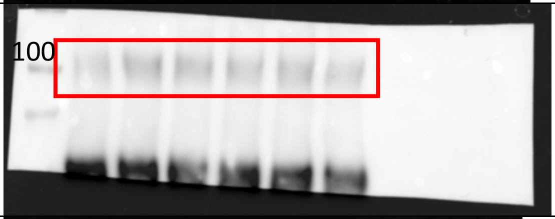	TBK1
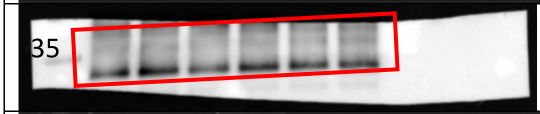	STING
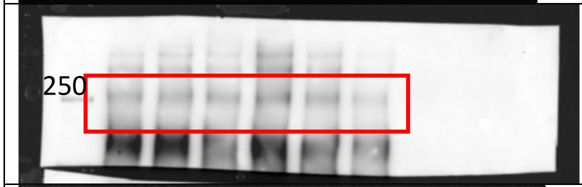	FIP200
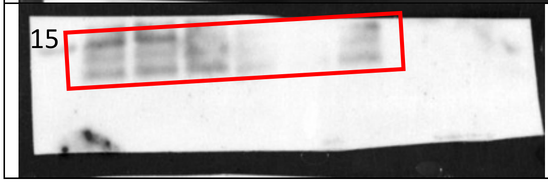	LC3

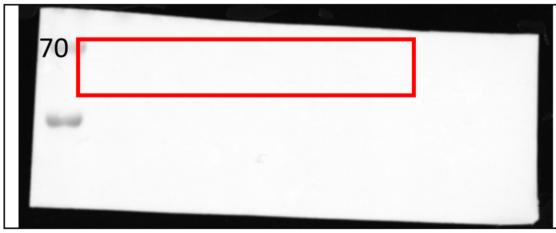		P62

Supplementary Fig. 2A

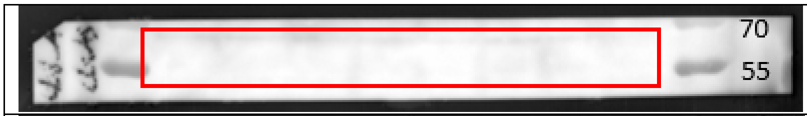	cGAS
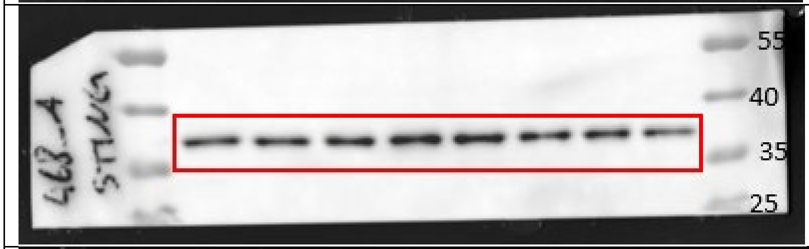	STING
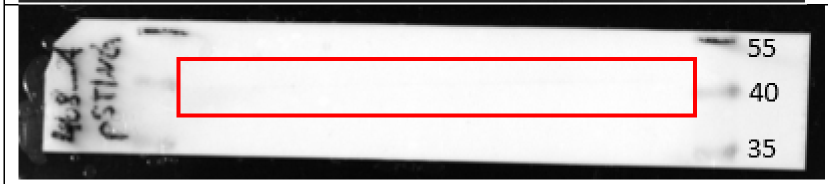	pSTING
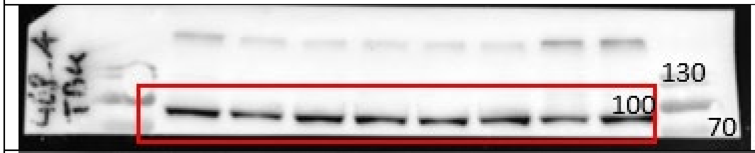	TBK1
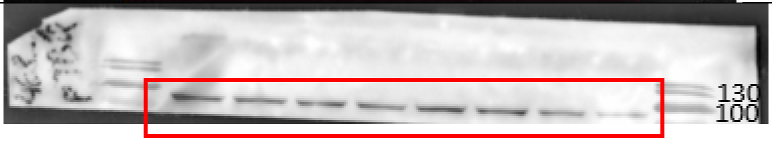	pTBK1
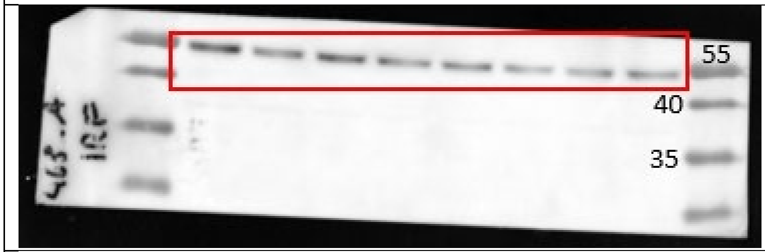	IRF3
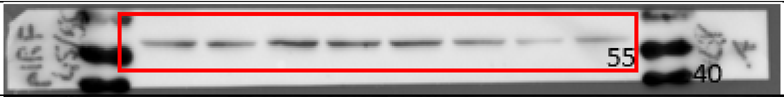	pIRF3
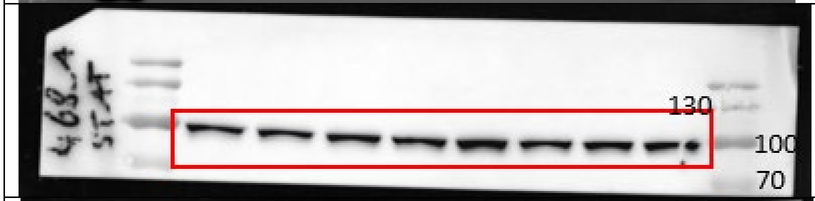	STAT1
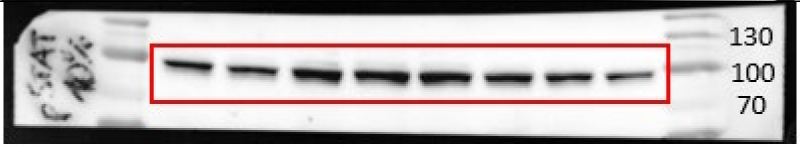	pSTAT1
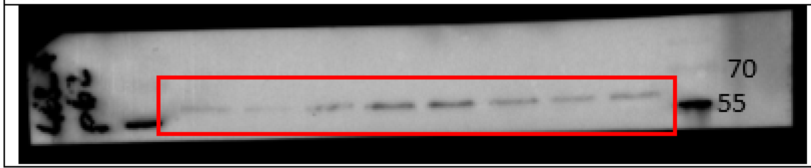	p62

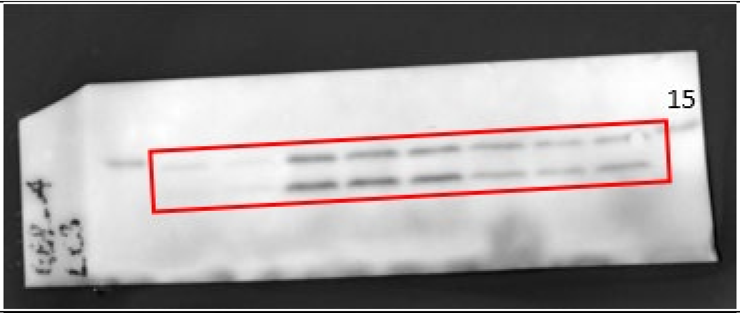	LC3
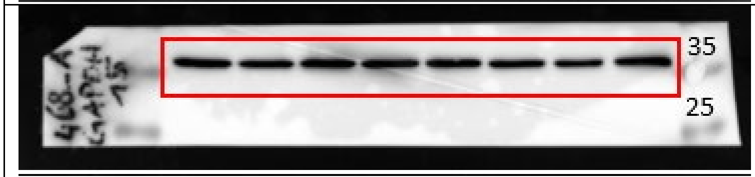	GAPDH

Supplementary Fig 2B

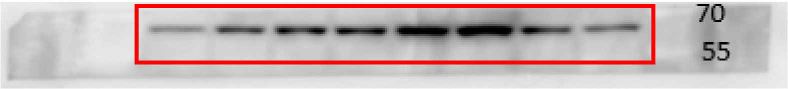	cGAS
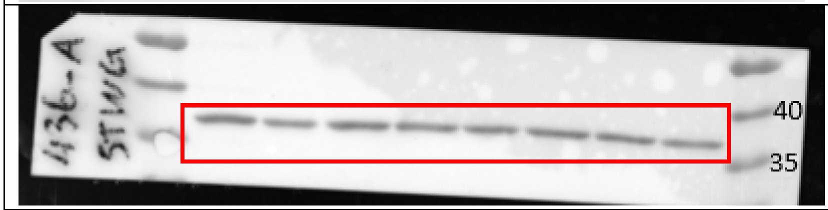	STING
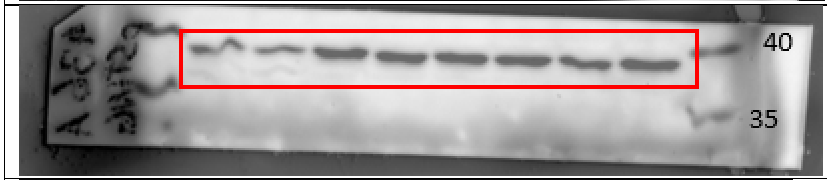	pSTING
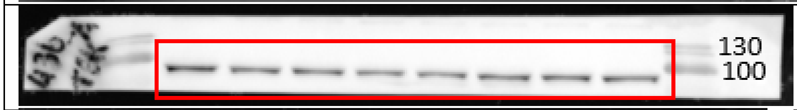	TBK1
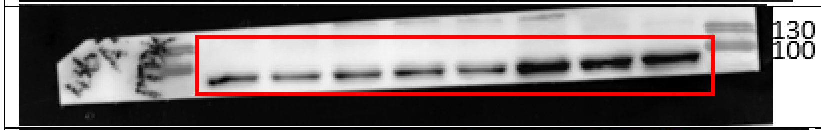	pTBK1
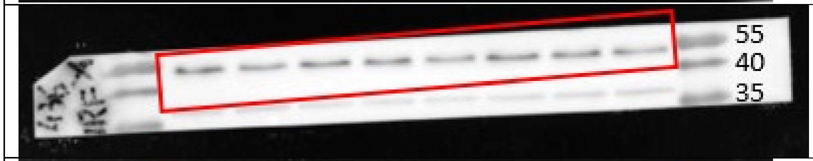	IRF3
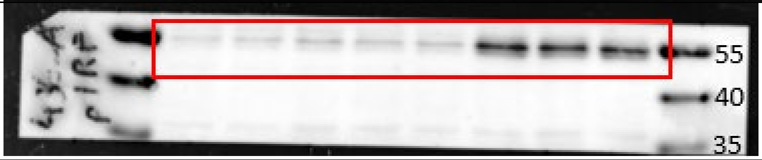	pIRF3
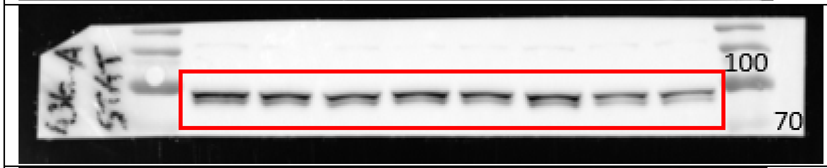	STAT1
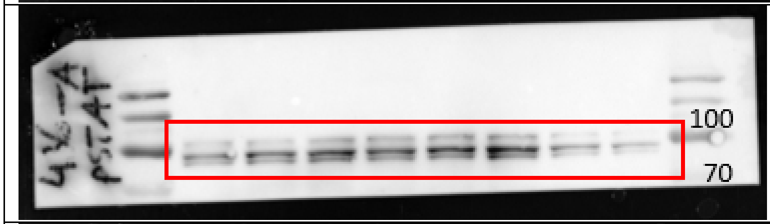	pSTAT1
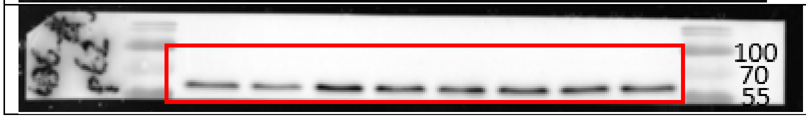	p62

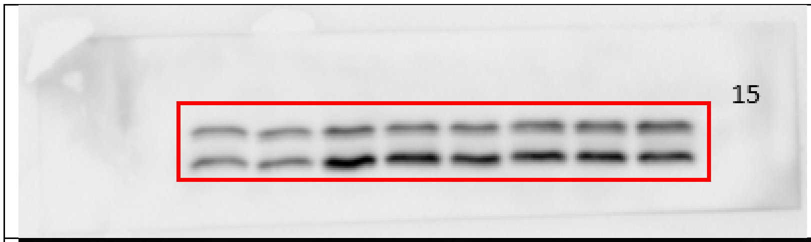	LC3
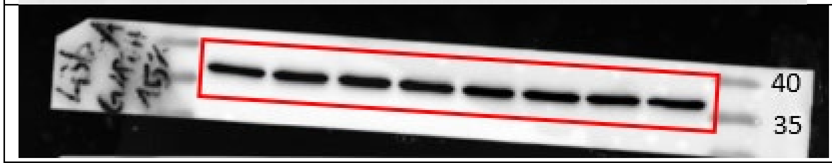	GAPDH

Supplementary Fig 2C

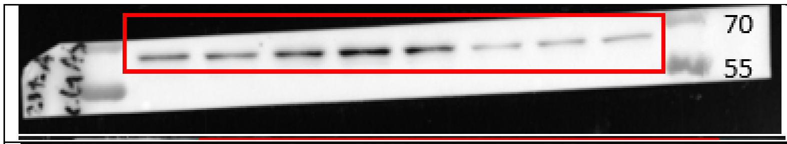	cGAS
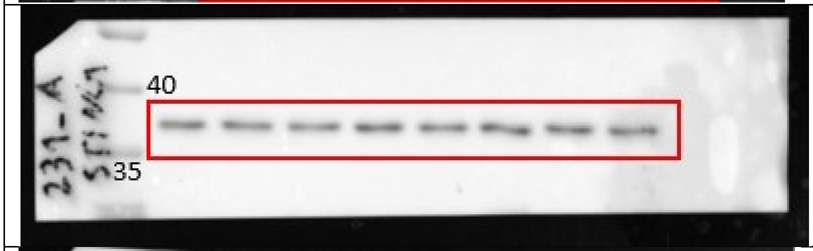	STING
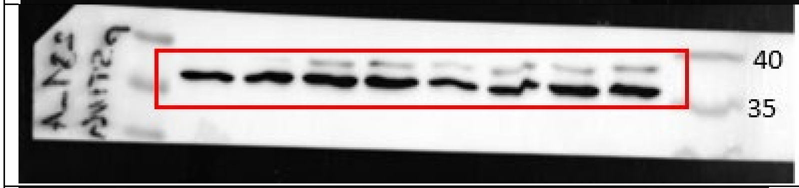	pSTING
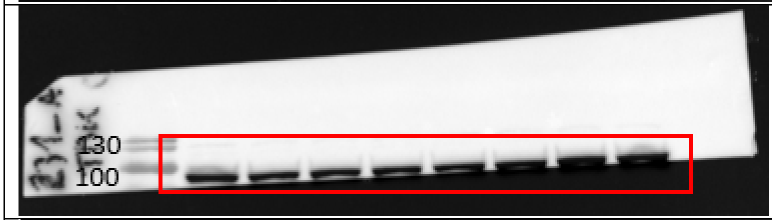	TBK1
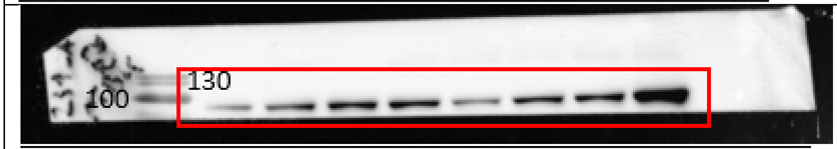	pTBK1
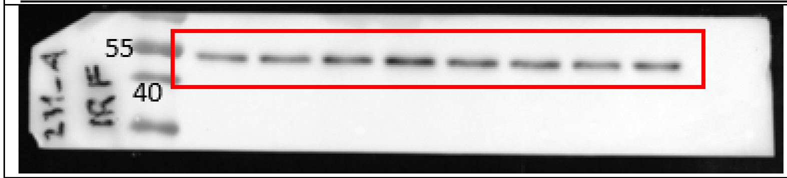	IRF3
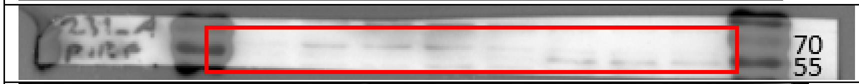	pIRF3
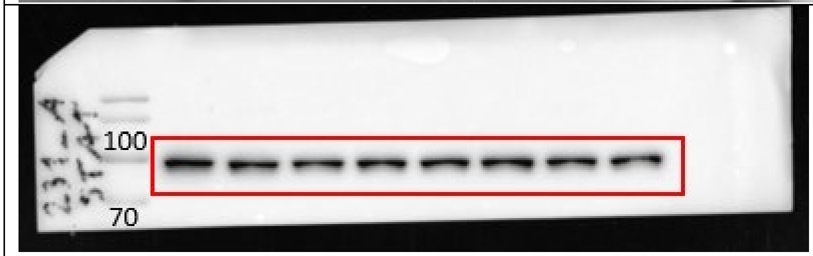	STAT1
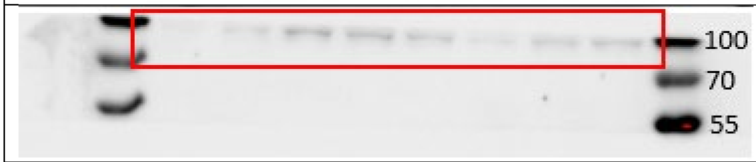	pSTAT1

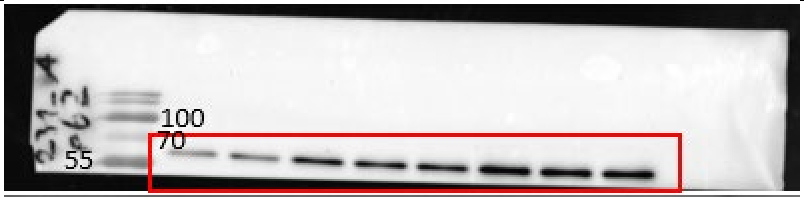	p62
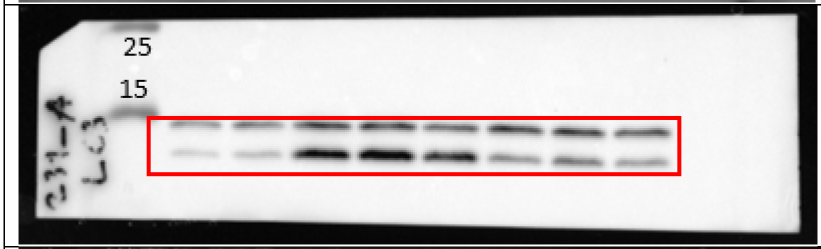	LC3
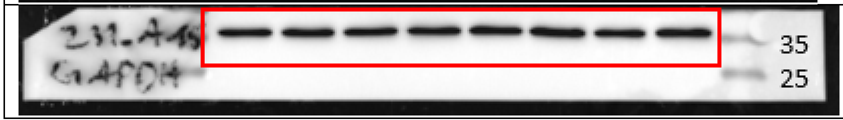	GAPDH

Supplementary Fig. 4

MDA-MB-468	
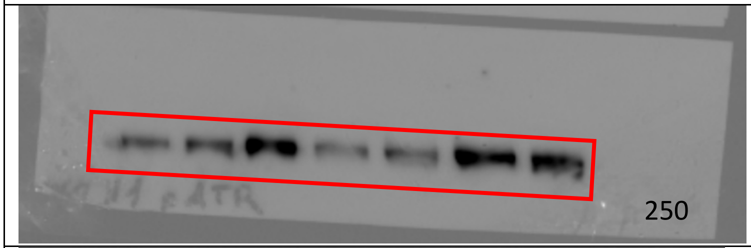	pATR
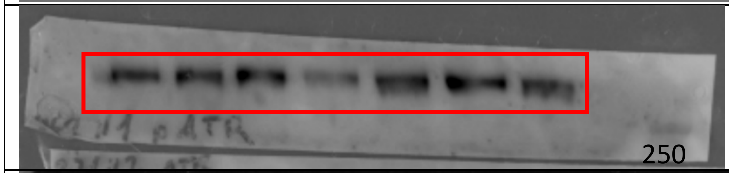	ATR
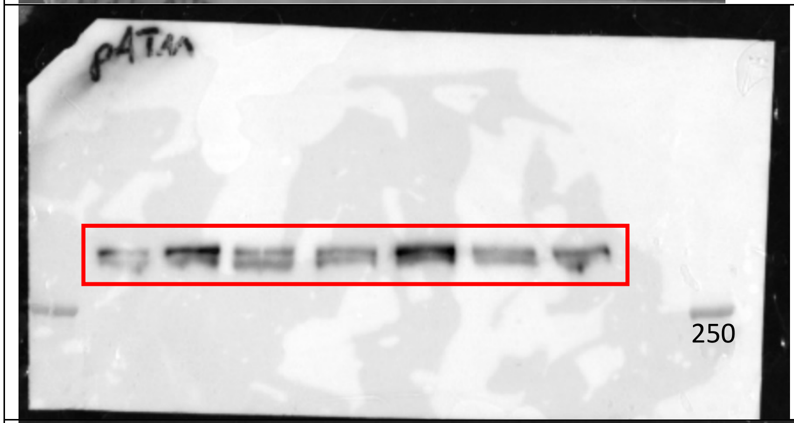	pATM
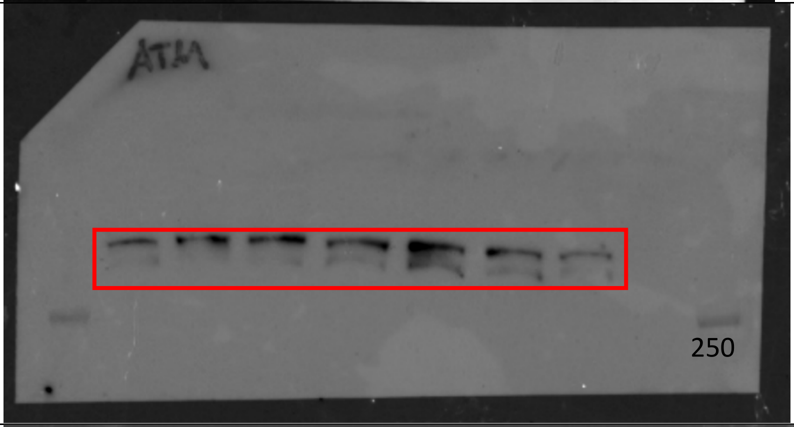	ATM
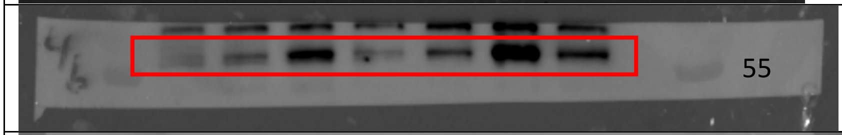	pCHK1
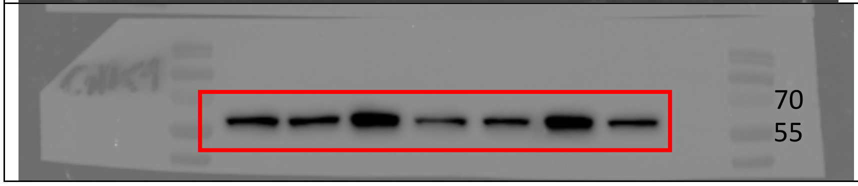	CHK1

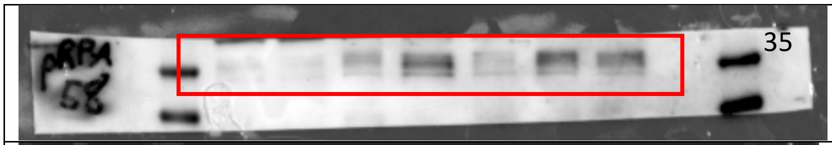	pRPA S4/S8
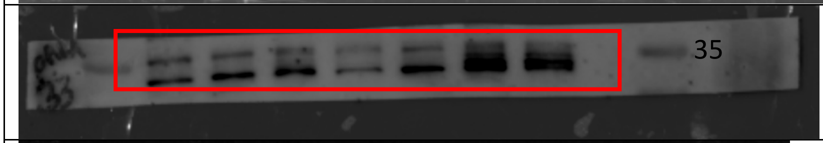	pRPA S33
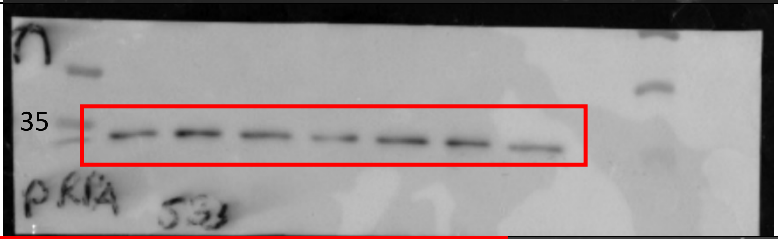	RPA
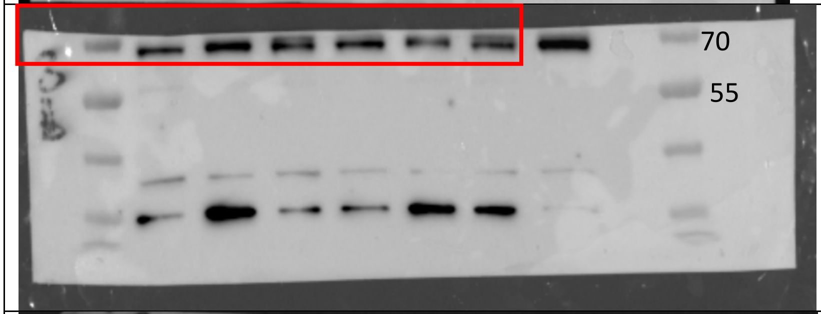	pCHK2
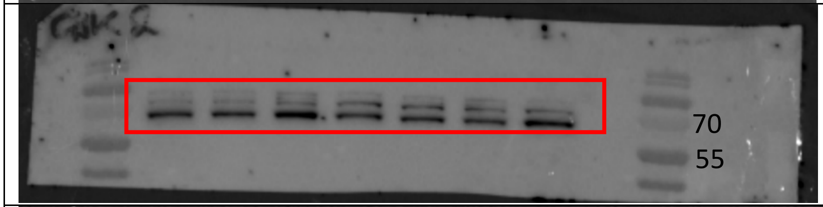	CHK2
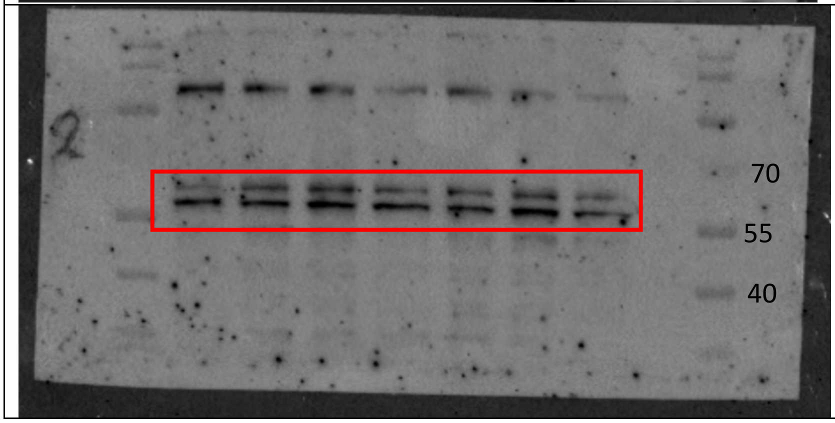	pMLKL

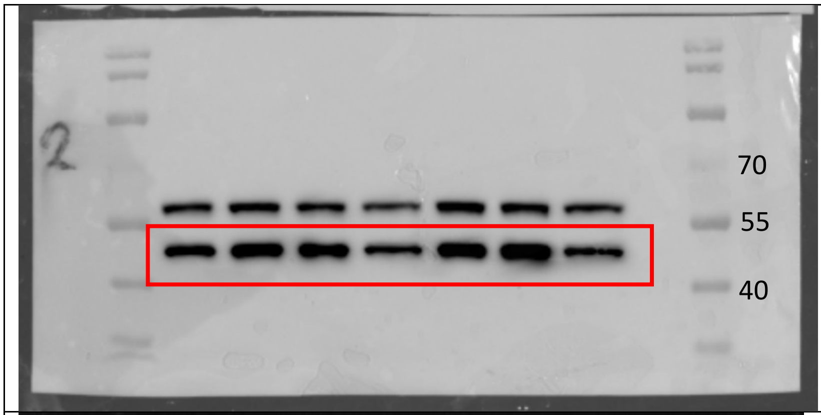	MLKL
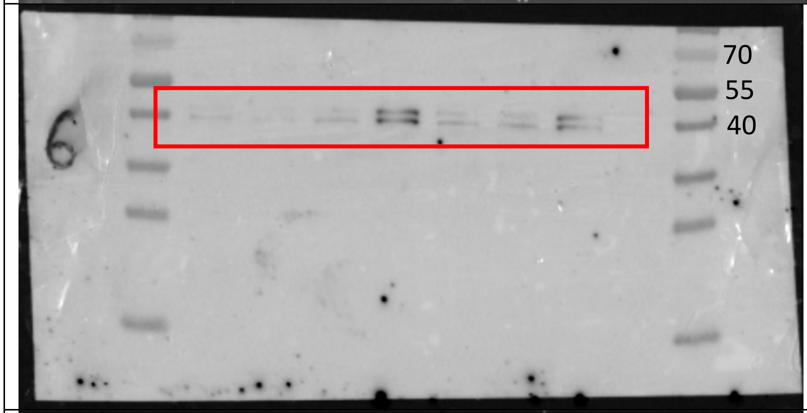	Capsase 8
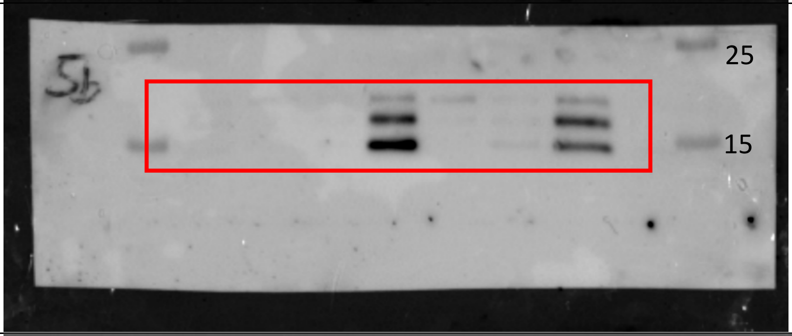	Cleaved caspase 3
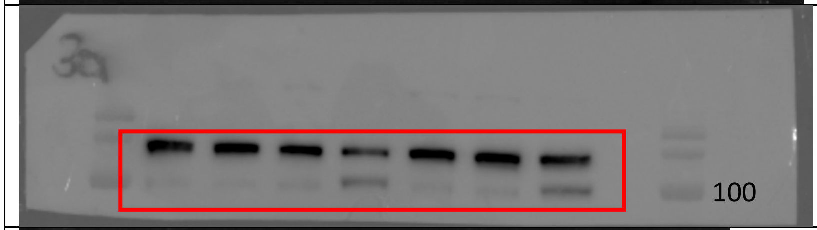	PARP1
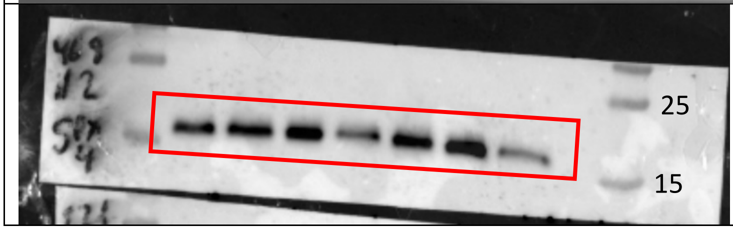	GPX4

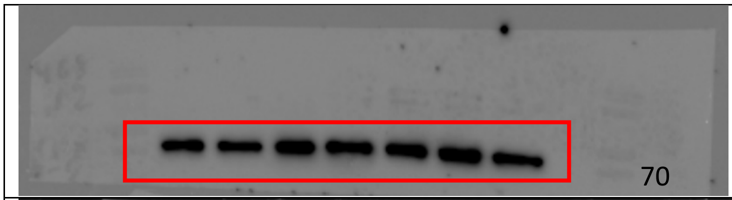	COX-2
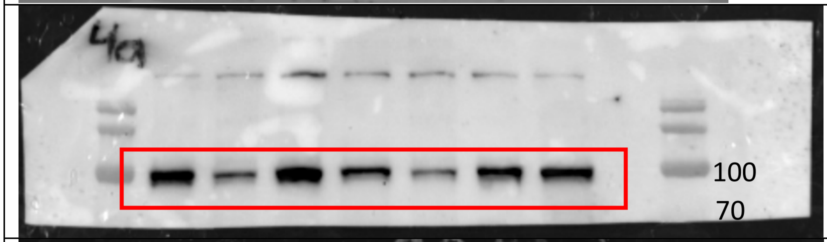	pSTAT1
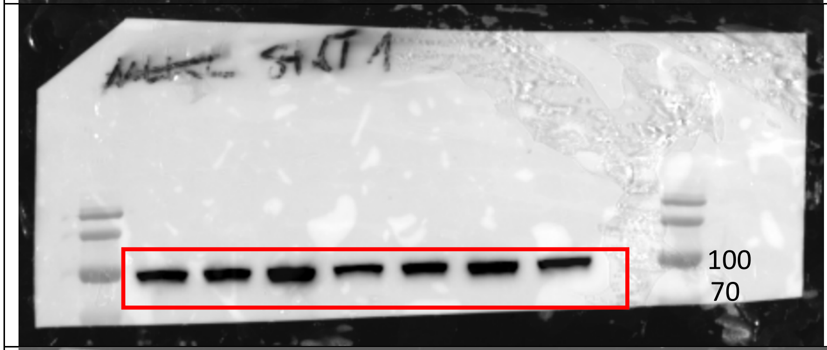	STAT1
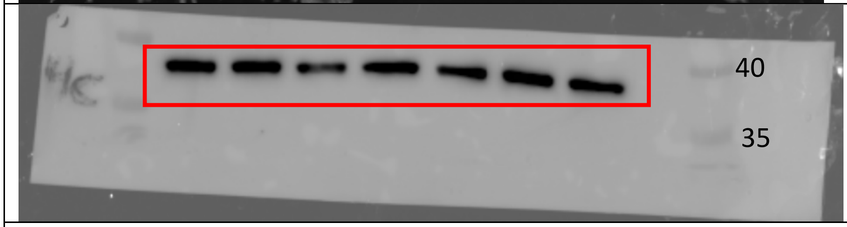	GAPDH
MDA-MB-436	
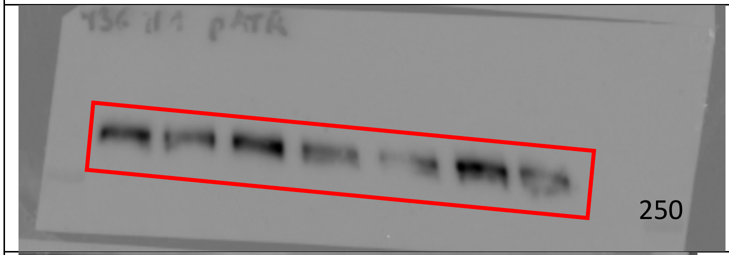	pATR
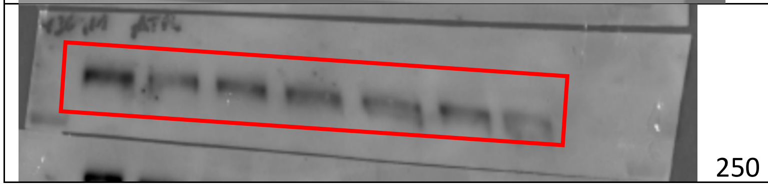	ATR

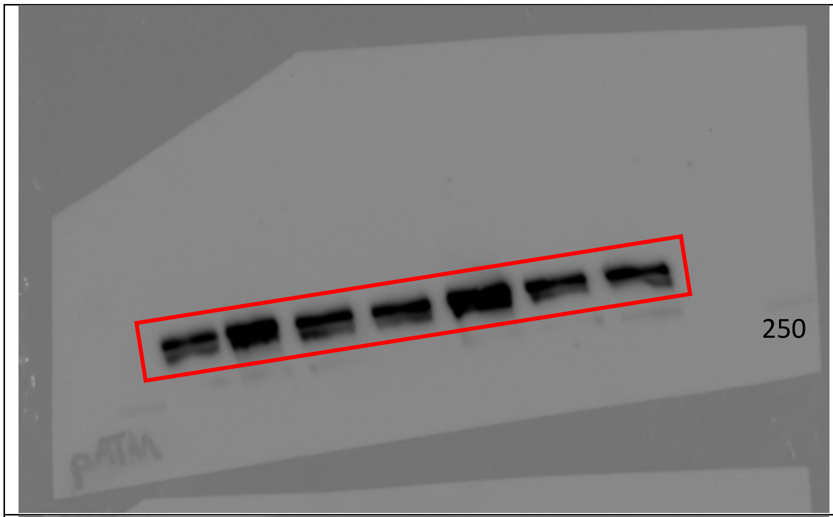	pATM
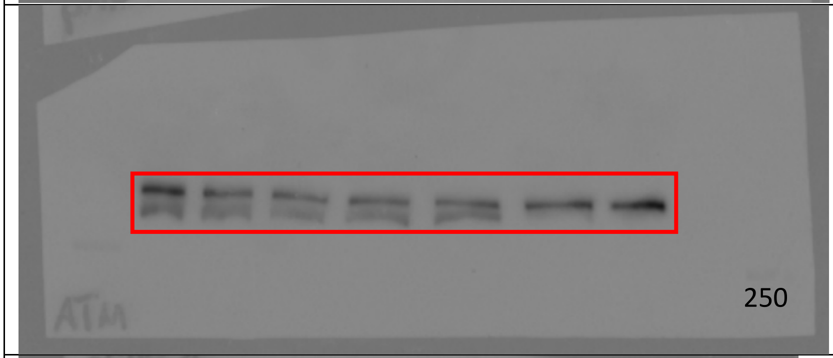	ATM
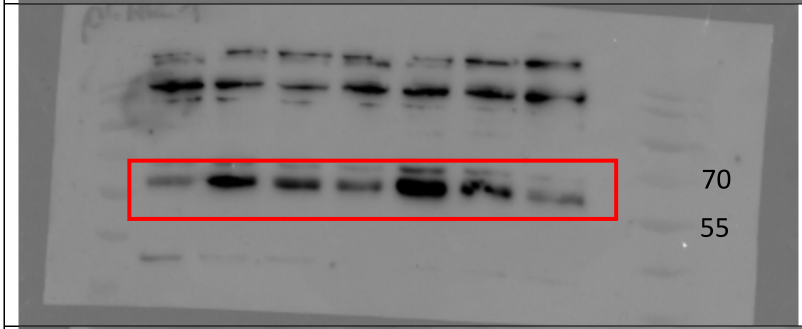	pCHK1
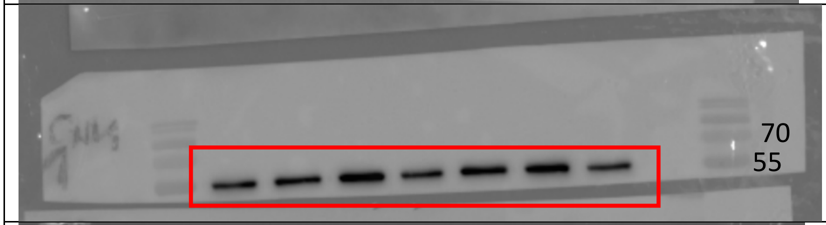	CHK1
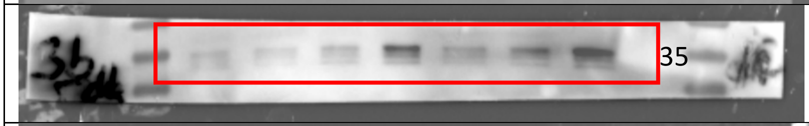	pRPA S4/S8
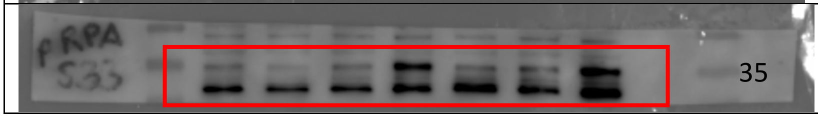	pRPA S33

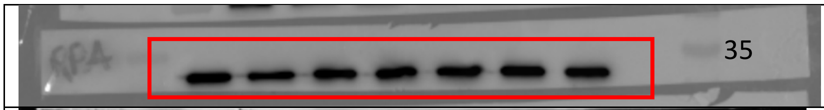	RPA
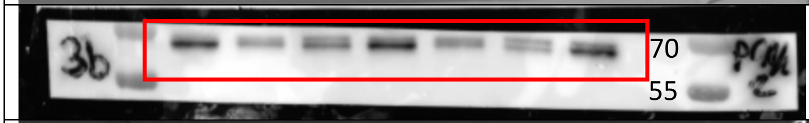	pCHK2
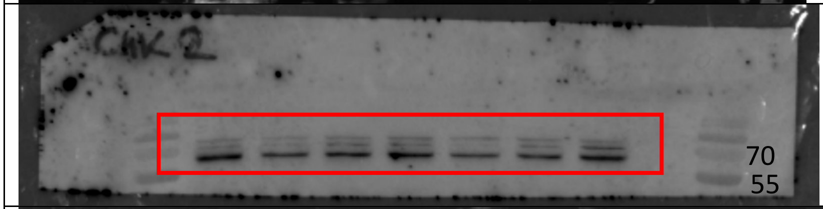	CHK2
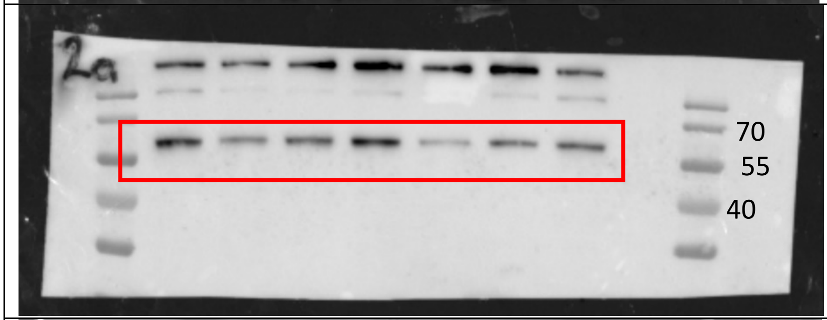	pMLKL
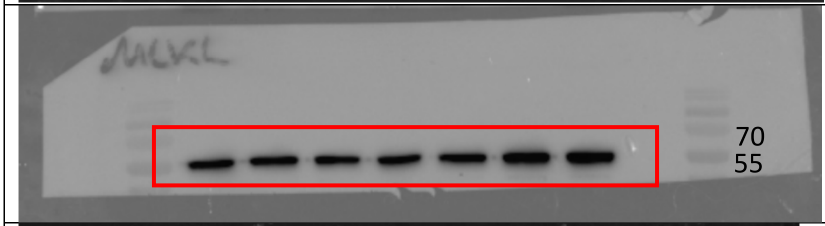	MLKL
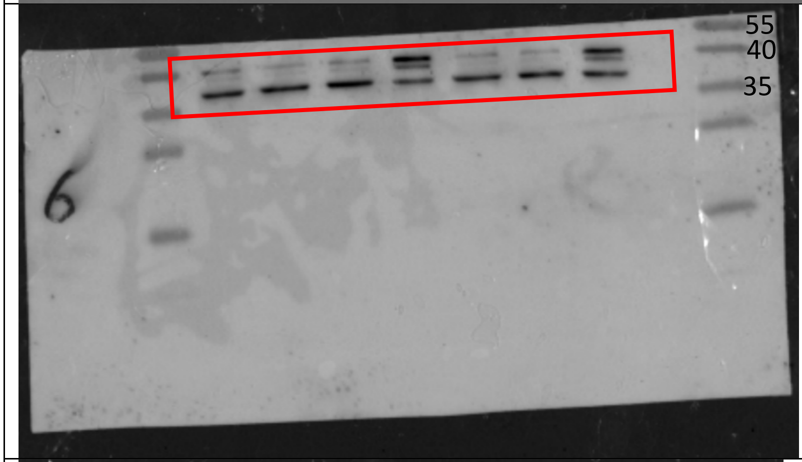	Capsase 8
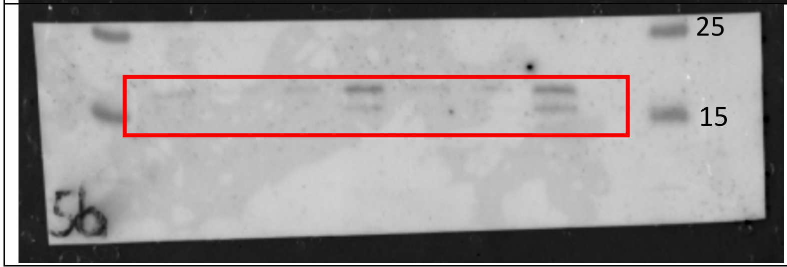	Cleaved caspase 3

<table>
<tr><td>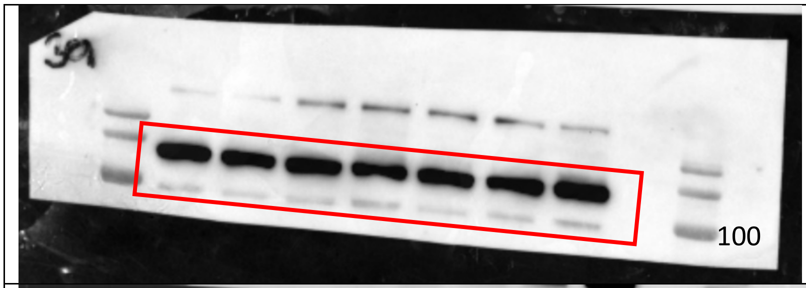
</td><td>PARP1</td></tr>
<tr><td>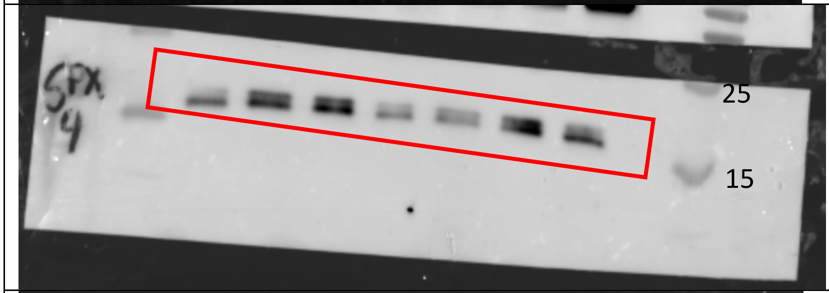
</td><td>GPX4</td></tr>
<tr><td>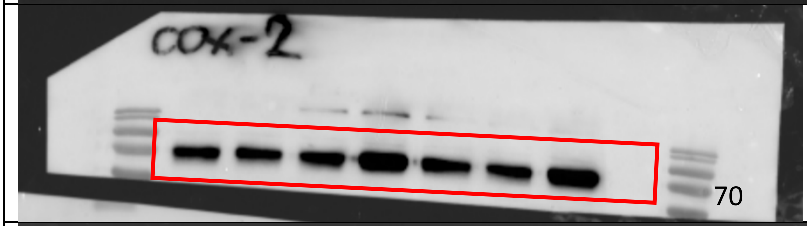
</td><td>COX-2</td></tr>
<tr><td>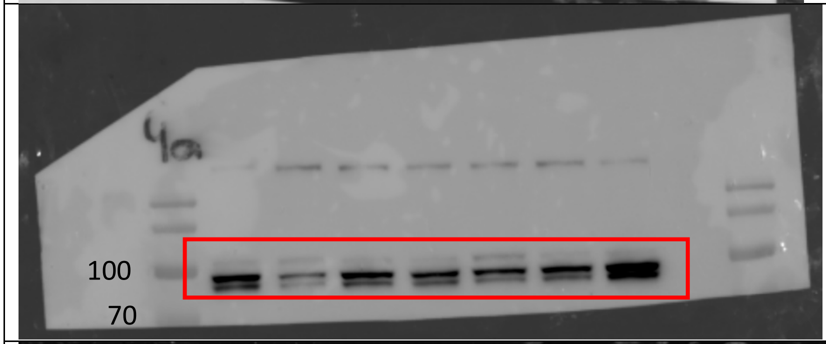
</td><td>pSTAT1</td></tr>
<tr><td>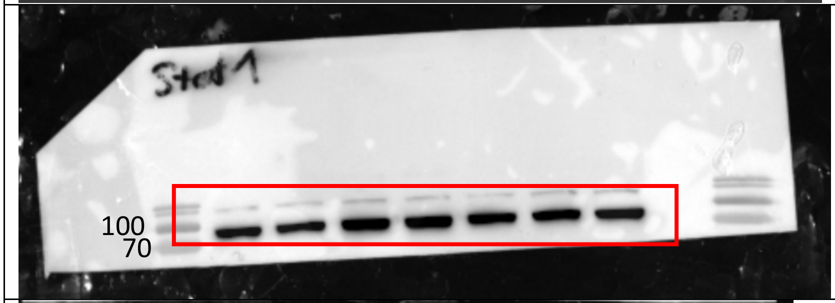
</td><td>STAT1</td></tr>
<tr><td>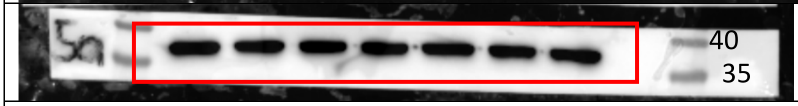
</td><td>GAPDH</td></tr>
<tr><td colspan="2">MDA-MB-231</td></tr>
</table>

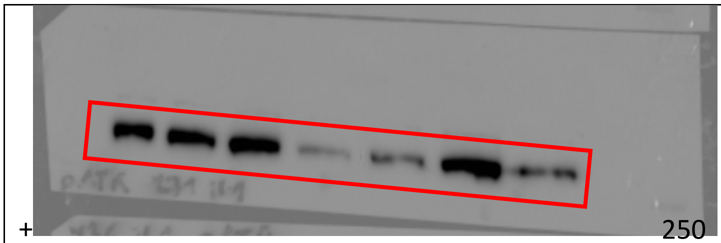	pATR
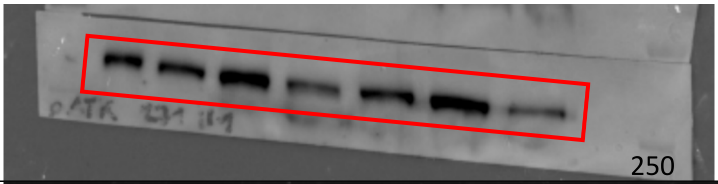	ATR
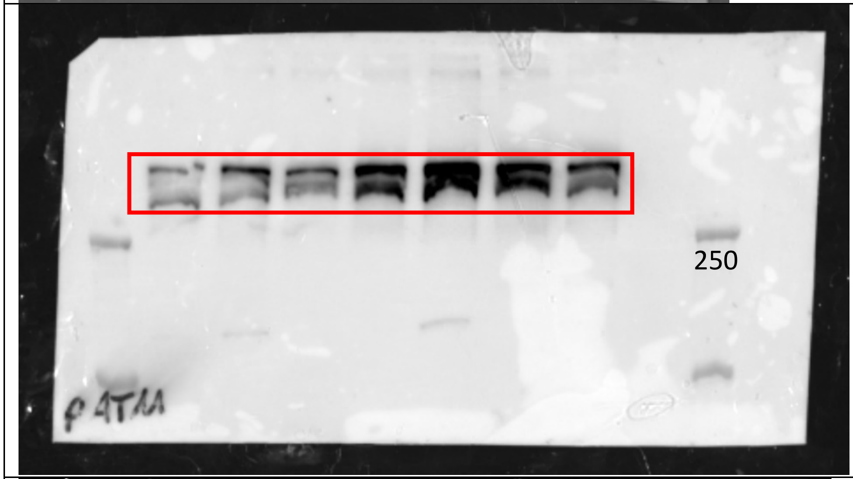	pATM
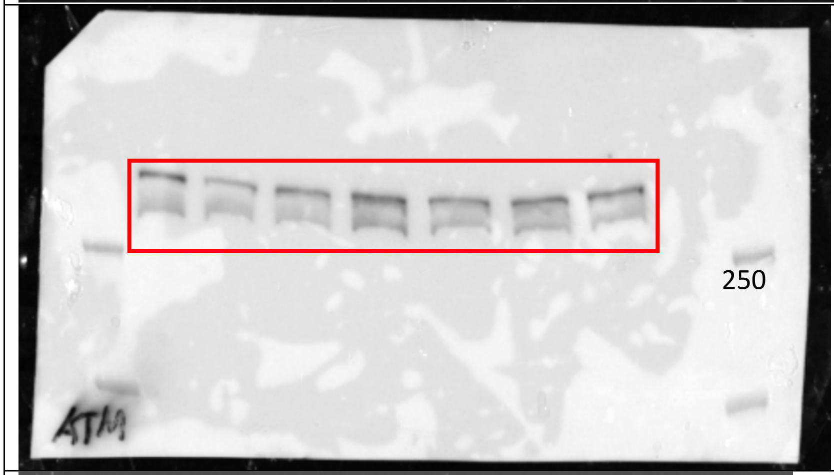	ATM
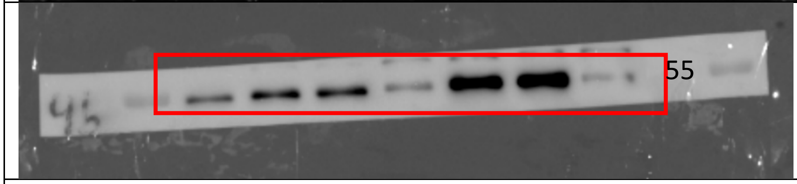	pCHK1
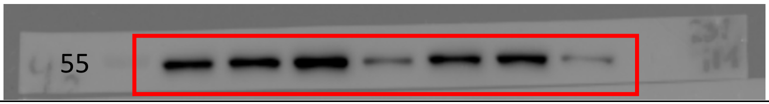	CHK1

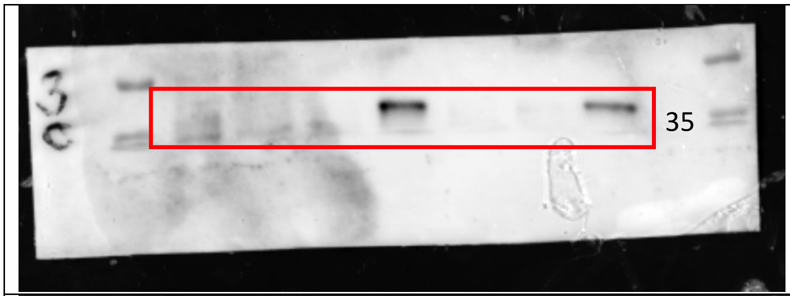	pRPA S4/S8
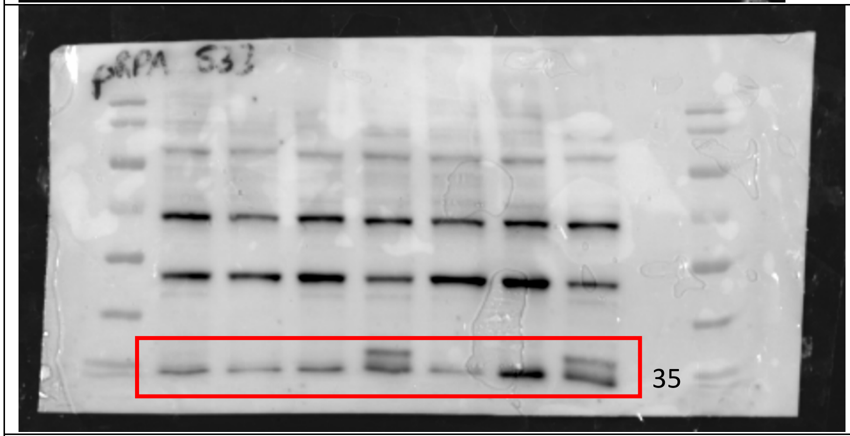	pRPA S33
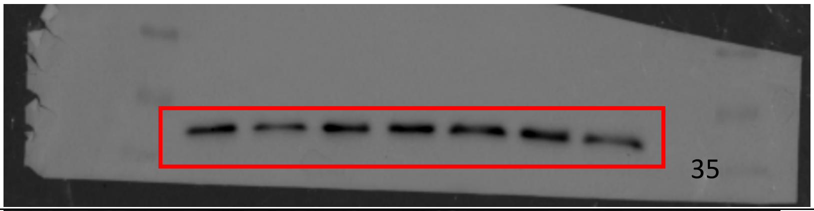	RPA
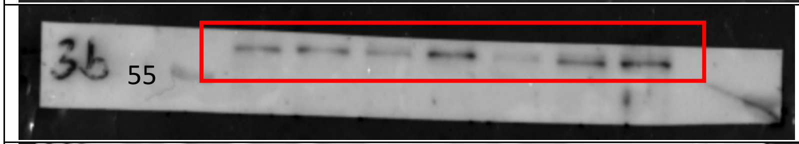	pCHK2
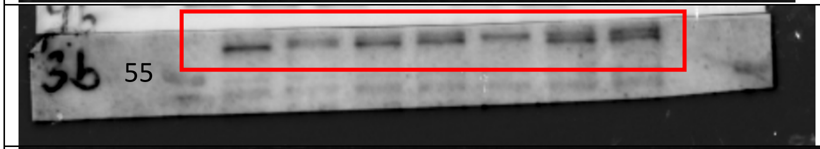	CHK2
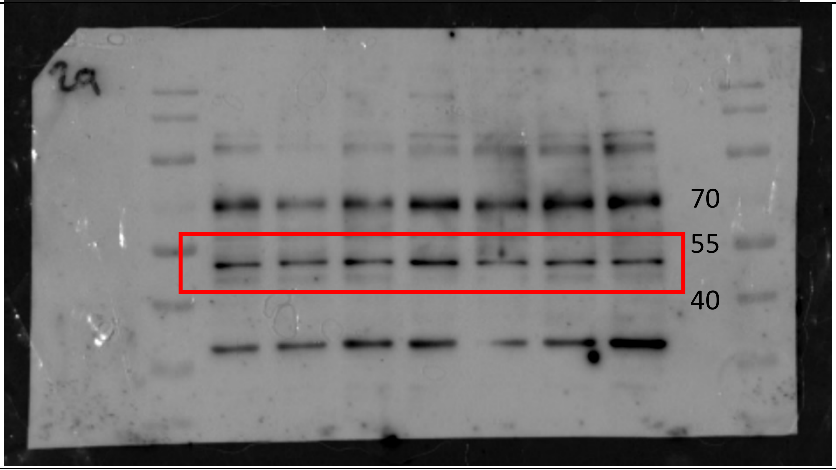	pMLKL

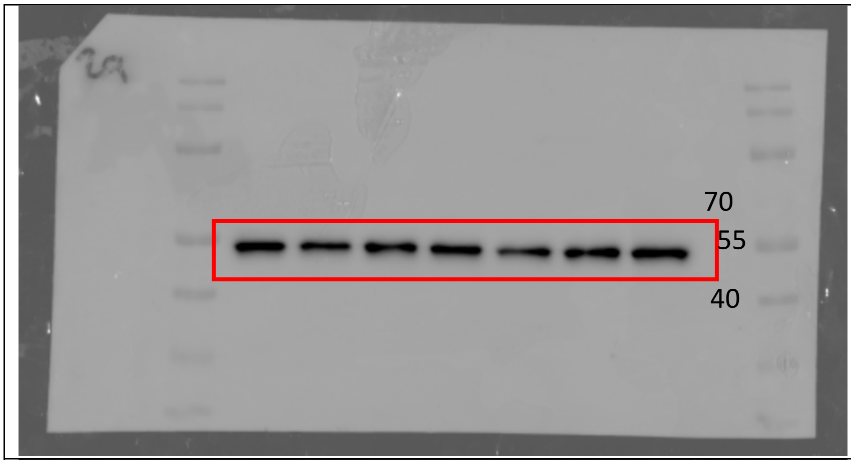	MLKL
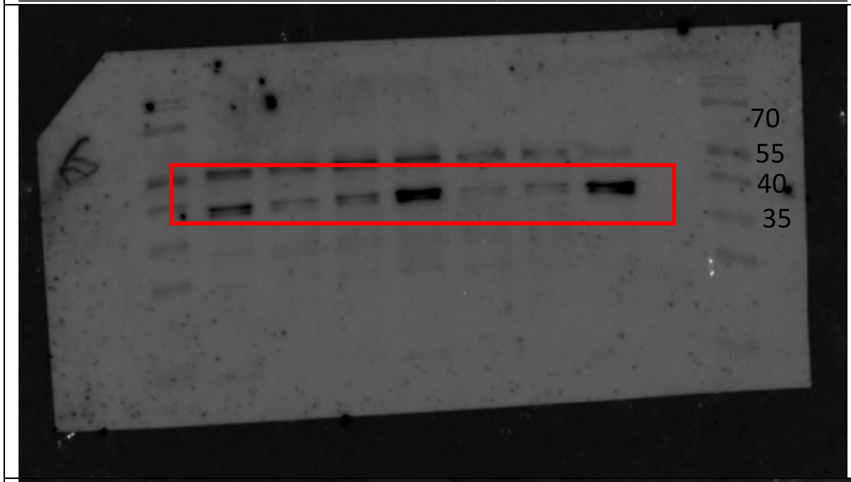	Capsase 8
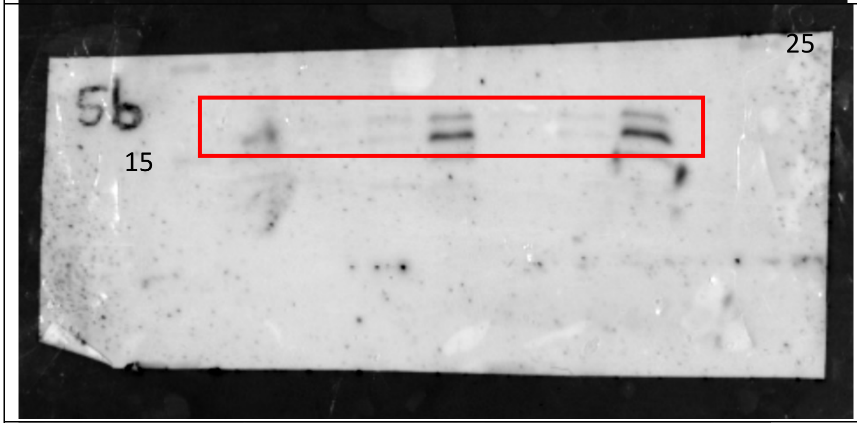	Cleaved caspase 3
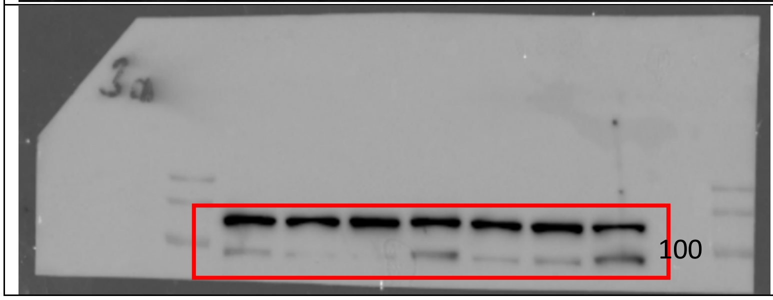	PARP1

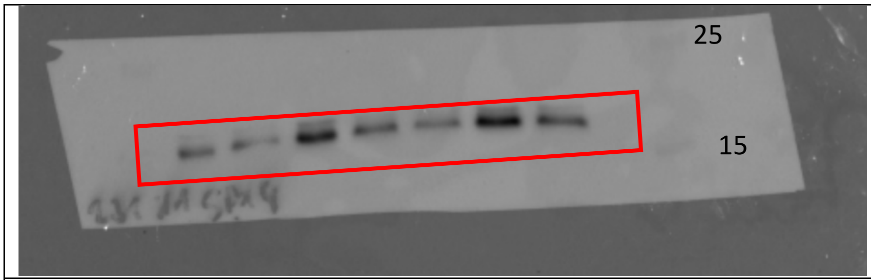	GPX4
^<br>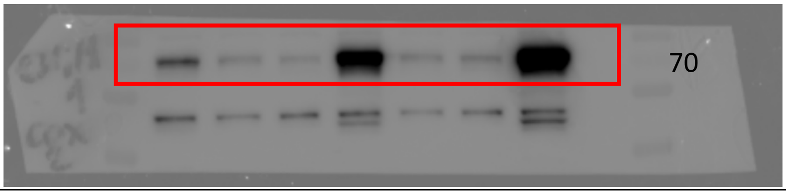	COX-2
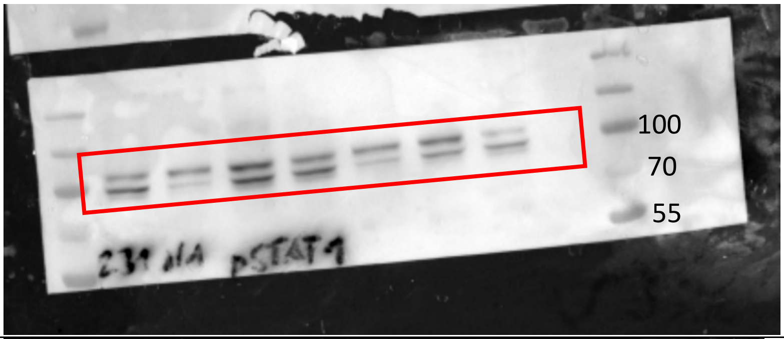	pSTAT1
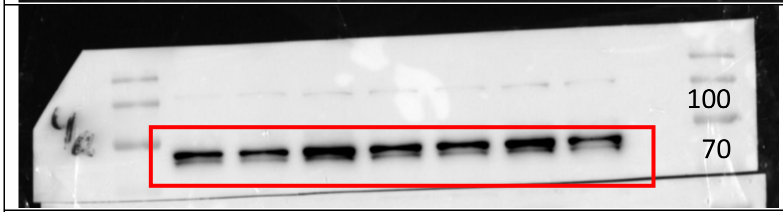	STAT1
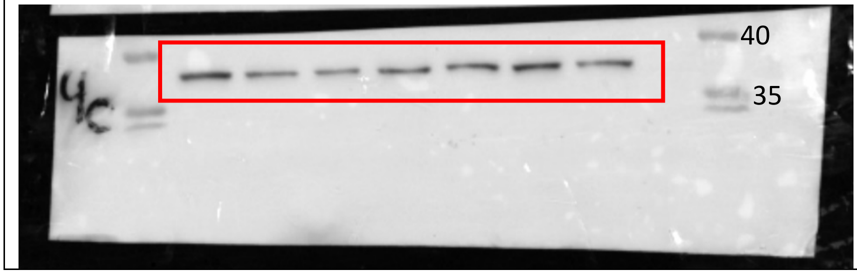	GAPDH

Supplementary Fig. 6A

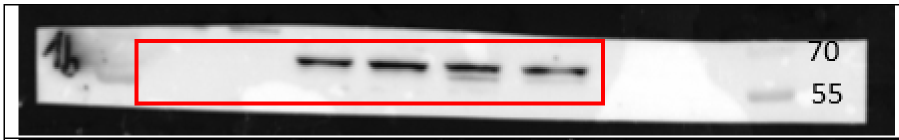	cGAS
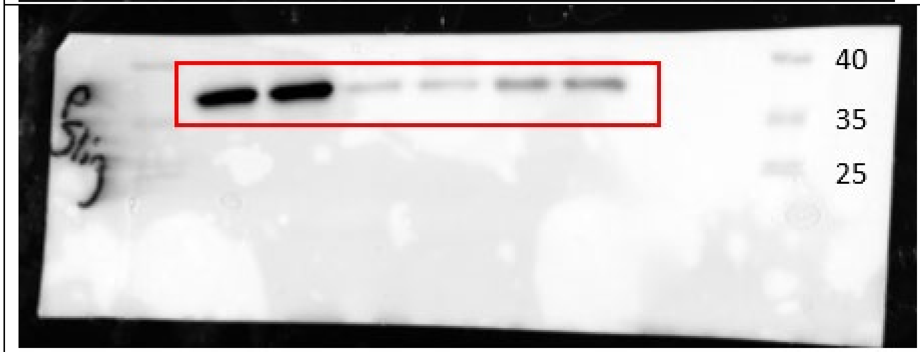	STING
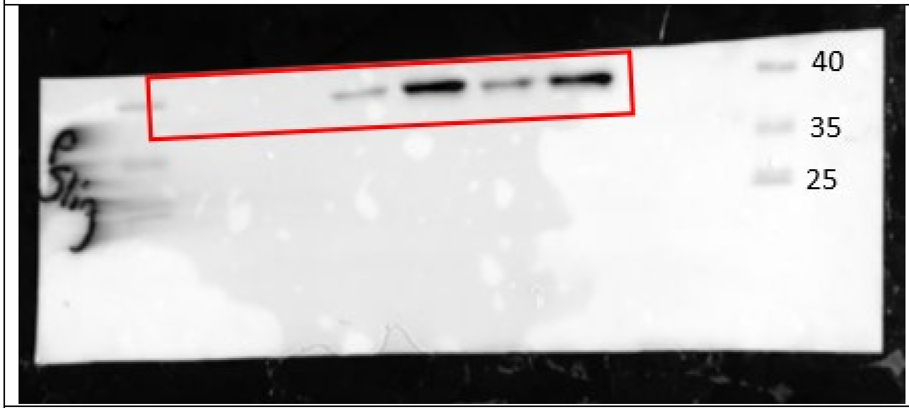	pSTING
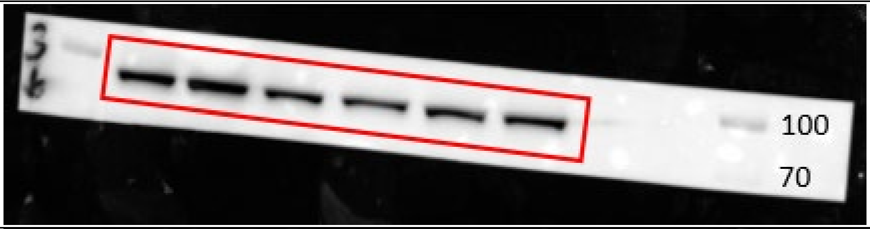	TBK1
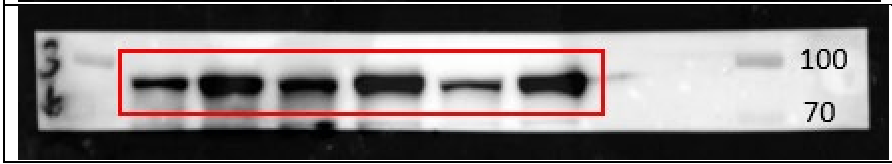	pTBK1

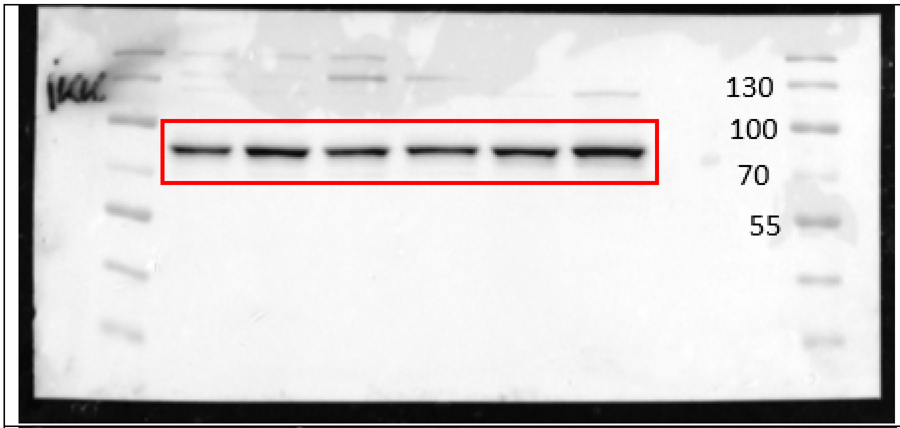	IKKϵ
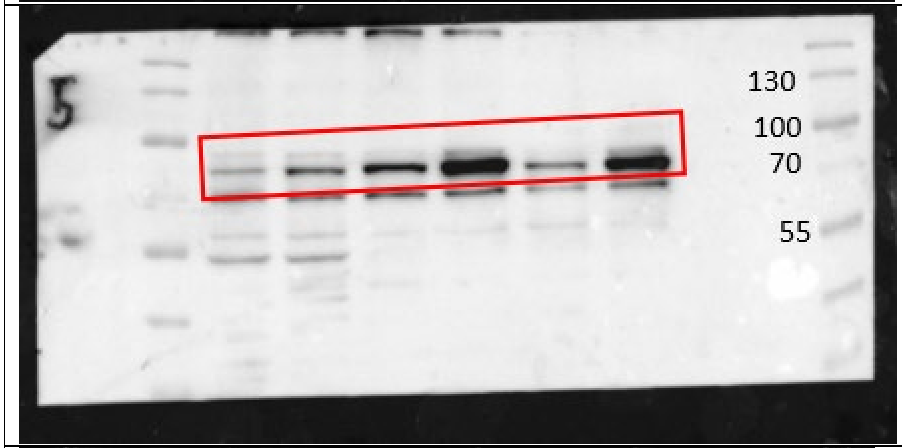	pIKKϵ
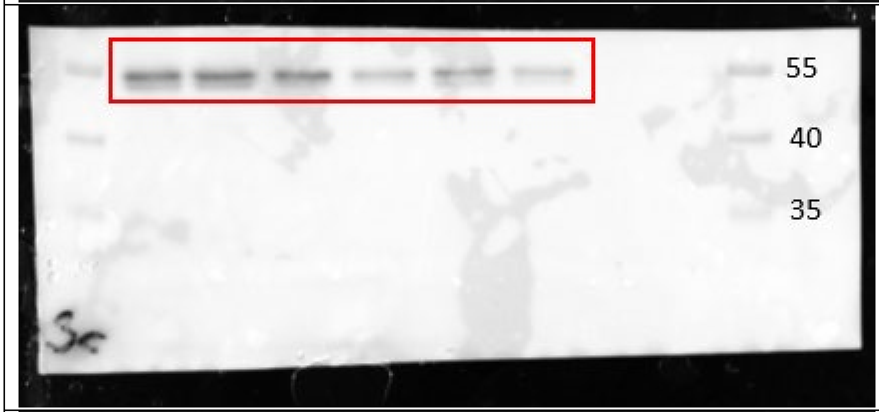	IRF3
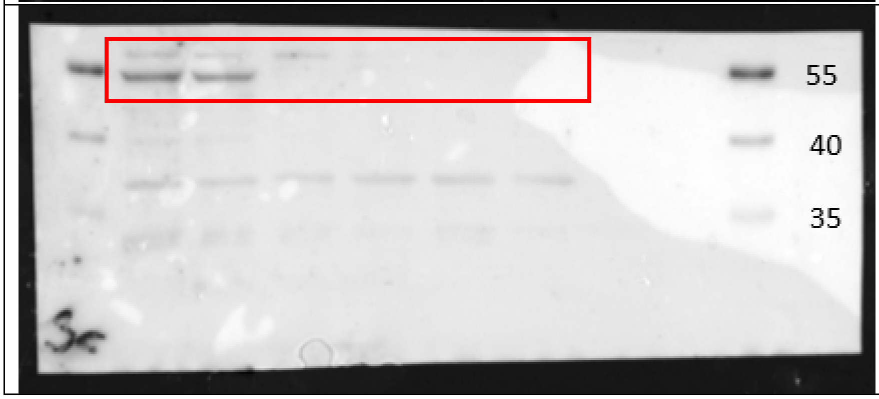	pIRF3

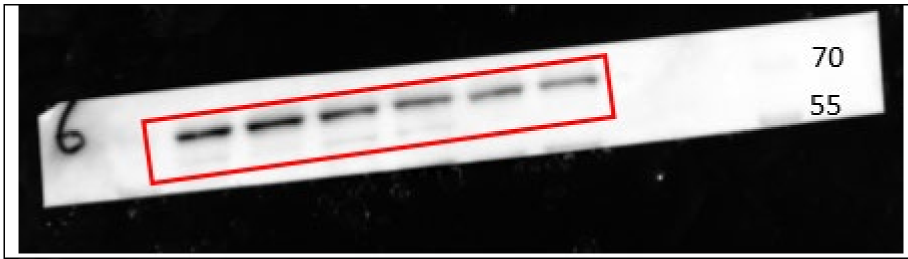	RelA
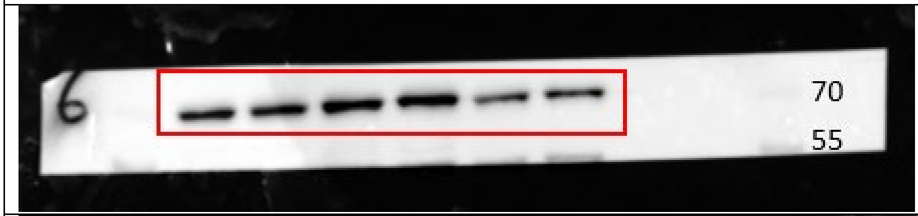	pRelA
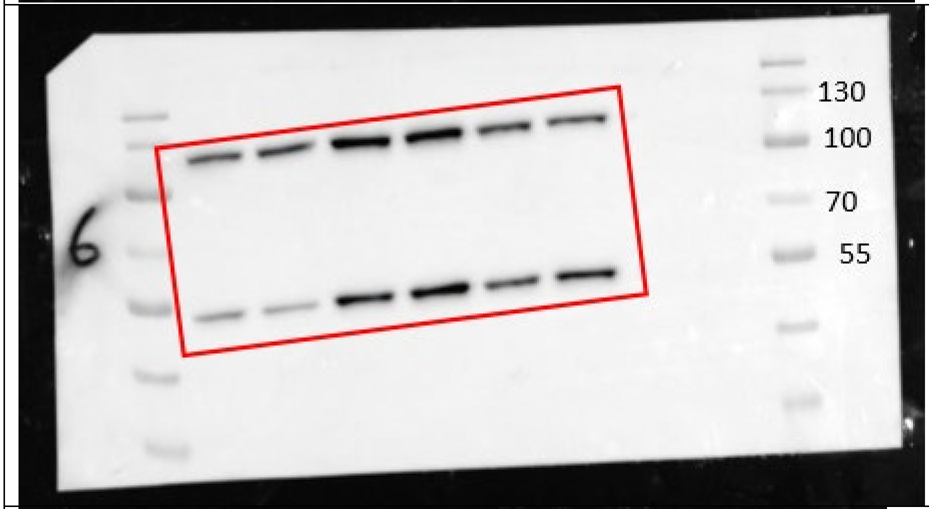	p100/p52
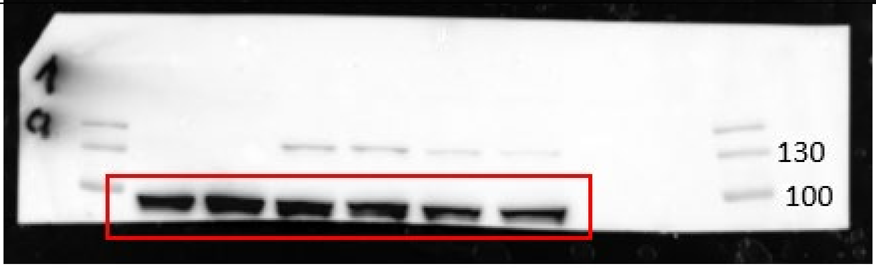	STAT1
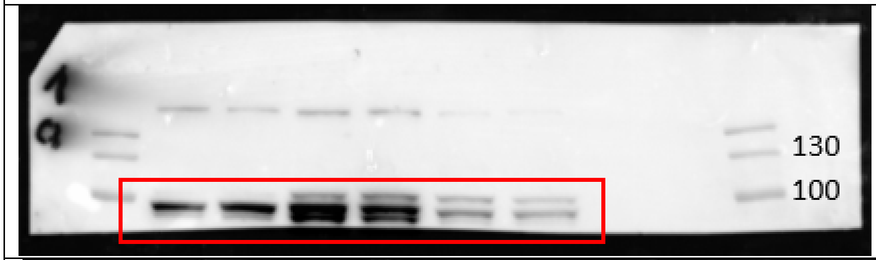	pSTAT1
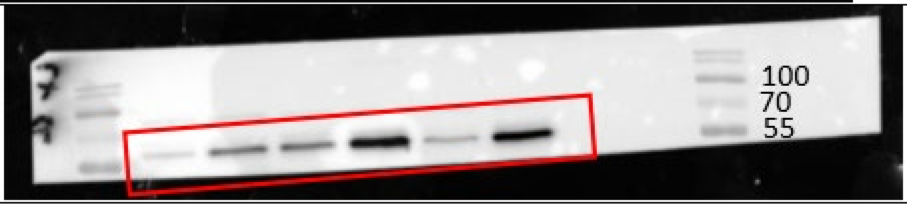	p62

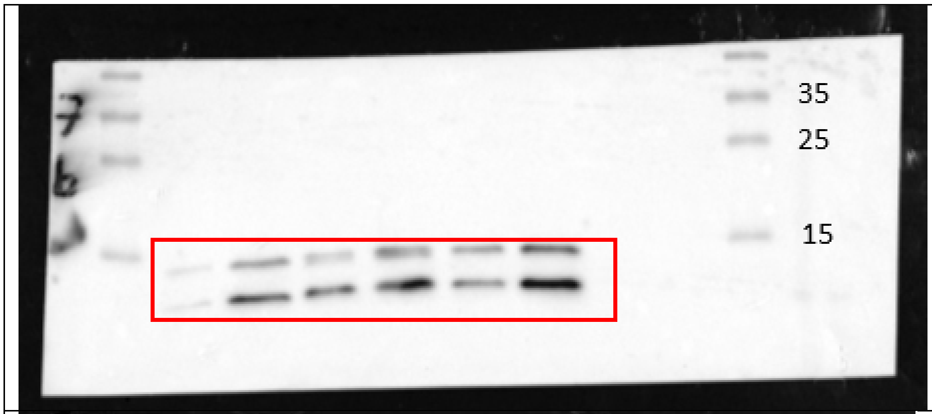	LC3
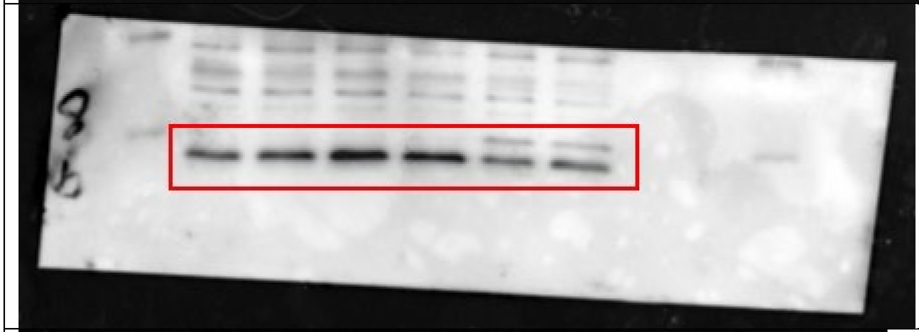	γH2AX
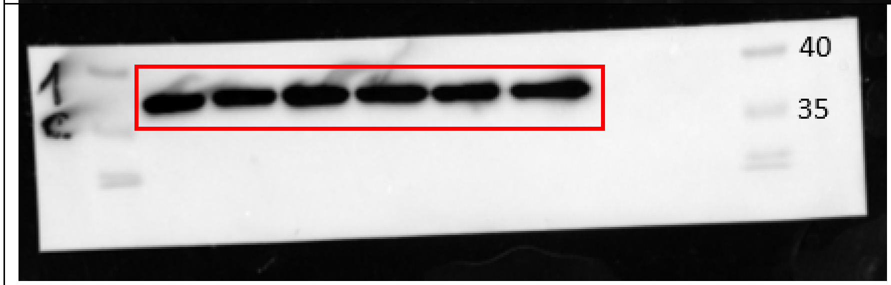	GAPDH
