# Supplementary material for: Molecular features of TNBC govern heterogeneity in the response to radiation and autophagy inhibition
Source: Cell Death Dis. 2025 Jul 21;16(1):540. doi: 10.1038/s41419-025-07873-w (PMC12280211; doi:10.1038/s41419-025-07873-w)
Supplement: Supplementary file 3 — Supplementary information [file 41419_2025_7873_MOESM3_ESM.docx]

**Supplementary Figures**


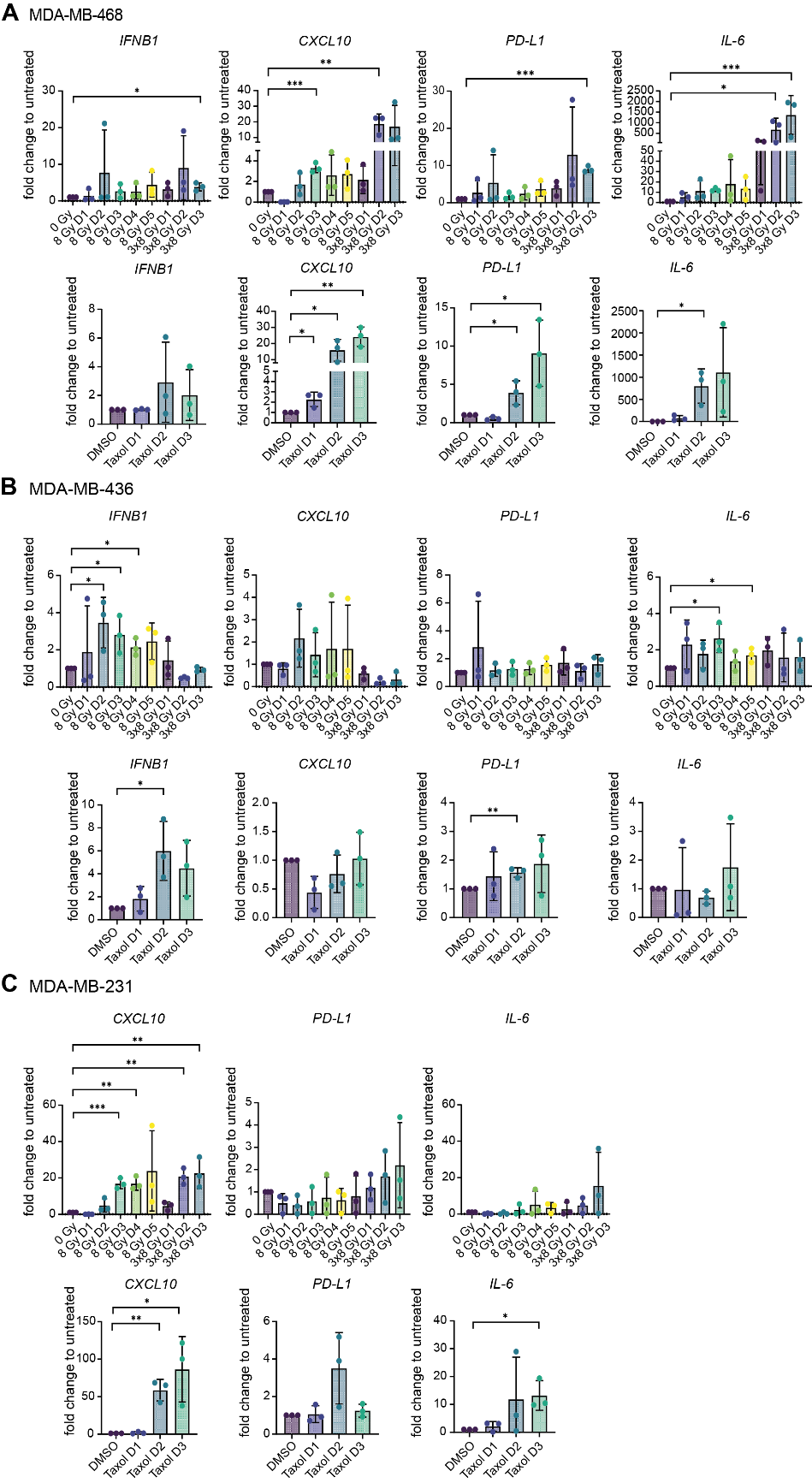


**Supplementary Fig. 1 Time course analysis of type I IFN response in MDA-MB-468, MDA-MB-436 and MDA-MB-231 treated with radiation or taxol.** Cells were irradiated with 8 Gy single dose and 3x8 Gy fractionated dose or treated with 1 μM of taxol and harvested at different timepoints (day 1-5 after 8Gy; day 1-3 after the last irradiation for 3x8Gy; day 1-3 of taxol treatment). **A-C** RT-qPCR analysis of IFNB1, CXCL10, PD-L1 and IL-6 mRNA levels in **A** MDA-MB-468, **B** MDA-MB-436 and **C** MDA-MB-231. IFNB1 was not detectable in MDA-MB-231. TBP served as a housekeeping gene. The bars and error bars represent mean values ± standard deviation. Three biologically independent experiments were performed. P-values were calculated with an unpaired t-test. A p-value of 0.05 was considered as statistically significant.


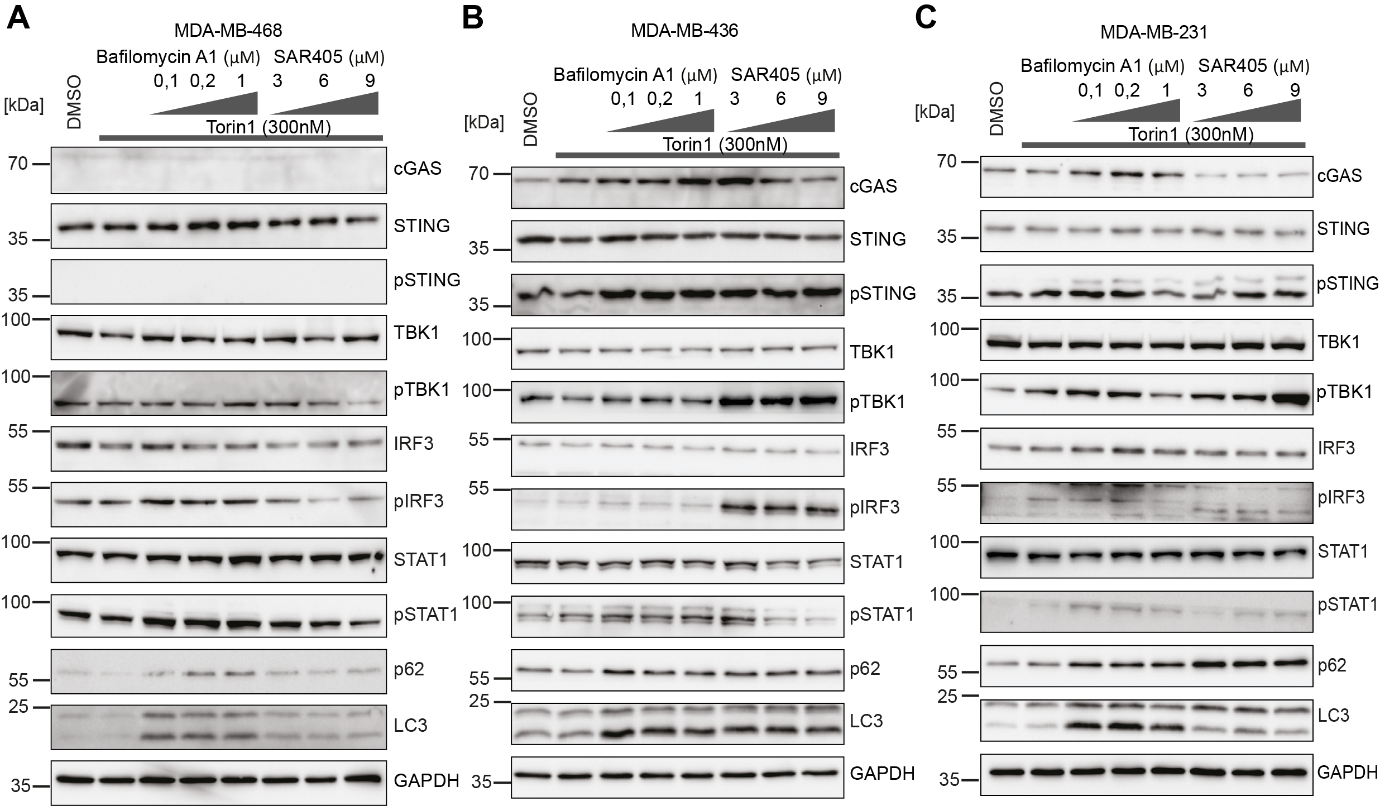


**Supplementary Fig. 2 Analysis of cGAS-STING signaling in MDA-MB-468, MDA-MB-436 and MDA-MB-231 treated with different concentrations of autophagy inhibitors bafilomycin A1 and SAR405. A-C** Immunoblots of **A** MDA-MB-468, **B** MDA-MB-436 and **C** MDA-MB-231 cells treated for 8 h and harvested immediately afterwards. Treatment regimens are indicated in the respective graphs. GAPDH served as a loading control. Two biologically independent experiments were performed.


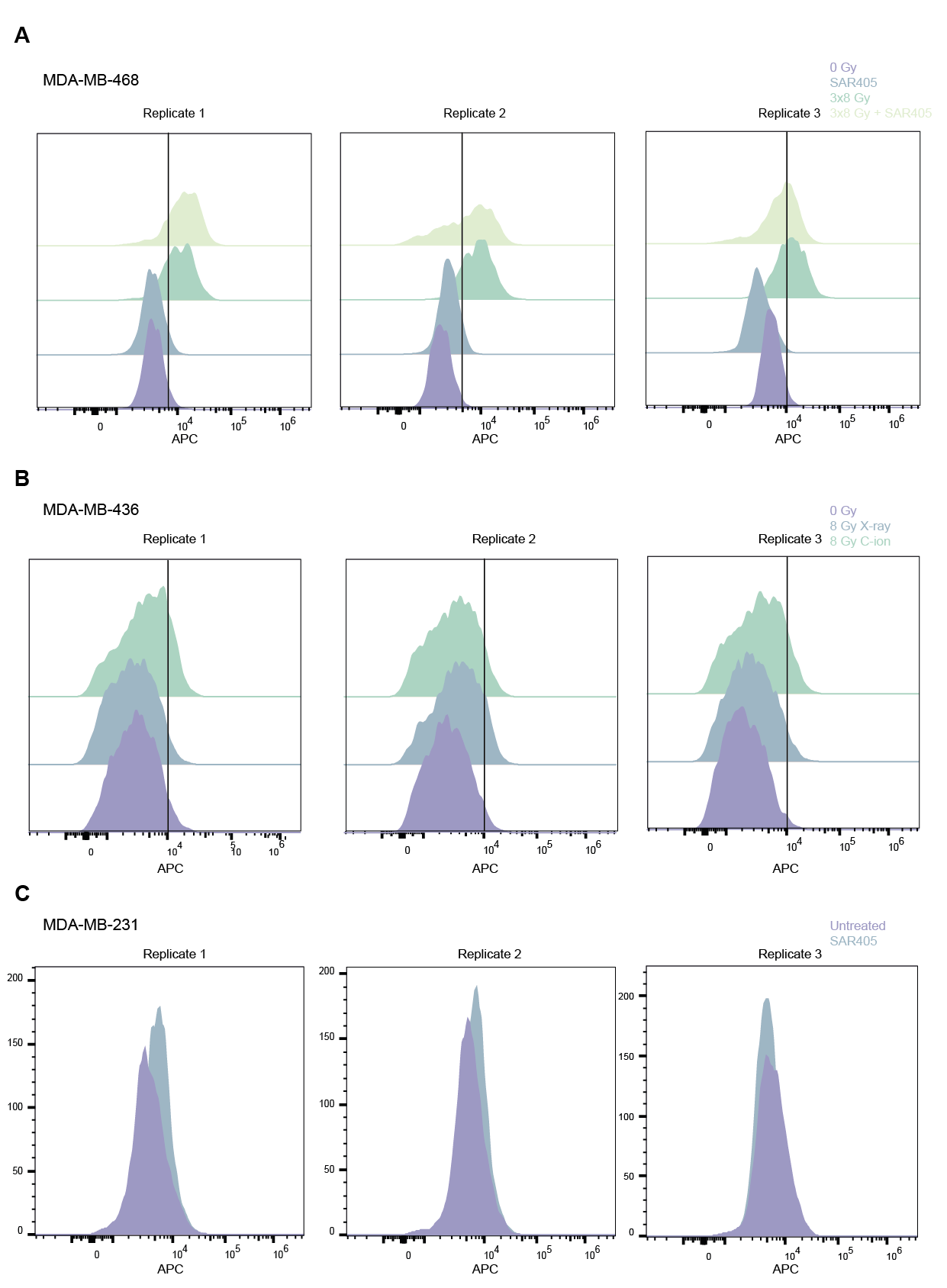


**Supplementary Fig. 3 Flow cytometry analysis of nuclear IRF3 in TNBC cell lines.** To quantify IRF3 nuclear translocation, nuclear proteins were extracted, immunostained with APC-IRF3 antibody and analysed by flow cytometry in **A** MDA-MB-468 control cells and after treatment with SAR405, 3x8 Gy and 3x8 Gy combined with SAR405, **B** MDA-MB-436 control cells and after exposure to 8 Gy X-ray or C-ions, and **C** MDA-MB-231 control cells or treated with SAR405. Cells were harvested 72 h after radiation and exposed to 3 μM SAR405 for 72 h. The solid lines in panel A and B represent the gate to determine the percentage of nuclear IRF3-stained cells. Each gate was adjusted based on 0 Gy (untreated) sample. Flow cytometry measurements were analysed with FlowJo_v10.8.1.

**
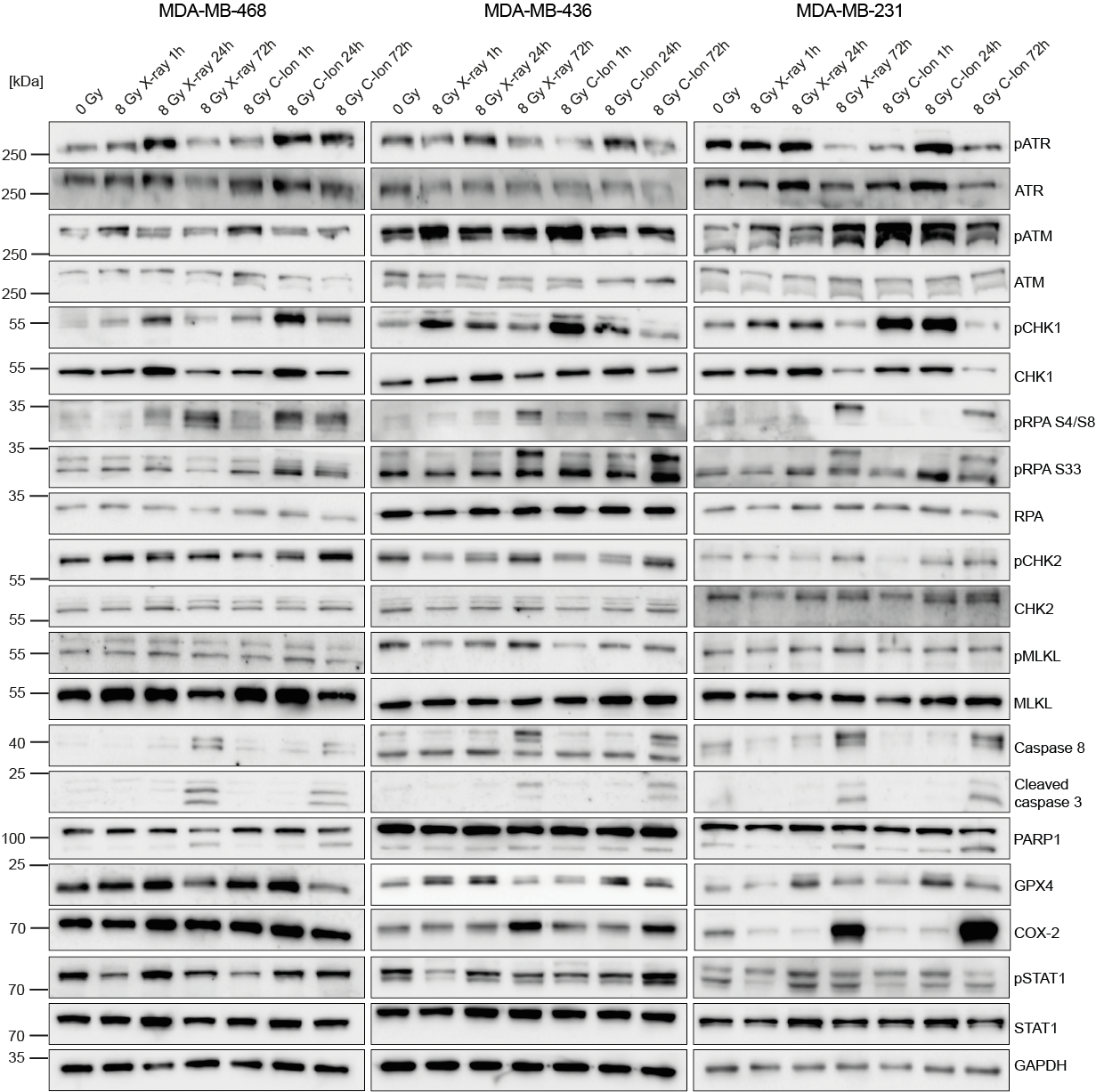
**

**Supplementary Fig. 4 DNA damage signaling in TNBC cell lines exposed to X-ray or C-ion radiation.** Immunoblots of MDA-MB-468, MDA-MB-436 and MDA-MB-231 control cells and cells treated with 8 Gy X-ray of C-ion radiation and harvested after 1 h, 24 h and 72 h. GAPDH was used as a loading control. Two biologically independent experiments were performed.


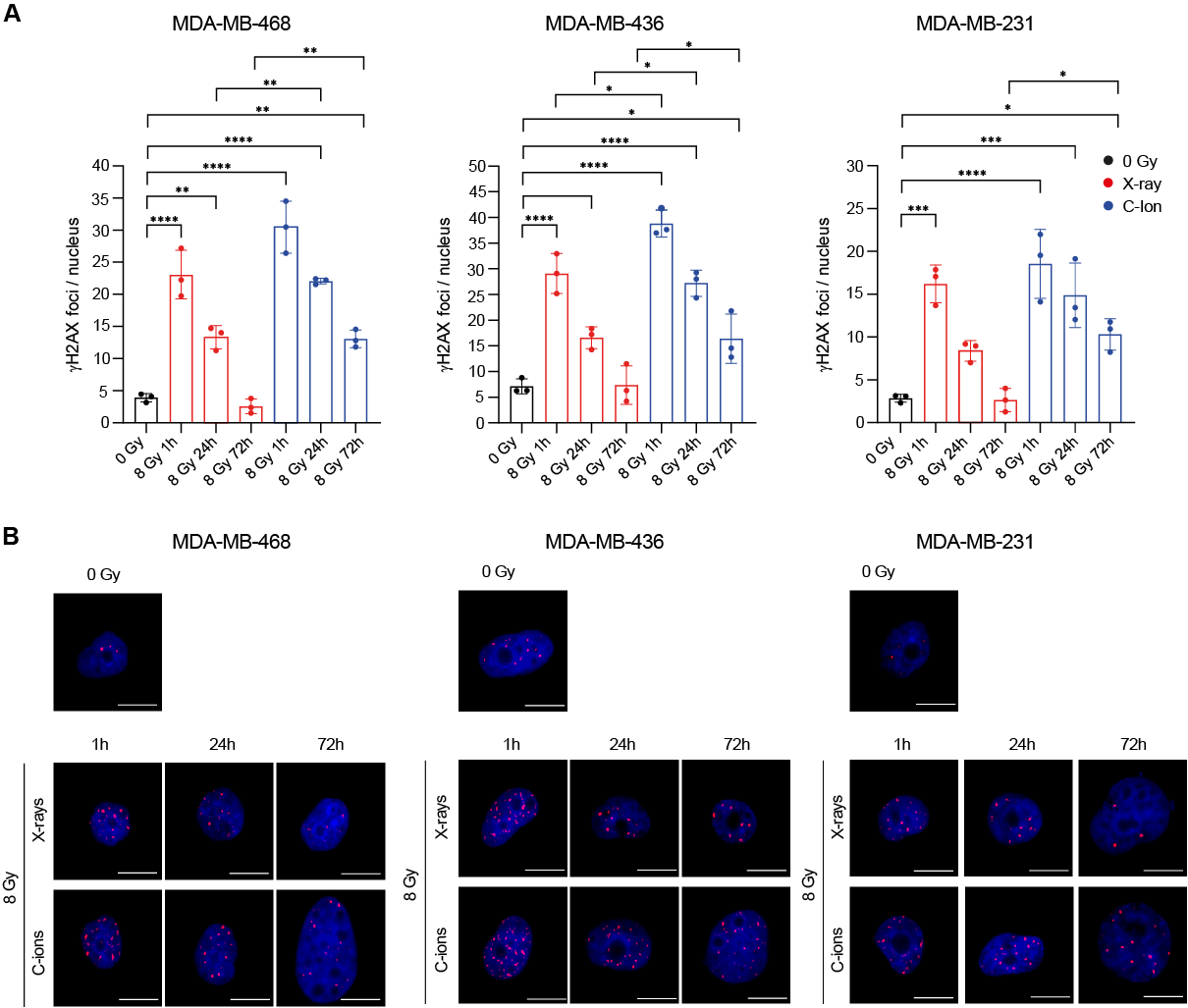


**Supplementary Fig. 5 γH2AX foci formation in TNBC cell lines exposed to X-ray or C-ion radiation.** **A** Quantification of γH2AX foci and **B** representative images of MDA-MB-468, MDA-MB-436 and MDA-MB-231 control cells and cells exposed to 8 Gy X-ray or C-ion irradiation, harvested after 1 h, 24 h and 72 h, and stained with anti-γH2AX antibody (red) and nucleus-stained with DAPI (blue). Scale bar: 10 μm. The bars and error bars represent mean values ± standard deviation. Three biologically independent experiments were performed. Statistical significance was assessed using one-way ANOVA with Tukey’s post-hoc test (*≤ 0.05; **≤ 0.01; ***≤ 0.005, ****≤0.0001).


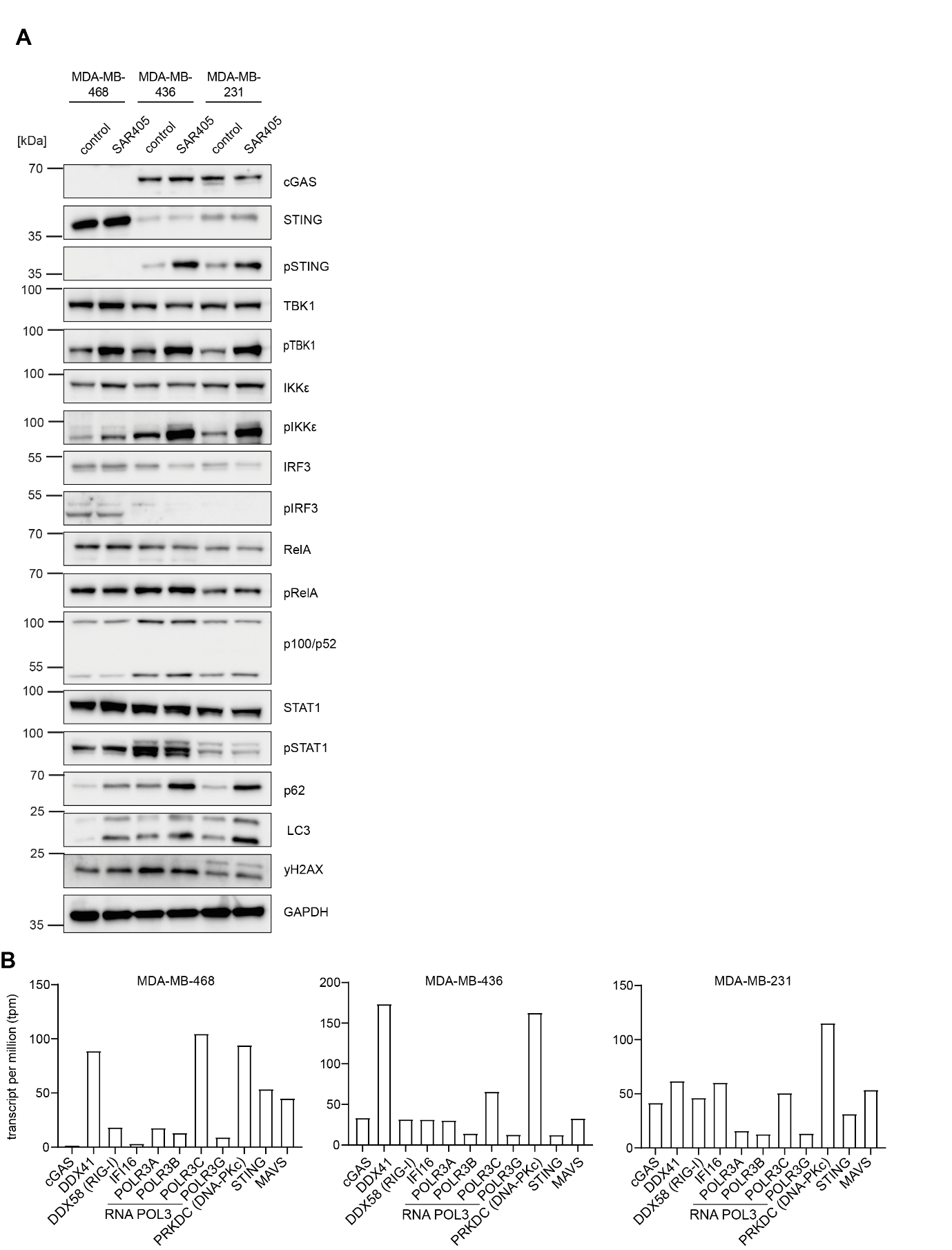


**Supplementary Fig. 6 cGAS-STING signaling in TNBC cell lines exposed to the autophagy inhibitor SAR405.** **A** Immunoblots of MDA-MB-468, MDA-MB-436 and MDA-MB-231 control cells and cells treated with 3 μM SAR405 for 48 h and harvested immediately. GAPDH was used as a loading control. Two biologically independent experiments were performed. **B** Expression of different nucleic acid sensors in the cell lines MDA-MB-468, MDA-MB-436 and MDA-MB-231 based on RNA-seq data from the Cell model passport database^69^.

**
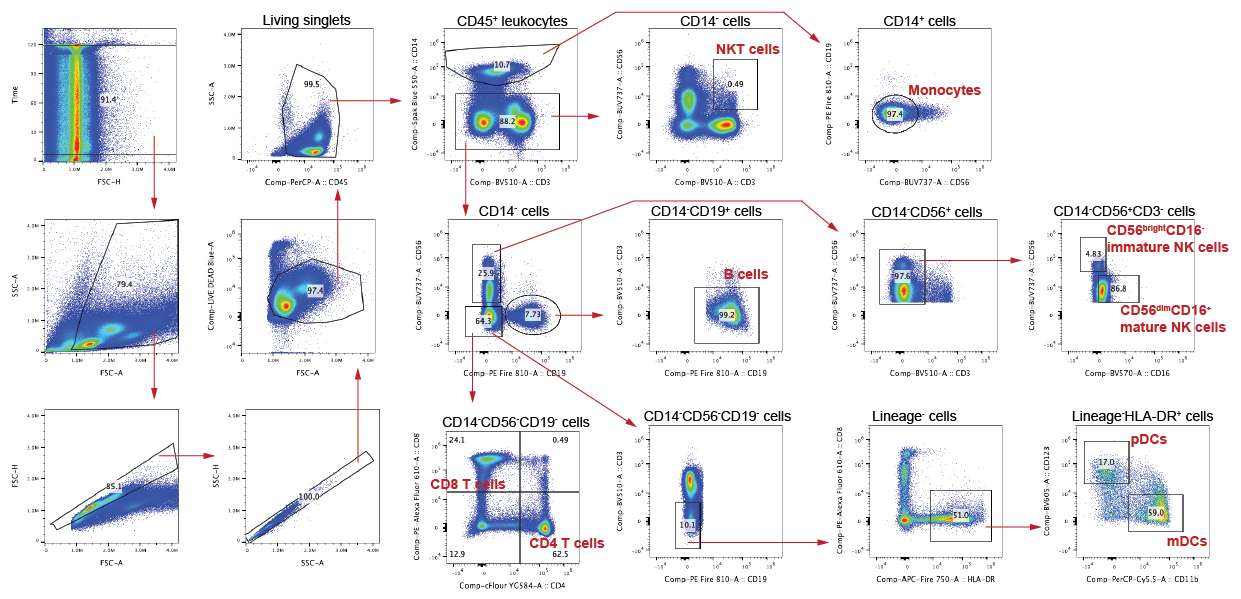
**

**Supplementary Fig. 7 Gating strategy for the identification of the immune cell subsets within peripheral blood mononuclear cells (PBMCs).** Sequential gating strategy was employed to identify viable single CD45^+^ cells that were further gated based on the expression of the lineage markers into monocytes, NKT cells, B cells, CD8 and CD4 T cells, plasmacytoid dendritic cells (pDCs) and myeloid dendritic cells (mDCs). From the two identified natural killer (NK) cell populations, only the more abundant CD56^dim^CD16^+^ mature NK cells are discussed and are referred to as ‘NK cells’.

**
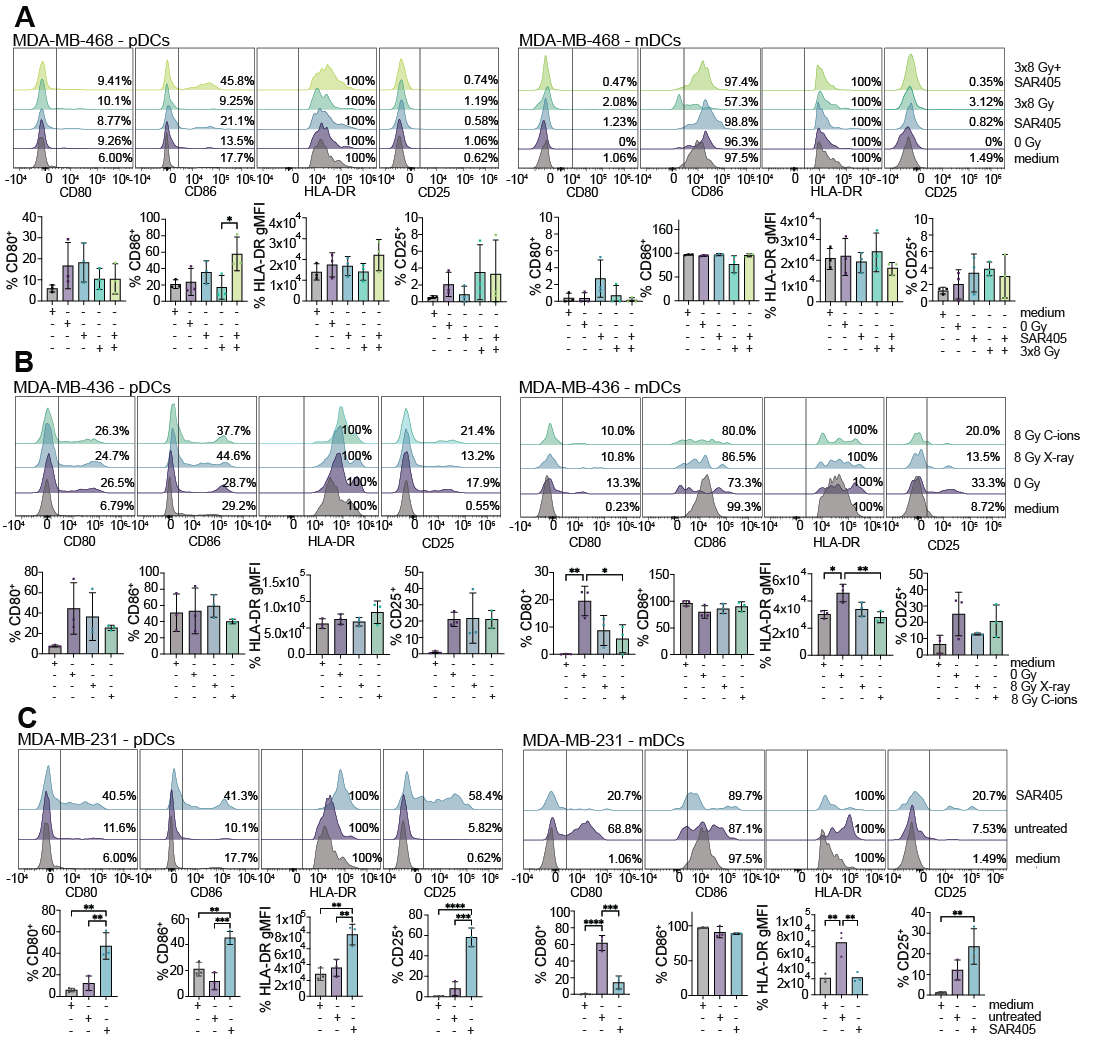
**

**Supplementary Fig. 8** **Response of human plasmacytoid dendritic cells (pDCs) and myeloid dendritic cells (mDCs) to supernatants from TNBC cells treated with the autophagy inhibitor SAR405 or radiation.** PBMCs from three different donors were incubated for 20 h in the presence of the culture medium (control) or cell-free supernatants from **A** MDA-MB-468, **B** MDA-MB-436 and **C** MDA-MB-231 cells. **A** MDA-MB-468 cells were treated with 3 μM SAR405 for 96 h, 3x8 Gy X-rays or a combination thereof. Supernatants were harvested 96 h after the last irradiation. When combined with radiation, SAR405 was added 96 h before harvesting. **B** MDA-MB-436 cells were irradiated with 8 Gy X-ray or C-ions and harvested after 96 h. **C** MDA-MB-231 cells were treated with 3 μM SAR405 for 96 h. Human pDCs (left) and mDCs (right) were identified according to lineage markers and their activation was assessed based on the analysis of activation markers CD80, CD86, HLA-DR and CD25 by spectral flow cytometry. Representative histograms of one donor (top) and percentages of the marker-positive cells ± standard deviation of three PBMC donors (bottom) are shown. In histograms, the gating for the positive cells is based on the lineage stain (depicted in gray) and is indicated by a vertical line. Since the DC lineage marker HLA-DR was used for gating, its expression was evaluated based on the geometric mean fluorescence intensity (gMFI) instead. Experiments in A and C were performed at the same time, thus they share the medium control. Statistical significance was assessed using one-way ANOVA with Tukey’s post-hoc test (*≤ 0.05; **≤ 0.01; ***≤ 0.005, ****≤0.0001).


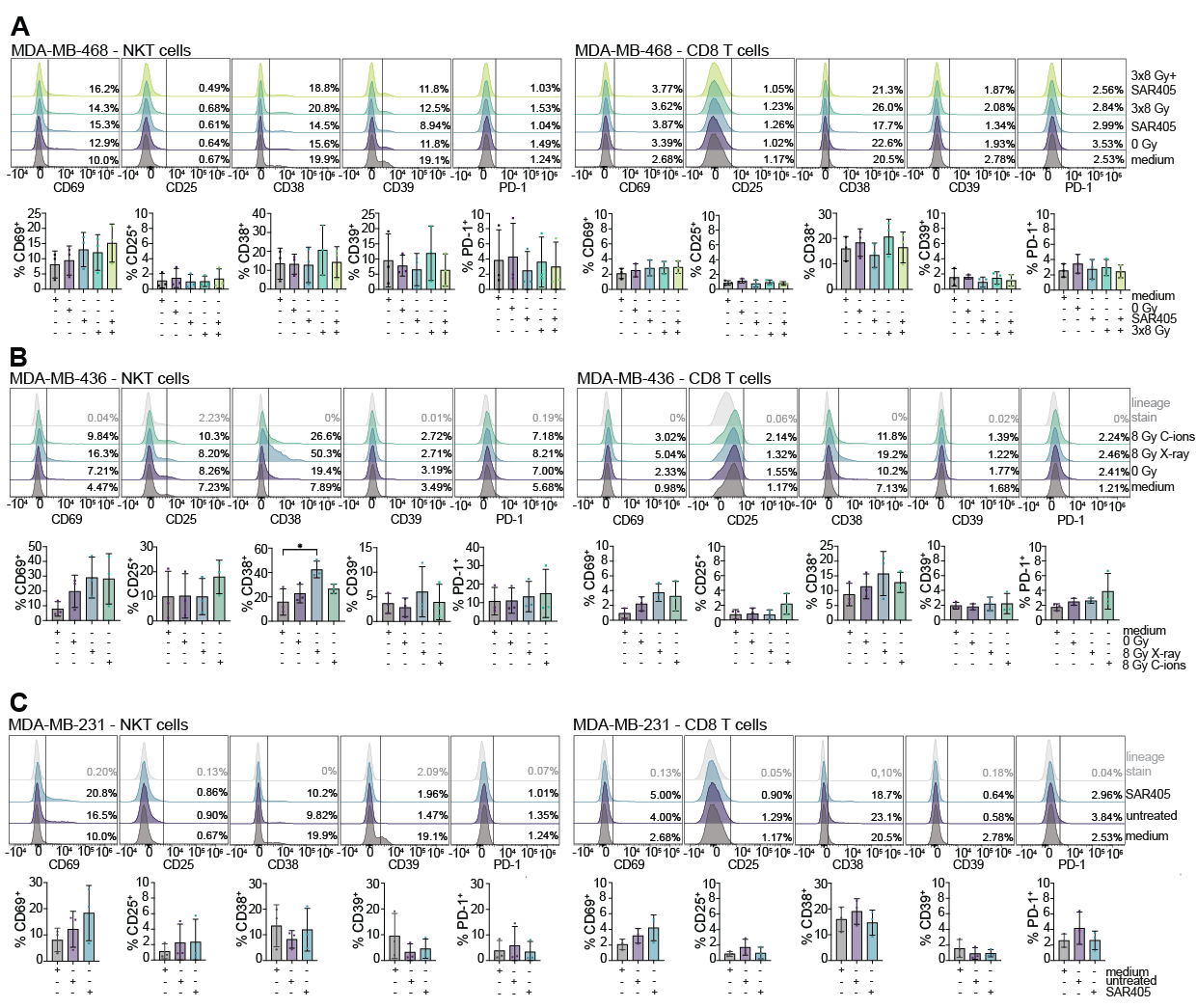


**Supplementary Fig. 9** **Response of human NKT cells and CD8 T cells to supernatants from TNBC cells treated with the autophagy inhibitor SAR405 or radiation.** PBMCs from three different donors were incubated for 20 h in the presence of the culture medium (control) or cell-free supernatants from **A** MDA-MB-468, **B** MDA-MB-436 and **C** MDA-MB-231 cells. **A** MDA-MB-468 cells were treated with 3 μM SAR405 for 96 h, 3x8 Gy X-rays or a combination thereof. Supernatants were harvested 96 h after the last irradiation. When combined with radiation, SAR405 was added 96 h before harvesting. **B** MDA-MB-436 cells were irradiated with 8 Gy X-ray or C-ions and harvested after 96 h. **C** MDA-MB-231 cells were treated with 3 μM SAR405 for 96 h. Human NKT (left) and CD8 T cells (right) were identified according to lineage markers and their activation was assessed based on the analysis of activation markers CD69, CD25, CD38 or activation-induced molecules CD39 and PD-1 with immunoregulatory functions by spectral flow cytometry. Representative histograms of one donor (top) and percentages of the marker-positive cells ± standard deviation of three PBMC donors (bottom) are shown. In histograms, the gating for the positive cells is based on the lineage stain (depicted in gray) and is indicated by a vertical line. Experiments in A and C were performed at the same time, thus they share the medium control. Statistical significance was assessed using one-way ANOVA with Tukey’s *post-hoc* test (*≤ 0.05).

**
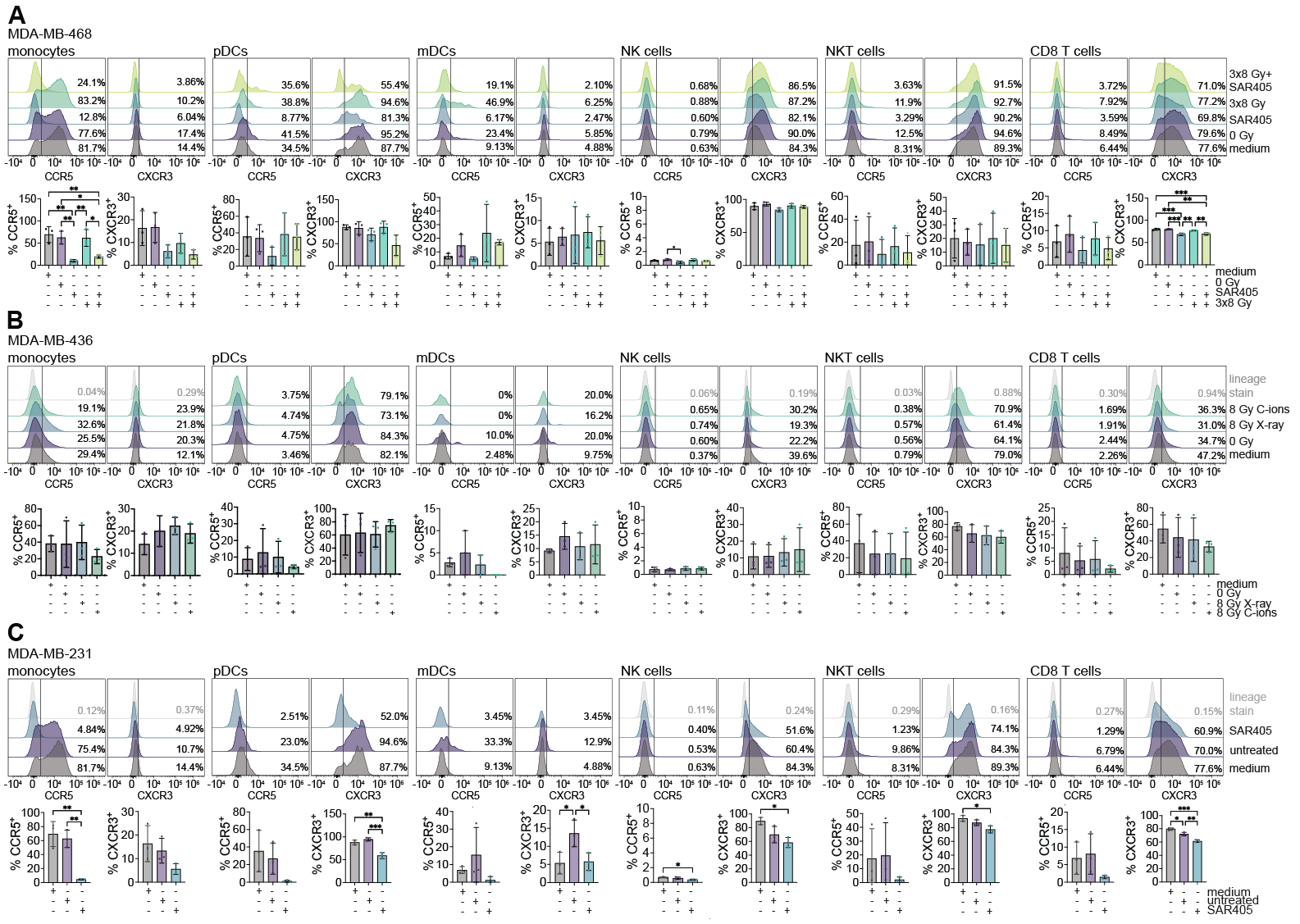
**

**Supplementary Fig. 10** **Chemokine receptor expression in PBMC subsets in response to supernatants from TNBC cells treated with the autophagy inhibitor SAR405 or radiation.** PBMCs from three different donors were incubated for 20 h in the presence of the culture medium (control) or cell-free supernatants from **A** MDA-MB-468, **B** MDA-MB-436 and **C** MDA-MB-231 cells. **A** MDA-MB-468 cells were treated with 3 μM SAR405 for 96 h, 3x8 Gy X-rays or a combination thereof. Supernatants were harvested 96 h after the last irradiation. When combined with radiation, SAR405 was added 96 h before harvesting. **B** MDA-MB-436 cells were irradiated with 8 Gy X-ray or C-ions and harvested after 96 h. **C** MDA-MB-231 cells were treated with 3 μM SAR405 for 96 h. CCR5 and CXCR3 surface expression on monocytes, pDCs, mDCs, NK, NKT and CD8 T cells was assessed using spectral flow cytometry. Representative histograms of one donor (top) and percentages of the marker-positive cells ± standard deviation of three PBMC donors (bottom) are shown. In histograms, the gating for the positive cells is based on the lineage stain (depicted in gray) and is indicated by a vertical line. Experiments in A and C were performed at the same time, thus they share the medium control. Statistical significance was assessed using one-way ANOVA with Tukey’s *post-hoc t*est (*≤ 0.05; **≤ 0.01; ***≤ 0.005).

**
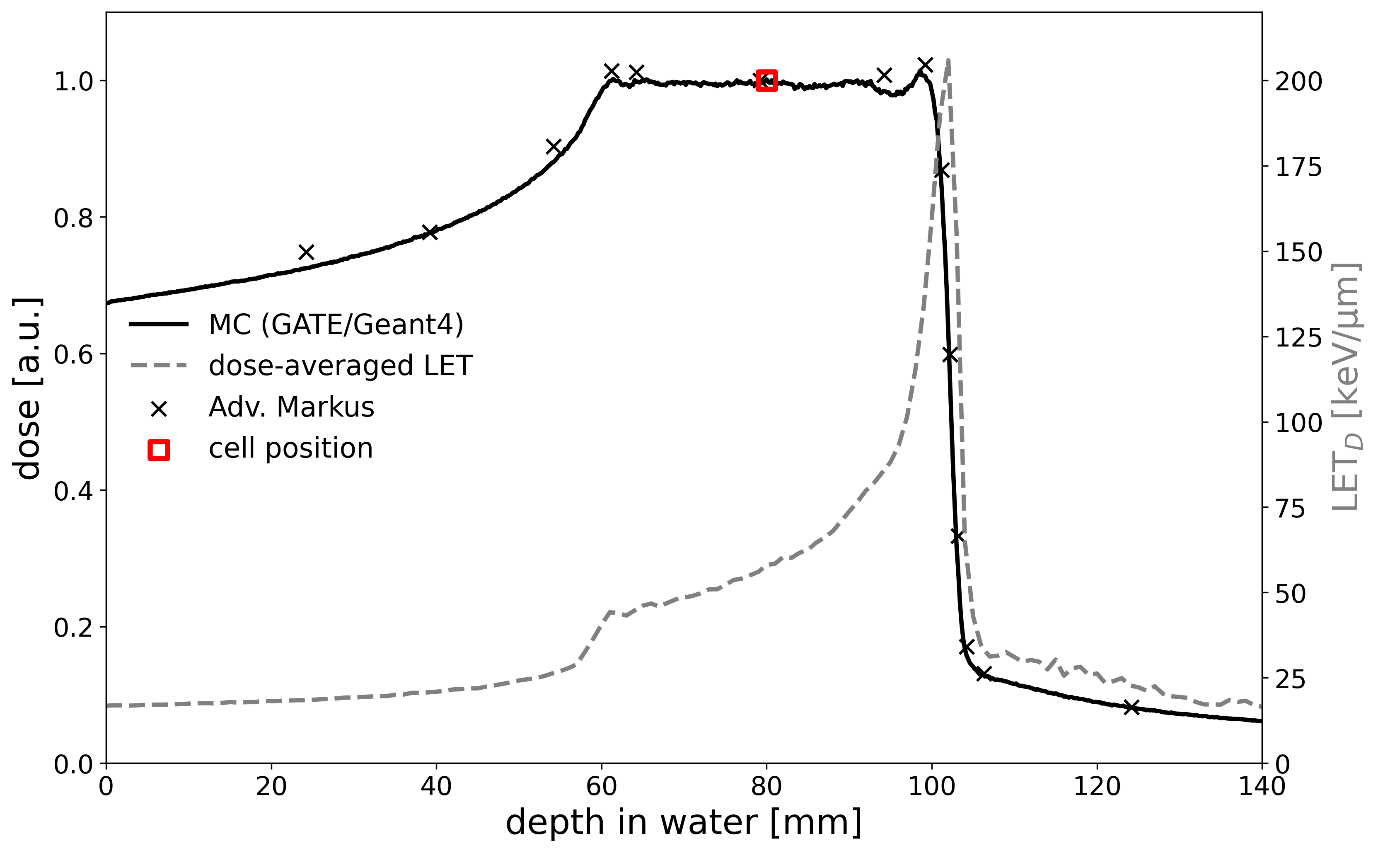
**

**Supplementary Fig. 11** The depth dose curve, dosimetry measurements, and calculated LET_D_ of the SOBP used for cell irradiations in water.

**Supplementary Table 1**. Antibodies

| Rabbit anti-GAPDH | Cell Signaling | RRID: AB_10622025; 5174S; 1:1000 for WB |
| --- | --- | --- |
| Rabbit anti-cGAS | Cell Signaling | RRID: AB_2732795; 15102S; 1:1000 for WB |
| Rabbit anti-pSTING (Ser366) | Cell Signaling | RRID: AB_2737062; 19781S; 1:1000 for WB |
| Rabbit anti-STING | Cell Signaling | RRID: AB_2732796; 13647S; 1:1000 for WB |
| Rabbit anti-pTBK1 (Ser172) | Cell Signaling | RRID: AB_10693472; 5483S; 1:1000 for WB; 1:400 for IF |
| Rabbit anti-TBK1 | Cell Signaling | RRID: AB_2827657; 38066S; 1:1000 for WB |
| Rabbit anti-pIRF3 (Ser396) | Cell Signaling | RRID: AB_823547; 4947S; 1:1000 for WB |
| Rabbit anti-IRF3 | Cell Signaling | RRID: AB_1904036; 4302S; 1:1000 for WB |
| Rabbit anti-pSTAT1 (Tyr701) | Cell Signaling | RRID: AB_561284; 9167S; 1:1000 for WB |
| Rabbit anti-STAT1 | Cell Signaling | RRID: AB_2737027; 14994S; 1:1000 for WB |
| Rabbit anti-pNF-κB p65 (Ser536) | Cell Signaling | RRID:AB_331284; 3033S; 1:1000 for WB |
| Rabbit anti-NF-κB p65 | Proteintech | RRID:AB_2178878; 10745-1-AP; 1:1000 for WB |
| Rabbit anti-pIKKε (Ser172) | Cell Signaling | RRID:AB_2737061; 8766S; 1:1000 for WB |
| Rabbit anti-IKKε | Cell Signaling | RRID:AB_1147662; 2905S; 1:1000 for WB |
| Rabbit anti-NF-κB2 p100/p52 | Cell Signaling | RRID:AB_10695537; 4882S; 1:1000 for WB |
| Rabbit anti-SQSTM1/p62 | Cell Signaling | RRID:AB_10624872; 5114S; 1:1000 for WB |
| Mouse anti-SQSTM1/p62 | BD Biosciences | RRID: AB_398152; 610833; 1:500 for IF |
| Rabbit anti-LC3A/B | Cell Signaling | RRID:AB_2617131; 12741S; 1:1000 for WB |
| Rabbit anti-γH2AX | Millipore | RRID:AB_310406; 07-164; 1:1000 for WB |
| Mouse anti-γH2AX | Millipore | RRID:AB_309864; 05-636; 1:100 for IF |
| Rabbit anti-PARP1 | Cell Signaling | RRID:AB_2160739; 9542S; 1:1000 for WB |
| Rabbit anti-FIP200 | Cell Signaling | RRID:AB 2797913; 12436; 1:1000 for WB |
| Mouse anti-dsDNA | Abcam | RRID: AB_470907; ab27156; 1:1000 for IF |
| Rabbit anti-TFAM | Genetex | RRID: AB_11176720; GTX103231; 1:500 for IF |
| Rabbit anti pATR | Cell Signaling | RRID: AB_2290281; 2853S; 1:1000 for WB |
| Mouse anti-ATR | Santa Cruz  Biotechnology | RRID: AB_2893291; sc-515173; 1:250 for WB |
| Rabbit anti-pATM | Cell Signaling | RRID: AB_2798100; 13050S; 1:1000 for WB |
| Rabbit anti-ATM | Cell Signaling | RRID: AB_2062659; 2873S; 1:1000 for WB |
| Rabbit anti-pRPA S4/S8 | Bethyl | RRID: AB_210547; A300-245A; 1:1000 for WB |
| Rabbit anti-pRPA S33 | Bethyl | RRID: AB_2180847; A300-246A; 1:1000 for WB |
| Rat anti-RPA | Cell Signaling | RRID: AB_2238543; 2208S; 1:1000 for WB |
| Rabbit anti-pCHK1 | Cell Signaling | RRID: AB_331212; 2348S; 1:1000 for WB |
| Mouse anti-CHK1 | Cell Signaling | RRID: AB_2080320; 2360S; 1:1000 for WB |
| Rabbit anti-pCHK2 | Cell Signaling | RRID: AB_331479; 2661S; 1:1000 for WB |
| Rabbit anti-CHK2 | Cell Signaling | RRID: AB_2080793; 2662S; 1:1000 for WB |
| Rabbit anti-pMLKL | Cell Signaling | RRID: AB_2732034; 91689S; 1:1000 for WB |
| Rabbit anti-MLKL | Cell Signaling | RRID: AB_2721822; 14993S; 1:1000 for WB |
| Rabbit anti-CASP8 | Cell Signaling | RRID: AB_561381; 9496S; 1:1000 for WB |
| Rabbit anti-CASP3 | Cell Signaling | RRID: AB_2070042; 9664S; 1:1000 for WB |
| Rabbit anti-COX-2 | Cell Signaling | RRID: AB_2571729; 12282S; 1:1000 for WB |
| Rabbit anit-GPX4 | Cell Signaling | RRID: AB_2924984; 52455S; 1:1000 for WB |
| Goat anti-Mouse IgG H&L (Alexa Fluor® 568) | Abcam | RRID: AB_2895153; ab175473; 1:500 for IF |
| Goat anti-Rabbit IgG H&L (Alexa Fluor® 488) | Abcam | RRID: AB_2630356; ab150077; 1:400 for IF |
| Goat anti-rabbit-AF488 | Thermo Fisher | RRID: AB_143165; A11008; 1:500 for IF |
| Goat anti-mouse-AF568 | Thermo Fisher | RRID: AB_2534072; A11004; 1:500 for IF |

**Supplementary Table 2.** Immunophenotyping panel used in spectral flow cytometry analysis of PBMCs. mAbs used in both the full immunophenotyping and the lineage panel are depicted in bold. DC – dendritic cell, Treg – regulatory T cell.

| **Marker** | **Fluorochrome** | **Clone** | **Company** | **Cat. Number** | **Final dilution** | **Purpose** |
| --- | --- | --- | --- | --- | --- | --- |
| CD45RA | BUV395 | 5H9 | BD Biosciences | 569489 | 1:460 | T cell and DC differentiation |
| Viability | LIVE DEAD Blue | - | Thermo Fisher | L23105 | 1:500 (prestain) | Viability |
| CD194/CCR4 | BUV615 | 1G1 | BD Biosciences | 613000 | 1:80 | Chemokine receptor; T cell, NK cell, monocyte differentiation |
| **CD56** | **BUV737** | **NCAM16.2** | **BD Biosciences** | **612766** | **1:70** | **NK cells** |
| CD195/CCR5 | BUV805 | 3A9 | BD Biosciences | 748872 | 1:70 | Chemokine receptor; monocyte, DC, T cell, B cell differentiation |
| CD197/CCR7 | BV421 | G043H7 | BioLegend | 353208 | 1:46 | T cell differentiation |
| CD161 | eF450 | HP-3G10 | Thermo Fisher | 48-1619-42 | 1:46 | NK cell, T cell activation marker |
| CD39 | BV480 | TU66 | BD Biosciences | 746454 | 1:140 | B cell, Treg, and monocyte differentiation |
| **CD3** | **BV510** | **SK7** | **BioLegend** | **344828** | **1:175** | **T cells, NKT cells** |
| **CD16** | **BV570** | **3G8** | **BioLegend** | **302036** | **1:140** | **Monocyte, NK cell, and DC differentiation** |
| **CD123** | **BV605** | **6H6** | **BioLegend** | **306026** | **1:46** | **Plasmacytoid DCs** |
| CD196/CCR6 | BV711 | G034E3 | BioLegend | 353436 | 1:140 | Chemokine receptor; T cell, B cell differentiation |
| **CD1c** | **BV750** | **F10/21A3** | **BD Biosciences** | **747327** | **1:280** | DC differentiation |
| CD279/PD-1 | BV785 | EH12.2H7 | BioLegend | 329930 | 1:140 | T cell inhibitory receptor |
| **CD141** | **BB515** | **1A4** | **BD Biosciences** | **565084** | **1:70** | DC differentiation |
| CD64 | FITC | 10.1 | BioLegend | 305006 | 1:280 | Myeloid cell marker |
| **CD14** | **Spark Blue 550** | **63D3** | **BioLegend** | **367148** | **1:280** | **Monocyte differentiation** |
| **CD45** | **PerCP** | **2D1** | **BioLegend** | **368506** | **1:100** | **Leukocytes** |
| CD11b | PerCP-Cy5.5 | LM2 | BioLegend | 393106 | 1:46 | DC differentiation, monocyte activation |
| **TCR γδ** | **PerCP-eFluor710** | **B1.1** | **Thermo Fisher** | **46-9959-42** | **1:280** | **γδ T cells** |
| **CD4** | **cFluor YG584** | **SK3** | **CYTEK** | **R7-20041** | **1:116** | **CD4 T cells, NKT cells** |
| **CD8** | **PE-Alexa Flour 610** | **3B5** | **Thermo Fisher** | **MHCD0822** | **1:460** | CD8 T cells, NK cells and NKT cells |
| CD80 | PE-Cy5 | L307.4 | BD Biosciences | 559370 | 1:70 | Monocyte, DC and B cell activation marker |
| CD25/IL2Rα | PE-Alexa Flour 700 | CD25-3G10 | Thermo Fisher | MHCD2524 | 1:140 | Treg marker, activation marker on T cells, B cells, myeloid cells |
| CD183/CXCR3 | PE-Cy7 | CEW33D | Thermo Fisher | 25-1839-42 | 1:200 | Chemokine receptor; DC, T, and B cell differentiation |
| **CD19** | **PE-Fire 810** | **HIB19** | **BioLegend** | **302287** | **1:280** | **B cells** |
| CD86 | APC | BU63 | BioLegend | 374208 | 1:280 | Monocyte, DC and B cell activation marker |
| CD127/IL7Rα | Spark NIR 685 | A019D5 | BioLegend | 351362 | 1:140 | T cell differentiation |
| CD69 | APC-R700 | FN50 | BD Biosciences | 565154 | 1:93 | Early activation marker expressed on leukocytes |
| HLA-DR | APC-Fire 750 | L243 | BioLegend | 307658 | 1:175 | T cell and monocyte activation, DC lineage marker |
| CD38 | APC-Fire 810 | HIT2 | BioLegend | 303550 | 1:200 | Monocyte, DC, T and B cell activation/differentiation |
